# Effectiveness of interventions for improving livelihood outcomes for people with disabilities in low‐ and middle‐income countries: A systematic review

**DOI:** 10.1002/cl2.1257

**Published:** 2022-06-30

**Authors:** Xanthe Hunt, Ashrita Saran, Lena Morgon Banks, Howard White, Hannah Kuper

**Affiliations:** ^1^ Institute for Life Course Health Research, Department of Global Health Stellenbosch University Stellenbosch South Africa; ^2^ Campbell Collaboration Vasant Kunj India; ^3^ International Center for Evidence on Disability London School of Hygiene and Tropical Medicine London UK

## Abstract

**Background:**

People with disabilities—more than a billion people worldwide—are frequently excluded from livelihood opportunities, including employment, social protection, and access to finance. Interventions are therefore needed to improve livelihood outcomes for people with disabilities, such as improving access to financial capital (e.g., social protection), human capital (e.g., health and education/training), social capital (e.g., support) or physical capital (e.g., accessible buildings). However, evidence is lacking as to which approaches should be promoted.

**Objectives:**

This review examines whether interventions for people with disabilities result in improved livelihood outcomes in low‐ and middle‐income countries (LMIC): acquisition of skills for the workplace, access to the job market, employment in formal and informal sectors, income and earnings from work, access to financial services such as grants and loans, and/or access to social protection programmes.

**Search Methods:**

The search, up to date as of February 2020, comprised of:

(1) an electronic search of databases (MEDLINE, Embase, PsychINFO, CAB Global Health, ERIC, PubMED and CINAHL),
(2) screening of all included studies in the instances where reviews were identified,
(3) screening reference lists and citations of identified recent papers and reviews, and
(4) An electronic search of a range of organisational websites and databases (including ILO, R4D, UNESCO and WHO) using the keyword search for unpublished grey to ensure maximum coverage of unpublished literature, and reduce the potential for publication bias

**Selection Criteria:**

We included all studies which reported on impact evaluations of interventions to improve livelihood outcomes for people with disabilities in LMIC.

**Data Collection and Analysis:**

We used review management software EPPI Reviewer to screen the search results. A total of 10 studies were identified as meeting the inclusion criteria. We searched for errata for our included publications and found none. Two review authors independently extracted the data from each study report, including for the confidence in study findings appraisal. Data and information were extracted regarding available characteristics of participants, intervention characteristics and control conditions, research design, sample size, risk of bias and outcomes, and results. We found that it was not possible to conduct a meta‐analysis, and generate pooled results or compare effect sizes, given the diversity of designs, methodologies, measures, and rigour across studies in this area. As such, we presented out findings narratively.

**Main Results:**

Only one of the nine interventions targeted children with disabilities alone, and only two included a mix of age groups (children and adults with disabilities. Most of the interventions targeted adults with disabilities only. Most single impairment group interventions targeted people with physical impairments alone. The research designs of the studies included one randomised controlled trial, one quasi‐randomised controlled trial (a randomised, posttest only study using propensity score matching (PSM), one case‐control study with PSM, four uncontrolled before and after studies, and three posttest only studies. Our confidence in the overall findings is low to medium on the basis of our appraisal of the studies. Two studies scored medium using our assessment tool, with the remaining eight scoring low on one or more item. All the included studies reported positive impacts on livelihoods outcomes. However, outcomes varied substantially by study, as did the methods used to establish intervention impact, and the quality and reporting of findings.

**Authors' Conclusions:**

The findings of this review suggest that it may be possible for a variety of programming approaches to improve livelihood outcomes of people with disabilities in LMIC. However, given low confidence in study findings related to methodological limitations in all the included studies, positive findings must be interpreted with caution. Additional rigorous evaluations of livelihoods interventions for people with disabilities in LMIC are needed.

## 1 PLAIN LANGUAGE SUMMARY

### 1.1 Livelihood interventions appear to improve outcomes for people with disabilities

A range of programming approaches reported improvements in livelihood outcomes for people with disabilities in low‐ and middle‐income countries (LMICs). However, confidence in study findings is low, due to methodological limitations in the research.

### 1.2 What is this review about?

More than one billion people have some form of disability. People with disabilities are frequently excluded from livelihood opportunities, including employment, social protection and access to banking and loans. Among people with disabilities over the age of 15, 36% are employed, compared to 60% for people without disabilities.

Livelihood interventions are therefore needed for people with disabilities. These include interventions aiming to improve access to financial capital (e.g., social protection), human capital (e.g., health and education/training), social capital (e.g., support) and physical capital (e.g., accessible buildings).

**What is the aim of this review?**

This review examines whether interventions for people with disabilities in LMICs result in improved livelihood outcomes, including acquisition of skills for the workplace, access to the job market, employment in formal and informal sectors, income and earnings from work, access to financial services such as grants and loans, and access to social protection programmes.

### 1.3 What studies are included?

This review includes studies that evaluate the effects of interventions on livelihood outcomes for people with disabilities in LMICs. The authors found nine interventions which used eligible study designs. Countries represented are Bangladesh, India, Nigeria, Ethiopia, Brazil, China and Vietnam. All included studies have some important methodological weaknesses.

### 1.4 What are the main findings of this review?

All included studies reported positive impacts on livelihoods outcomes. However, due to variation between studies, we did not conduct as analysis of effects across studies. As such, it is hard to draw firm conclusions about what works, for whom and how.

Most studies focused on improving access to the workplace. For example, people without disabilities were involved in programmes to improve their social attitudes to working with people with disabilities. People with certain disabilities were provided with wheelchairs. And some people with disabilities were placed in supported employment.

Studies examined the effects of vocational training programmes, a ‘motivation to work’ programme, community‐based rehabilitation and social skills training. All of these approaches showed positive impacts on livelihood outcomes, including finding employment and gaining social skills for work.

The included studies all reported that their programmes improved outcomes related to the livelihoods of people with disabilities, including acquisition of skills for the workplace, access to the job market, employment in formal and informal sectors, and access to the formal and informal social protection measures.

Future research should evaluate these approaches with more rigorous study designs. This would develop a firmer evidence base, which would also inform the delivery of interventions at scale.

### 1.5 What do the findings of this review mean?

In general, there is not a great deal of evidence on interventions to improve livelihood outcomes for people with disabilities in LMICs, so more studies are needed. Researchers should work with organisations of persons with disabilities and other non‐governmental organisations to identify priority interventions to evaluate. For instance, online and community‐based delivery of livelihood interventions could be explored, to bridge gaps in coverage of programming and reach rural populations (who were underrepresented in this review).

There are other specific research gaps that need to be filled. The geographic scope of studies should be expanded. There were no studies from Europe, Central Asia, the Middle East or North Africa.

Programmes should integrate impact evaluations to improve the evidence base. Research evaluating programmes for people with disabilities other than those with physical impairments are needed.

Overall, there is a need for more and better data to inform policy and practice, including data on a broader range of impairment types.

### 1.6 How up‐to‐date is this review?

The review authors searched for studies up to February 2020.

## 2 BACKGROUND

### 2.1 The problem, condition or issue

The United Nations Convention on the Rights of Persons with Disability defines disability as ‘long‐term physical, mental, intellectual or sensory impairments which, in interaction with various barriers, may hinder [a person's] full and effective participation in society on an equal basis with others’ (UN, 2006). More than one billion persons in the world have some form of disability (World Health Organization, 2011). This figure corresponds to about 15% of the world's population.

Disability and poverty are strongly linked. On a global level, 80% of people with disabilities live in LMIC (World Health Organization, 2011). Within countries, disability disproportionately affects the most disadvantaged sector of the population (Banks, Kuper, et al., 2017). Disability is significantly associated not only with poverty, but also lower educational attainment, lower employment rates, and worse healthcare access (Mitra et al., 2013). Consequently, scholars identify the risk of experiencing ‘multidimensional poverty’ (poverty across multiple domains) as extremely high in this population (Mitra et al., 2013). This relationship—between disability and poverty—is bidirectional, and driven by a number of factors and proposed mechanisms; for instance there are high costs associated with many of types of impairments (e.g., costs of rehabilitation), and people with disabilities are often excluded from opportunities to learn and earn, so that people with disabilities may ‘fall into’ poverty (Braithwaite & Mont, 2009; Mitra, 2018; Mitra et al., 2011, 2013; Palmer, 2011). Conversely, people who are living in poverty may be more vulnerable to injury and illness, and have worse healthcare access, and thus at increased risk of acquiring an impairment and experiencing disability (Groce et al., 2011; Palmer, 2011; Trani & Loeb, 2012).

**Figure 1:**
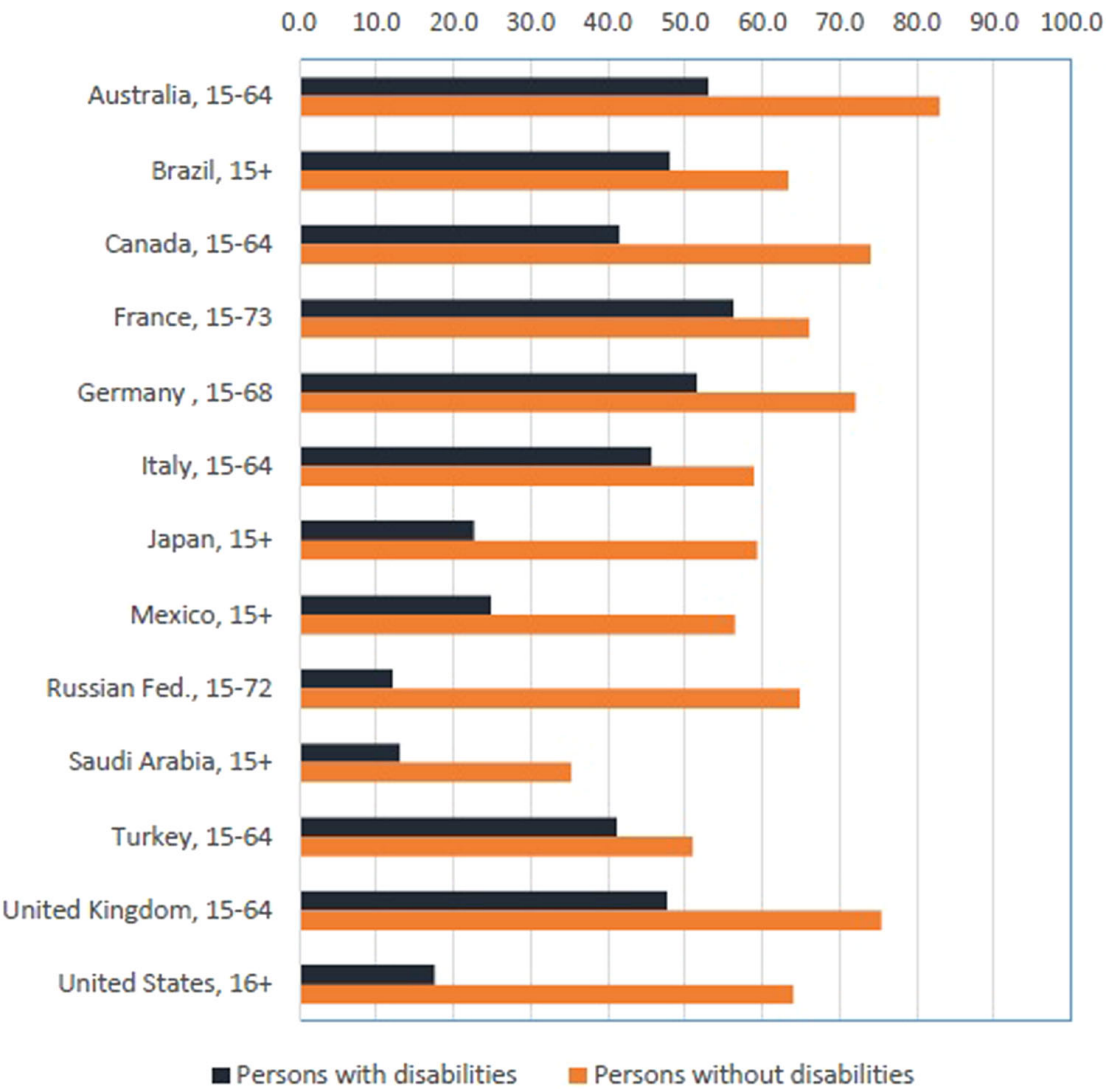
Employment‐to‐population ratio for persons with and without disabilities: Most recent data close to year 2010. *Source*: ILO (2018).

Of relevance to our review is the first of these pathways, from disability to poverty. The widespread exclusion of people with disabilities from livelihood opportunities is one of the drivers of the relationship of disability to poverty and is the focus of a substantial literature (Banks & Polack, 2014; World Health Organization, 2011). The 2018 UN Flagship Report on Disability and Development reported that across 8 geographical regions, the employment to population ratio for people with disabilities aged ≥15 years was 36% compared to 60% for people without disabilities. Indeed, an employment gap between people with and without disabilities is observed in the vast majority of countries (Mitra & Yap, 2021). The exclusion of people with disabilities from employment is also repeatedly shown in the broader literature, as illustrated in Figure 1, although these international comparisons must be made with caution due to differences in how disability and employment (especially informal employment) are measured.

There are complexities to the relationship between employment and disability. Disability is not a homogenous category and the experience of exclusion from employment will vary by gender, impairment type and context. Women already frequently face discrimination in terms of livelihood inclusion, and this may be compounded for women with disabilities (World Health Organization, 2010). For instance, the World Health Surveys used consistent methods to measure these constructs across 51 countries, and showed that employment levels were lower in men with disabilities (53%) compared to men without disabilities (65%), but that the overall level of employment was lower the gap higher when comparing women with disabilities (20%) to women without disabilities (30%) (World Health Organization, 2010). Exclusion may also vary by impairment type, as people with mental health conditions or intellectual impairments or particularly stigmatising conditions may be at higher risk of exclusion from employment (Van Beukering et al., 2021; World Health Organization, 2010), or face resistance when requesting necessary employment accommodations (Prince, 2017). Although data are lacking, people with disabilities may be particularly left behind within humanitarian settings in terms of livelihood inclusion.

Another consideration is that employment level alone is not the only pertinent measure of exclusion. Multiple studies have shown that when people with disabilities do work it is more likely to be in the informal sector, part‐time and for lower wages (Banks & Polack, 2014; World Health Organization, 2011). This pattern is illustrated by Figure 1, again with the caveat that differences in measurement of disability and employment (especially informal employment) make international comparisons difficult. The inequity in employment associated with disability occurs even though almost all jobs can be done by people with disabilities if the right supports are in place. However, it is unclear which interventions are most effective at improving employment inclusion and outcomes among people with disabilities in LMIC, and this question has not been previously explored through a systematic review.

It is important to focus beyond waged employment alone, to livelihood more broadly. Livelihood encompasses the means through which individuals or households can meet their basic needs. It encompasses people's capabilities (Sen, 1993), assets, income and activities required to secure the necessities of life (Hebinck & Bourdillon, 2001). A livelihood is sustainable when it can cope with, and recover from, stress and shocks, and when it can maintain or enhance its capabilities and assets both now and in the future, while not undermining the natural resource base (Chambers & Conway, 1991). Livelihood, therefore, also includes social protection and financial support, as well as individual's skills to be included in employment.

Social protection includes programmes and policies designed to reduce poverty and vulnerability, for instance, by providing social assistance or by promoting efficient labour markets. Social protection can therefore assure that low‐income and vulnerable populations are able to maintain a basic livelihood, including people with disabilities. Indeed, many countries offer a disability allowance or similar scheme (Walsham et al., 2019). In Korea, for instance, there is a means‐tested and noncontributory public assistance grant, called the National Basic *Livelihood* Security System (NBLSS) (emphasis added) (Jeon et al., 2017). The aim of this grant is to support livelihoods—to mitigate poverty and improve the quality of life and capacity to maintain a minimal standard of living, for the low‐income families and vulnerable groups (including people with disabilities) (Jeon et al., 2017). Social protection interventions need to address the inequalities and the processes of social exclusion that people with disabilities face in attaining a livelihood to have a meaningful impact on their livelihood (de Haan, 2017; Stienstra & Lee, 2019). Yet, evidence is lacking on whether social protection or other similar interventions are effective at improving livelihoods for people with disabilities, as most studies have focused on interventions to improve waged employment alone (Banks, Mearkle, et al., 2017; Cramm & Finkenflugel, 2008).

**Figure 2:**
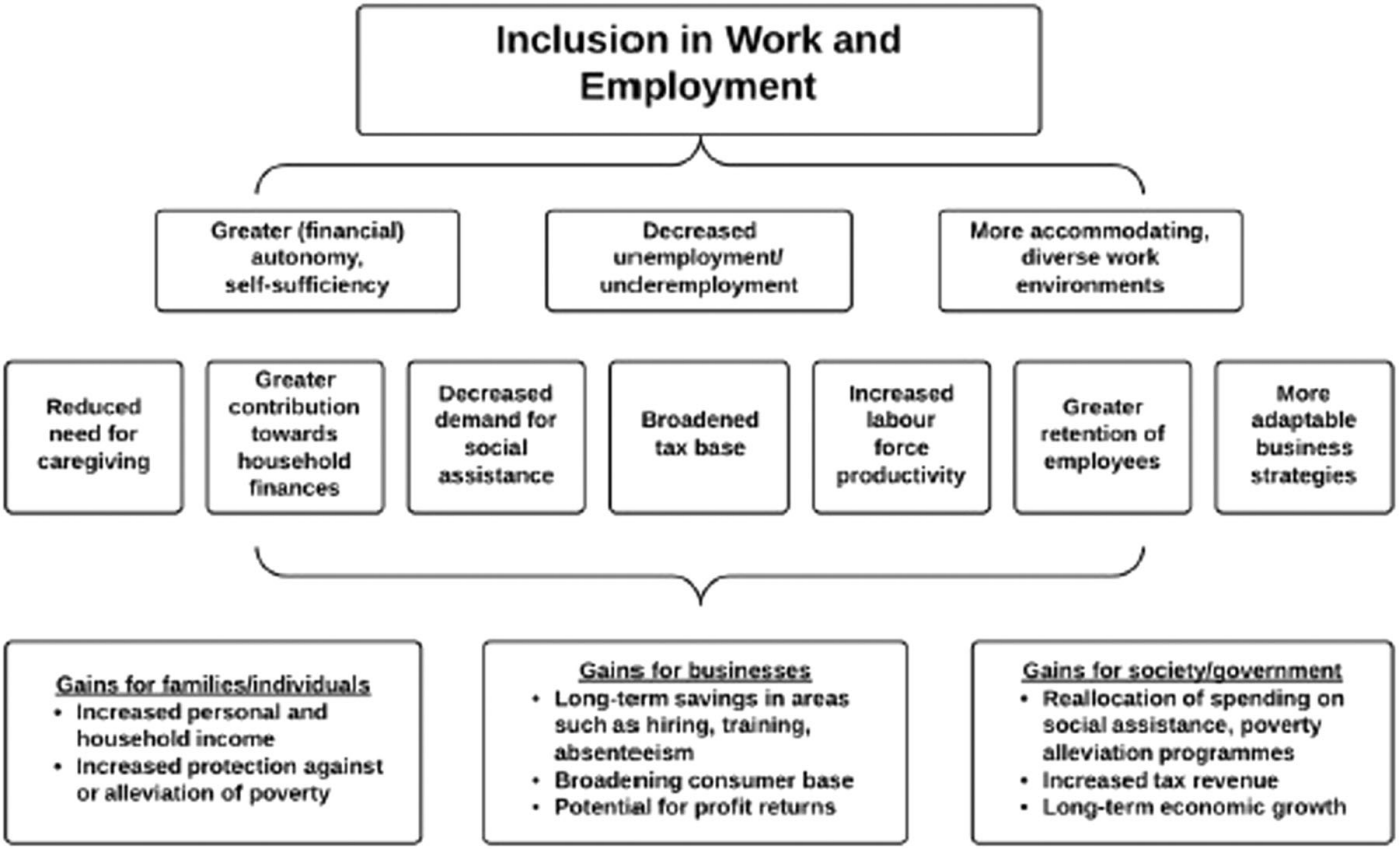
How livelihood can reap gains for people with disabilities. *Source*: Banks and Polack (2014).

The financial benefits for people with disabilities of inclusion in livelihood opportunities are obvious (Figure 2) (Banks & Polack, 2014). Improving livelihood outcomes will help people to meet their basic needs. People who are employed will earn income, whether financial or in kind, which will reduce their poverty levels. These benefits will extend beyond the individual to his/her household, as they contribute to the household economy. Financial benefits are also reaped by employers, as they are able to select employees from the full range of skills and abilities, and as evidence suggests that people with disabilities may be particularly loyal and committed employees (UNenable, 2007). Society will also see financial benefits through tax generated from the salary of people with disabilities (Deloitte, 2011). For instance, a report commissioned in 2011 by the Australian Network on Disability showed that closing the gap between labour market participation rates and unemployment rates for people with and without disabilities by one‐third would increase Australia's GDP by $43 billion over the following 10 years (Deloitte, 2011).

The nonfinancial benefits of improving livelihood opportunities for people with disabilities must also be emphasised (Figure 2). Employment is a cornerstone of social inclusion and facilitates friendship and engagement in society. It also promotes human dignity and social cohesion. Fulfilling the right to livelihood inclusion may also help other rights to be met—for instance, the workplace is a key provider of healthcare, and receipt of social protection may help health care and educational costs to be met (as evidence from populations of people without disabilities suggests (Case et al., 2005; Tembo & Freeland, 2008).

#### 2.1.1 The intervention

The intervention considered in this review are those that improve livelihood outcomes for people with disabilities. We used the WHO's Community Based Rehabilitation (CBR) Guidelines (World Health Organization, 2010) as our starting point for conceptualising the kinds of interventions which may be considered as livelihood interventions. CBR, which is promoted by the WHO to improve the lives of people with disabilities, has ‘livelihood’ as one of its five pillars (World Health Organization, 2010). Within the ‘livelihood’ pillar, there are five specific components which we used to initially elaborate a list of the types of interventions which might be included in this review: wage employment, skills development, self‐employment, access to financial services (e.g., micro‐credit schemes, access to bank accounts), and inclusion in social protection programmes. Each of these categories has specific interventions which are named in Table 1 (e.g., vocational training, job placements, and birth registration). However, given that our review is not only concerned with CBR programmes, the CBR served only as a guiding framework for the intervention categories, these were piloted and refined against a set of studies before use. During this process, we added two categories to the livelihood pillar, namely Health and Rehabilitation, and Policies, as potential approaches to improve livelihood inclusion.

**Table 1:** Categories of interventions to improve livelihood outcomes for people with disabilities

Intervention category	Intervention subcategory
Skills development	Training opportunities for employment such as vocational training
	Access to basic educational opportunities
	Social and communications skills training
	Business skills training
Self‐employment	Agricultural or nonagricultural
Waged employment	Apprenticeships
	Job searching services
	Overcome physical and social barriers to the workplace
	Job placement
Financial services	Access to credit
	Savings and loans initiatives
Social protection	Health and social insurance schemes
	Cash transfers, in kind transfers (e.g., food for work programmes)
	Birth registration
	Social assistance intervention
Health and rehabilitation	Health and rehabilitation
	Assistive technology
Policies	International legislation (e.g., universal declaration of human rights)
	Employment policies (e.g., antidiscrimination, quotas, or accessible buildings)

We considered interventions that specifically target people with disabilities, as well as mainstream programmes that are inclusive of people with disabilities and present disaggregated outcomes for people with disabilities.

#### 2.1.2 How the intervention might work

It is important to consider the barriers to livelihood opportunities experienced by people with disabilities, to identify how these may be overcome. People with disabilities are not a homogenous group, and the reasons for exclusion will vary for women and men, in different settings, and for people with different impairment types. Nevertheless, barriers can be broadly categorised as being experienced at the level of the System, the Programme (Workplace), and the Individual (the Family or the Person) (Wapling, 2016).

*System‐level barriers* include the lack of legislation or policies to support the inclusion of people with disabilities in livelihood opportunities. Even where there are good policies, these may not be implemented due to failure to monitor inclusion or to implement incentives or penalties to promote inclusion. Another important concern is inadequate resource allocation to support inclusion (e.g., lack of funds for access to work schemes). Policies may also be inappropriately formulated so that they penalise people with disabilities who work (e.g., create a benefits trap) or establish over‐protective labour laws that discourage firms from employing people with disabilities.

*Programme‐level barriers* include lack of reasonable accommodation (including assistive technology), physical accessibility of the workplace, transport or toilets, or the existence of negative attitudes from employers and co‐workers towards people with disabilities. Programmes, such as micro‐credit schemes, may also explicitly exclude people with disabilities (e.g., making people with long‐term health conditions ineligible).

*Individual‐level barriers* include the frequently lower level of training or skills of people with disabilities, following their higher risk of exclusion from education, which may make livelihood opportunities more difficult to obtain. People with disabilities may also experience poor health, and require treatment and rehabilitation, which can make full‐time employment more challenging. Depending on the impairment type, people with disabilities may have difficulties with different skills needed in many work environments, such as concentrating and controlled behaviour, and this may reinforce negative attitudes that people with disabilities are not capable of learning or worth investing in. People with disabilities may experience higher costs of working (e.g., need for accessible transport), which creates a barrier to entry into the labour force. Attitudinal barriers may also be important, for instance if relatives discourage a person with disabilities from working in attempts to be protective or if people with disabilities themselves hold negative attitudes through internalising societal stereotypes.

Approaches to improve livelihood inclusion and outcomes for people with disabilities must act by targeting the barriers that they experience. In other words, they must operate at the level of the system (e.g., improving policy and legislation), the programme (e.g., making reasonable accommodations) and/or individual (e.g., providing training in new skills). These interventions should address inclusion in livelihood opportunities in the broadest sense, and not focus on employment alone. The World Report on Disability describes different approaches to addressing barriers and thereby enhancing livelihood opportunities (World Health Organization, 2011).

At the *systems‐level*, most countries have laws and regulations in place protecting people with disabilities from discrimination in employment,^1^ but they should be implemented where they are lacking or improved if they are inadequate. Systems‐level interventions may also include instituting requirement for reasonable accommodation in the workplace, implementation of quotas for employment of people with disabilities, establishment of tax incentives to employers, mainstreaming disability into public employment services, or promotion of affirmative action. A concern is that regulations can act as disincentive to the employment of people with disabilities (e.g., due to expense of providing specialist resources, of strong protection of workers' rights), and this must be avoided.

Examples of *programme‐level* interventions include supported employment (e.g., specialist job training, social firms), sheltered employment (e.g., employment in segregated facilities), social protection (e.g., disability grants), and micro‐finance (e.g., group loans or small business loans).

*Individual‐level* interventions include activities such as vocational rehabilitation programmes, which aim to restore the capabilities of people with disabilities so that they can participate in a competitive labour market, or other forms of skill development. Health and rehabilitation initiatives which facilitate working among people with disabilities are also relevant here. Efforts to change attitudes are also important, so that people with disabilities believe that they are as capable of productive work.

#### 2.1.3 Why it is important to do this review

The United Nations Convention on the Rights of Persons with Disabilities (UNCRPD) recognises the rights of people with disabilities to work and employment (article 27), including the ‘opportunity to gain a living by work freely chosen and accepted in a labour market and work environment that is open, inclusive and accessible to persons with disabilities’ (UN, 2006). This article also refers to the rights of persons with disabilities to access technical and vocational training, opportunities for self‐employment and entrepreneurship, and a good working environment that provides reasonable accommodation. Article 28 of the UNCRPD asserts the rights of persons with disabilities to accessing social protection programmes and poverty reduction programmes.

The Sustainability Development Goals (SDGs) are also relevant to this issue (UN, 2015). SDG1 is to ‘End poverty in all its forms everywhere’ and includes a specific target to ‘Implement nationally appropriate social protection systems and measures for all’ (emphasis added). Furthermore, SDG 8 is to ‘Promote sustained, inclusive and sustainable economic growth, full and productive employment and decent work for all’. This goal is ambitious as ‘decent work for all’, according to the International Labour Organisation (ILO), means opportunities for work that are productive and deliver a fair income, security in the workplace and social protection for families, better prospects for personal development and social integration, freedom for people to express their concerns, organise and participate in the decisions that affect their lives and equality of opportunity and treatment for all women and men (ILO, 2018). ‘Sustained’ and ‘sustainable economic growth’ places emphasis on long‐term endurance. Finally, ‘inclusive’ requires opportunities for work to be equal for different groups, and SDG8 explicitly states ‘including for persons with disabilities’.

Development initiatives also prioritise inclusive livelihood. For instance, Community Based Rehabilitation (CBR) is promoted by the WHO to improve the lives of people with disabilities, and it has ‘livelihood’ as one of its main pillars (World Health Organization, 2010). The focus on livelihood includes wage employment, but also includes skills development, self‐employment, access to financial services (e.g., micro‐credit schemes), and inclusion in social protection programmes.

In addition, most countries have policies in place protecting people with disabilities from discrimination in employment specifically. Recent examples include the Law on the Rights of Persons with Disabilities adopted in India in 2016 and Indonesia Law no. 8/2016 on Persons with Disabilities. Extensive policies are also in place promoting livelihood opportunities for people with disabilities.

However, existing research does not provide clear conclusions regarding which interventions are effective to improve livelihood outcomes for people with disabilities in LMIC; nor whether interventions appear effective for different categories of disability. Furthermore, evidence on which interventions are effective to achieve the specified policies have not been systematically reviewed. Several systematic reviews and protocols do exist that are relevant to the topic, but none which would address the stated objectives of this review.

Two relevant Campbell reviews have been completed. Iemmi et al sought to assess the effectiveness of CBR for people with disabilities in LMIC, but interventions to improve livelihood outcomes that do not operate through CBR were not included in this review (Iemmi et al., 2015). Tripney et al assessed the effectiveness of interventions to improve the labour market situation of adults with physical and/or sensory disabilities in LMIC (Tripney et al., 2015). This review identified 14 eligible studies, which generally found positive impacts of the interventions, despite concerns about the quality of the data. While this latter review is relevant to the current proposed review, it did not include interventions aimed at people with psychosocial disabilities, nor did it address broader livelihood outcomes (e.g., social protection, access to financial services). There are also likely to be relevant papers published since these reviews were undertaken.

There is a broader existing pool of reviews which focus on specific aspects of the central question of which interventions are effective at improving livelihood outcomes for people with disabilities. These reviews are restricted in terms of:

– *Impairment type/condition included*: Several reviews have been undertaken, or are planned, which focus on livelihood outcomes for people with specific impairments or conditions, including people with musculoskeletal conditions (Alexander et al., 2017; Seeberg et al., 2019), Autism, (Westbrook et al., 2013) acquired brain injury (Batavia et al., 2017), Stroke (Chan et al., 2013) or mental health conditions (Suijkerbuijk et al., 2017). However, reviews are lacking addressing disability holistically.
– *Eligible livelihood outcomes*: Reviews have been undertaken or are planned that focus only on restricted outcomes related to livelihood. As an example, Gensby et al. addressed the effectiveness of workplace‐based disability management programmes for promoting return‐to‐work outcomes (Gensby et al., 2012), while Alexander et al. focused on work participation (Alexander et al., 2017). Banks et al. considered studies on what is effective to improve inclusion in social protection programmes for people with disabilities (Banks, Kuper, et al., 2017). Here too, data are lacking despite the fact social protection programmes and financial schemes are widely promoted globally in efforts to alleviate poverty.
– *Other socio‐demographic restrictions*: Several reviews exist focused only on interventions for young adults (Jetha et al., 2019).
– *Geographic location*: Most existing reviews have either identified no eligible studies (e.g., Westbrook et al., 2013), or only studies from high income settings (e.g., Gensby et al., 2012 or Jetha et al., 2019).

There is consequently a need for a review assessing the overall literature on effectiveness of interventions to improve livelihood for people with disabilities, including broad livelihood outcomes. This review should be focused on LMIC, as this is where 80% of people with disabilities live and the particular challenges and opportunities with respect to livelihood may differ from high‐income settings.

## 3 OBJECTIVES

The question posed by this review was ‘What works to improve livelihood outcomes for people with disabilities in LMIC?’. The objectives of this review were to answer the following research questions:

1. What is the effect size of the effectiveness of interventions to improve livelihood outcomes for people with disabilities in LMIC, and what is the quality of the evidence base?
2. What works to improve livelihood outcomes for people with disabilities in LMIC?
3. Which interventions appear most effective for different categories of disability?
4. What are the barriers and facilitators to the improvement of livelihood outcomes to people with disabilities?

## 4 METHODS

### 4.1 Criteria for considering studies for this review

#### 4.1.1 Types of studies

Eligible study designs were defined on the basis of being an impact evaluation. Descriptive studies of various designs and methodologies were not included because they, unlike impact evaluations, cannot address the question of effect. To answer the question posed by this review ‘What works to improve livelihood outcomes for people with disabilities in LMIC?’, we required quantitative evidence of effect.

Eligible designs included those in which one of the following was true:

a) participants were randomly assigned (using a process of random allocation, such as a random number generation),
b) a quasi‐random method of assignment was used,
c) participants were non‐randomly assigned but matched on pre‐tests and/or relevant demographic characteristics (using observables, or propensity scores) and/or according to a cut‐off on an ordinal or continuous variable (regression discontinuity design),
d) participants were non‐randomly assigned, but statistical methods have been used to control for differences between groups (e.g., using multiple regression analysis or instrumental variables regression),
e) the design attempted to detect whether the intervention has had an effect significantly greater than any underlying trend over time, using observations at multiple time points before and after the intervention (interrupted time‐series design),
f) participants receiving an intervention were compared with a similar group from the past who did not (i.e., a historically controlled study), or
g) observations were made on a group of individuals before and after an intervention, but with no control group (single‐group before‐and‐after study).

#### 4.1.2 Types of participants

The target population were people with disabilities living in LMIC, including people with physical, sensory, intellectual, cognitive, and psychosocial (i.e., arising from a mental health condition) impairments. We also included studies which were concerned with family members or carers of people with disabilities, and service providers working with people with disabilities, in LMIC (although these studies were only included where a relevant livelihood outcome among people with disabilities was included). Population sub‐groups of interest included women, children (particularly vulnerable children, e.g., those in care), different impairment groups, conflict and post‐conflict settings, migrants/refugees/internally displaced people, and ethnic minority groups. The LMIC context, and opportunities for people with disabilities, are considerably different from those in high‐income countries, hence the need for a separate review.

#### 4.1.3 Types of interventions

The WHO CBR matrix served as a guiding framework for the intervention and outcome categories, as described above. There were no restrictions on comparators/comparison groups, however, a study must have *both* an eligible intervention *and* an eligible outcome to be included. Eligible intervention types related to livelihoods, targeted at the system‐, programme‐ and/or individual‐level, and are presented in Table 1.

#### 4.1.4 Types of outcome measures

Eligible outcomes were also developed from those included in the livelihood pillar of the CBR matrix. All outcomes were relevant regardless of whether they were primary outcomes, or secondary outcomes of the study. It is important to note that if a study did not have *both* an eligible intervention *and* an eligible outcome then it was excluded. The outcomes of interest included those experienced at the system‐, programme‐ and/or individual‐level, and are presented in Table 2.

**Table 2:** Categories of livelihoods outcomes

Outcome domain	Outcome subcategory	Examples
Acquisition of skills for the workplace	Technical skills	People with disabilities acquire technical skills for work through, for example, training in typing, coding or use of specific machinery.
	Business skills	People with disabilities acquire business skills, through, for example, courses in book‐keeping, entrepreneurship training, marketing training, or business management.
	Social and communication skills relevant to employment	People with disabilities acquire the social and communication skills necessary to succeed in the workplace, though, for instance, social skills training, or courses in workplace communication.
	Basic educational competencies relevant to employment	People with disabilities acquire competencies relevant to employment such as knowledge of accounting or economics.
Access to job market	People with disability can engage in job identification and application	People with disabilities are supported to find and apply for jobs and/or identify business opportunities and start their own ventures.
	Physical and social barriers to employment are removed (e.g., negative attitudes or employers)	Employers and employees acquire improved skills and knowledge about disability, facilities are made accessible, and/or reasonable accommodations are put in place.
Employment in formal and informal sector	Self‐employment, entrepreneurship and/or informal sector participation	People with disabilities engage in work of their choosing through self‐employment, entrepreneurship and/or informal sector participation.
	Waged/salaried employment and formal sector participation	People with disabilities engage in waged/salaried employment and/or participate in the formal sector.
Income and earnings from work	Work in the formal and/or informal sector	Men and women with disability have paid and decent work in the formal and/or informal sector
	Income is earned through people with disabilities' own chosen economic activities	Women and men with disability earn income through their own chosen economic activities
Access to financial services such as grants and loans	Grants, loans, and other financial services	Men and women with disability have access to grants, loans, and other financial services
	Savings and credit schemes	Men and women with disability participate in local saving and credit schemes
Access to social protection programmes	Access to social protection programmes	Men and women with disability access formal and informal social protection programmes

#### 4.1.5 Duration of follow‐up

Any duration of follow‐up was included.

#### 4.1.6 Types of settings

All settings were eligible, provided that the study is situated within a low‐ and‐middle‐income country, as defined by the World Bank (https://datahelpdesk.worldbank.org/knowledgebase/articles/906519-world-bank-country-and-lending-groups).

### 4.2 Search methods for identification of studies

This systematic review was based on evidence already identified in the Evidence and Gap Map (EGM) commissioned by the Commonwealth and Development Office (FCDO) under its support for the Centre for Excellence for Development Impact and Learning (CEDIL) and PENDA grant from FCDO for the support and published report by White, Saran, and Kuper (Saran et al., 2020). The EGM present studies on the effectiveness of interventions for people with disabilities in LMIC. We updated the database search and screened the references to identify additional studies. This review was based on the updated searches performed for the map February 2020. As the inclusion/exclusion criteria for this review were narrower in scope than the scope of the EGM, the review team independently screened all studies included in the map to meet the predetermined eligibility criteria outlined previously. In April 2022 we also updated our searches using Open Alex in EPPI‐reviewer to ensure that nothing had been missed, adding livelihoods‐specific search terms to our search strategy using the Campbell Collaboration's machine learning system for identifying studies. This process did not yield any additional studies within the time frame of this review (i.e., before February 2020).

The search comprised (1) an electronic search of databases and sector‐specific websites, (2) screening of all included studies in the instances where reviews are identified, (3) and citation searching of included reviews (including both forward and backward searching).

#### 4.2.1 Electronic searches

A search of the following electronic databases was conducted by the author:

- MEDLINE(R)
- Embase Classic+Embase
- PsycINFO
- CAB Global Health
- CINAHL
- ERIC
- Scopus
- Web of Science (Social Sciences Citation Index)
- WHO Global Health Index

MEDLINE, Embase, PsychINFO, and CAB Global Health were searched through OVID and ERIC and CINAHL through Ebsco. PubMED through NCBI.

Search strategies were tailored for each of the databases (see Supporting Information: Annex 2). No restrictions were placed. The main search strategy was as follows, using English as the search language:

**POPULATION**: (disable* or disabilit* or handicapped) **OR** (physical* or intellectual* or learning or psychiatric* or sensory or motor or neuromotor or cognitive or mental* or developmental or communication or learning) **OR** (cognitive* or learning or mobility or sensory or visual* or vision or sight or hearing or physical* or mental* or intellectual*) adj2 (impair* or disabilit* or disabl* or handicap*) **OR** (communication or language or speech or learning) adj5 (disorder*) **OR** (depression or depressive or anxiety or psychiat* or well‐being or quality of life or self‐esteem or self perception) adj2 (impair* or disabilit* or disabl* or handicap*) **OR** mental health **OR** (schizophreni* or psychos* or psychotic or schizoaffective or schizophreniform or dementia* or alzheimer*) adj2 (impair* or disabilit* or disabl* or handicap*) **OR** (mental* or emotional* or psychiatric or neurologic*) adj2 (disorder* or ill or illness*) **OR** (autis* or dyslexi* or Down* syndrome or mongolism or trisomy 21) **OR** (intellectual* or educational* or mental* or psychological* or developmental) adj5 (impair* or retard* or deficien* or disable* or disabili* or handicap* or ill*) **OR** (hearing or acoustic or ear*) adj5 (loss* or impair* or deficien* or disable* or disabili* or handicap* or deaf*) **OR** (visual* or vision or eye* or ocular) adj5 (loss* or impair* or deficien* or disable* or disabili* or handicap* or blind*) **OR** (cerebral pals* or spina bifida or muscular dystroph* or arthriti* or osteogenesis imperfecta or musculoskeletal abnormalit* or musculo‐skeletal abnormalit* or muscular abnormalit* or skeletal abnormalit* or limb abnormalit* or brain injur* or amput* or clubfoot or polio* or paraplegi* or paralys* or paralyz* or hemiplegi* or stroke* or cerebrovascular accident*) adj2 (impair* or disabilit* or disabl* or handicap*) **OR** (physical* adj5 (impair* or deficien* or disable* or disabili* or handicap*) **OR** people with disabilities/or children with disabilities/or people with mental disabilities/or people with physical disabilities/**OR** abnormalities/or exp congenital abnormalities/or exp deformities/or exp disabilities/or exp malformations/**OR** exp mental disorders/or exp mental health/or learning disabilities/or paralysis/or paraparesis/or paraplegia/or poliomyelitis/or hearing impairment/or deafness/or people with hearing impairment/or vision disorders/or blindness/or people with visual impairment/

**STUDY DESIGN**: (controlled clinical trial/or randomised controlled trial/or equivalence trial/or pragmatic clinical trial/or case‐control studies/or retrospective studies/or cohort studies/or follow‐up studies/or longitudinal studies/or prospective studies/or epidemiologic methods/or epidemiologic studies/or controlled before‐after studies/or cross‐sectional studies/or interrupted time series analysis/or control groups/or cross‐over studies/or double‐blind method/or matched‐pair analysis/or meta‐analysis as topic/or random allocation/or single‐blind method/or ‘retraction of publication’/or case reports/**OR** (random or placebo or single blind or double blind or triple blind or cohort or ((case or cohort or follow up or follow‐up) adj2 (control or series or report or study or studies)) or retrospective or (observ adj3 (study or studies)))

**LOCATION**: Developing Countries **OR** Africa/or Asia/or Caribbean/or West Indies/or Middle East/or South America/or Latin America/or Central America/**OR** (Africa or Asia or Caribbean or West Indies or Middle East or South America or Latin America or Central America) **OR** ((developing or less* developed or under developed or underdeveloped or middle income or low* income or underserved or under served or deprived or poor*) adj (countr* or nation? or population? or world or state*)) **OR** ((developing or less* developed or under developed or underdeveloped or middle income or low* income) adj (economy or economies)) **OR** (low* adj (gdp or gnp or gross domestic or gross national)) **OR** (low adj3 middle adj3 countr*) **OR** (lmic or lmics or third world or lami countr*) **OR** transitional countr*

#### 4.2.2 Searching other resources

We searched the reference lists of identified recent papers and reviews. To ensure maximum coverage of unpublished literature, and reduce the potential for publication bias, we searched the following organisational websites and databases using the keyword search for unpublished grey:

- ILO
- FCDO (including Research for Development [R4D])
- UNESCO
- WHO
- Disability Programme of the United Nations Economic and Social Commission for Asia and the Pacific (UNSCAP)
- United States Agency for International Development (USAID)
- Dissertation Abstracts, Conference Proceedings and Open Grey.
- Humanity and Inclusion (HI) http://www.hi-us.org/publications
- CBM https://www.cbm.org/Publications-252011.php
- Plan international https://plan-international.org/publications

### 4.3 Data collection and analysis

#### 4.3.1 Description of methods used in primary research

We used EPPI Reviewer (https://eppi.ioe.ac.uk/) to screen the search results. Due to time and resource constraints, at the title and abstract stage, we used EPPI reviewer's machine learning capabilities to prioritise studies in order of likelihood of inclusion. We used single screening at this stage. Two researchers independently screened at full‐text stage with an agreement rate of 92.8% (Cohen's *k*: 0.758).

**Figure 3:**
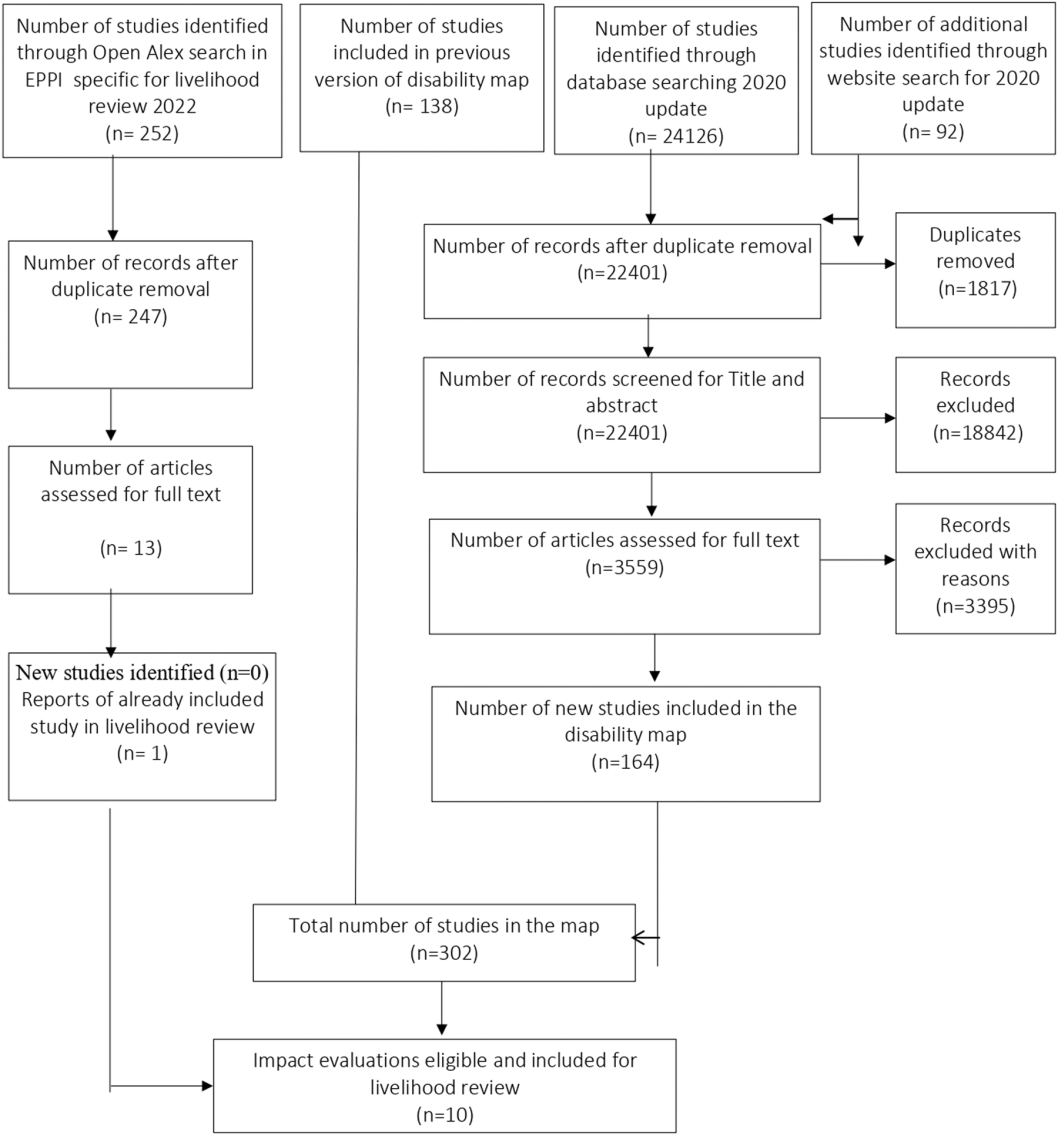
PRISMA‐evidence and gap map and livelihoods systematic review

The screening process is reported using a PRISMA flow chart (Figure 3).

The screening checklist (Supporting Information: Annex 1) included the following:

1. Intervention and outcome: Does the study include a relevant intervention AND a relevant outcome?
  a) Skills development
  b) Self‐employment
  c) Waged employment
  d) Financial services
  e) Social protection
  f) Employment in formal and informal sector
  g) Access to job market
  h) Access to financial services such as grants and loans
  i) Access to social protection programmes
  j) Participation in development of inclusive policies
2. Population: Is the study conducted with people with disabilities living in LMIC, or with the families of people with disabilities living in LMIC, or with service providers working with people with disabilities living in LMIC?
3. Study design: Is the study one in which participants are randomly assigned or quasi‐randomly assigned, or where nonrandom assignment has been done, but participants have been matched on pre‐tests and/or relevant demographic characteristics or statistical methods have been used to control for differences between groups; or where the design attempts to detect whether the intervention has had an effect significantly greater than any underlying trend over time, using observations at multiple time points before and after the intervention (interrupted time‐series design); or where participants receiving an intervention are compared with a similar group from the past who did not (i.e., a historically controlled study); or where observations are made on a group of individuals before and after an intervention, but with no control group (single‐group before‐and‐after study).

#### 4.3.2 Selection of studies

Two review authors independently determined inclusion and exclusion decisions through screening titles, abstracts, and full‐text articles of the search results using EPPI reviewer software.

#### 4.3.3 Data extraction and management

Two review authors independently extracted the necessary data from each study report. Data and information were extracted on available characteristics of participants, intervention characteristics and control conditions, research design, sample size, risk of bias and outcomes, and results. Extracted data were stored electronically. The coding sheet for this review is included as Supporting Information: Annex 2.

#### 4.3.4 Assessment of risk of bias in included studies

Table 3 presents the tool which was used to assess confidence in study findings. This tool^2^ contains six criteria:

1. Study design (Potential confounders considered): impact evaluations need either a well‐designed control group, preferably based on random assignment, or an estimation technique which controls for confounding and the associated possibility of selection bias.
2. Masking (RCTs only, also known as blinding): masking helps limit the biases which can occur if study participants, data collectors or data analysts are aware of the assignment condition of individual participants.
3. Presence of a power calculation: many studies may be underpowered, but it is difficult to assess without the inclusion in the study of a power calculation.
4. Attrition can be a major source of bias in studies, especially if these is differential attrition between the treatment and comparison group so that the two may no longer be balanced in pre‐intervention characteristics. The US Institute of Education Sciences What Works Clearing House has developed standards for acceptable levels of attrition, in aggregate and the differential, which we applied.^3^
5. Clear definition of disability: for a study to be useful the study population must be clear, which means that the type and severity of disability should be clearly defined, preferably with reference to a widely used international standard
6. Clear definition of outcome measures is needed to aid interpretation and reliability of findings and comparability with other studies. Studies should clearly state the outcomes being used with a definition and the basis on which they are measured, preferably with reference to a widely used international standard.
7. Baseline balance shows that the treatment and comparison groups are the same at baseline. Lack of balance can bias the results.

**Table 3:** Confidence in study findings assessment criteria

	Criterion	Low	Medium	High
1	Study design (Potential confounders considered)	Before and after. Naïve matching	IV, RDD, PSM, double difference	RCT, natural experiment
2	Masking (RCTs only)	No mention of masking	Masking for analysis.	Masking of data collection (where feasible). Masking for analysis.
3	Losses to follow up are presented and acceptable	Attrition not reported, OR falls well outside WWC acceptable combined levels or attrition >50%	Overall and differential attrition close to WWC combined levels	Overall and differential attrition within WWC combined levels
4	Disability/impairment measure is clearly defined and reliable	No definition	Unclear definition OR Single question item only (e.g., are you disabled)	Clear definition, e.g., Washington Group questions, detailed measure of impairment
5	Outcome measures are clearly defined and reliable	No definition	Unclear definition	Clear definition using existing measure where possible
6	Baseline balance (N.A. for before and after)	No baseline balance test (except RCT) OR reported and significant differences on more than five measures. PSM without establishing common support.	Baseline balance test, imbalance on 5 or fewer measures	RCT, RDD
	Overall confidence in study findings	Low on any item	Medium or high confidence on all items	RCT with high confidence on all items

Confidence in study findings was rated high, medium, or low, for each of the criteria, applying the standards as shown in Table 3. Overall confidence in study findings was determined to be the lowest rating achieved across the criteria—the weakest link in the chain principle.

#### 4.3.5 Measures of treatment effect

We found that it was not possible to conduct a meta‐analysis, and generate pooled results or compare effect sizes, given the diversity of designs, methodologies, and outcome measures across studies in this area, as well as poor reporting of parameters required to calculate standardised measures of effect. However, when effect sizes cannot be pooled, study‐level effects were reported in as much detail as possible.

#### 4.3.6 Unit of analysis issues

The unit of analysis of interest to the present review was individual people with disabilities, their caregivers, carers, or those working with them. If a study was included with more than two intervention arms, we included only intervention and control groups that met the eligibility criteria. Where multi‐arm studies were included, we ensured not to double‐count participants, and separately report eligible interventions and their respective outcomes.

#### 4.3.7 Criteria for determination of independent findings

Multiple publications of the same study were examined as a single study.

#### 4.3.8 Dealing with missing data

Missing data were recorded in the quality assessment tool.

#### 4.3.9 Assessment of heterogeneity

It was not possible to conduct heterogeneity analyses using standard procedures (Cochran's *Q*, *I*²) for doing so, as these depend on standardised effect sizes, which could not be calculated in the present study (as discussed under ‘Measures of treatment effect’). Indeed, give the type of synthesis of findings presented in this systematic review (narrative rather than meta‐analytic), a formal assessment of heterogeneity was not deemed appropriate, as the reasons prohibiting a meta‐analysis also prohibited the formal assessment of heterogeneity.

#### 4.3.10 Assessment of reporting biases

Assessment of reporting biases is covered under the section above ‘Quality assessment and assessment of risk of bias in included studies’.

#### 4.3.11 Data synthesis

Coding included: (1) basic study characteristics, (2) narrative summary (including annotation of any adverse effects), (3) summary of findings/results table, and (4) assessment of confidence in study findings. This coding was conducted by pairs of coders, with comparison and discussion to resolve any discrepancies which arise. There was a 93% agreement rate between coders for the study characteristics and 85% agreement rate between coders for confidence in study findings.

Data was extracted from the studies according to an extraction form which was piloted before use, and included the following sections:

1. Setting
2. Intervention category
3. Outcome category
4. Participants/target group
5. Gender of target group
6. Participants SES
7. Type of disability
8. Region
9. Country of study (specify)
10. Geographical setting of the intervention
11. Study design
12. Subject assignment
13. Duration of study

Data were summarised and findings presented in a narrative synthesis.

#### 4.3.12 Subgroup analysis and investigation of heterogeneity

We did not conduct subgroup analyses as part of a meta‐analysis given the high level of heterogeneity in reporting and effect sizes.

#### 4.3.13 Sensitivity analysis

No sensitivity analyses were conducted.

#### 4.3.14 Treatment of qualitative research

We did not include qualitative research.

## 5 RESULTS

### 5.1 Description of studies

#### 5.1.1 Results of the search

The Preferred Reporting Items for Systematic Reviews and Meta‐Analyses (PRISMA) flowchart (Figure 3) outlines the steps in the review process. The electronic databases searches for the EGM yielded 24126 potentially relevant documents for review, additional 92 studies were identified from grey literature search, reference and citation searching. The results from all three searches were combined, exported, and deduplicated using the reference management software EPPI reviewer 4 and we identified 1817 duplicates. We reviewed the titles and abstracts of the remaining 22,401 documents to determine potential relevance, excluding 18,842 due to irrelevance to the review, leaving 3559 articles for full paper review to determine inclusion in the review. Of these 3395 were excluded, and 164 new studies deemed relevant for the updated review. These 164 were pooled with the 138 studies which were identified from the previous EGM search, bringing the total count of included studies for this effectiveness map to 302. Of these 302, 9 impact evaluations were found to be eligible for inclusion in livelihood review.

As noted in the methods section, to identify any relevant articles that may have been missed during the EGM processes, we also ran the searches with search terms specific to livelihood review using Open Alex in EPPI reviewer. We identified an additional 252 studies, the results were deduplicate and we identified 247 studies that were screened for title and abstract. Only 13 studies were included for full text review. Only one study was included for data extraction, however, it was a paper from a study that was already included and hence the papers were linked (discussed below).

#### 5.1.2 Included studies

Included studies are summarised in Tables 4 and 5, and discussed in detail below.

**Table 4:** Intervention and outcome details by study

Study	Country	Design	Allocation	Sample size	Impairment types	Sample age	Intervention	Livelihoods outcomes^a^	Results	Unit of effect
Eniola and Adebiyi (2007)	Nigeria	Two‐group uncontrolled before and after (two interventions groups)	Individual random	32 (Treatment Group 1 and Treatment Group 2): Pre: *n* = 32 Post: *n* = 32	Visual	School‐aged (unspecified)	Emotional intelligence and goal setting (Individual level)	Motivation to work	A significant increase in the level of motivation postintervention compared pre‐intervention across the whole sample (*p* < 0.05). Motivation to work: Pre: 9.4 (0.52) Post: 15.9 (1.86) (mean change score 6.5; *F* = 7.98; *df* = 1,28; *p* < 0.05).	Mean change score
Grider and Wydick (2016)	Ethiopia	Posttest only with propensity score matching	Matched nonrandom	261	Physical	*M* = 35.9	Wheelchair provision	Ambition Time Spent working Time Spent begging Weekly Income Probability of having a job (Individual level)	Provision of wheelchairs led to time reallocation away from begging (1.40 fewer hours per day) *p* = 0.0004 and towards income‐generating activity (1.75 more hours per day) *p* < 0.001 and 77.5% higher income, *p* < 0.05. Level of ambition also improved (*p* = 0.01).	Mean change score; odds Ratios
Hansen et al. (2007)	Bangladesh	Uncontrolled before and after (one intervention group)	NA	109	Physical	15–50 years	An integrated community‐based rehabilitation and vocational training initiative (Indiviual level)	Reemployment	50% of the participants successfully reintegrated into paid employment, of which three quarters returned to occupations very similar to their previous ones.	Mean change score
Mauro et al. (2014)	India	Quasi‐randomised controlled trial	Cluster	2540	Convulsions, hearing and speech, intellectual, leprosy, mental, physical, and visual	Unspecified	Community‐based rehabilitation (CBR) programmes (Individual level)	Access to pensions Access to paid jobs	A positive and significant impact of the programme on access to paid jobs (by 12.3%, *p* ≤ 0.001) and access to pensions (by 29.7%, *p *≤ 0.001).	Mean change score
Biggeri et al. (2014)	India	Case‐control with propensity score matching	Cluster	1918 intervention; 414 control	Convulsions, hearing and speech, intellectual, leprosy, mental, physical, and visual	Unspecified	Community‐based rehabilitation (CBR) programmes (Individual level)	Opportunities to work Opportunities to find work Ability to work Ability to contribute economically to household	Improvements in mean score across all outcomes.	Means reported, significance tests not conducted
Nuri et al. (2012)	Bangladesh	Uncontrolled before and after (one intervention group)	NA	261	Physical, sensory	Unspecified	Vocational training programme (Individual)	Employment Family livelihood	A 60% increase in employment rate among participants and a 74% improvement in self‐reported capacity of participants to provide for their families.	Mean change score
Vilela and Leite (2017)	Brazil	Uncontrolled before and after (one intervention group)	NA	13	NA	26–58 years	Sensitization to the Inclusion of People with Disabilities in the workplace programme (Programme level)	Conceptions of disability	Statistically significant improvements on one construct (normality—the degree to which disability is seen as a dysfunctional deviation from normality) from the pre‐ and postintervention scores using the Conceptions of Disability Inventory (*p* = 0.01).	*t*‐Test
Pereira‐Guizzo et al. (2012)	Brazil	Two‐group uncontrolled before and after (two intervention groups)	Not reported	16	Physical	18–36 years	Professional Social Skills Training Programme (Individual level)	Social skills	Post‐intervention, significant improvements were already observed in Group 1 (*p* = 0.001) which increased in the first follow‐up assessment (*p* = 0.01) and was maintained in the second follow‐up assessment (*p* = 0.008). Group 2 maintained its initial scores throughout both preintervention evaluations, improving and then maintaining higher scores after the intervention (*p* < 0.001).	Mean change score
Shore and Juillerat (2012)	India, Vietnam	Uncontrolled before and after (one intervention group)	NA	519	Physical	1–100+ years	Wheelchair provision (Individual level)	Reported income Employment	The percentage of respondents in their study who reported having some employment had increased (*p* < 0.001), as had the number who reported adequate income (*p* < 0.001).	Mean change score
Zhang et al. (2017)	China	Randomised controlled trial	Individual random	162	Mental	*M* = 32.8	Integrated supported employment (ISE) (Individual level)	Employment rate Job tenure	The intervention resulted in significantly higher employment rate and longer job tenure in the ISE group (63.0%, 29.56 weeks) compared with the IPS group (50.0%, 25.47 weeks) and TVR group (33.3%, 9.91 weeks).	Mean change score; odds ratio

^a^ Outcomes related to domains other than livelihoods are not listed here.

**Table 5:** Intervention content and implementation details

Study	Intervention	Intervention description	Setting	Delivery agent	Details on delivery agent training	Details on dosage
Eniola and Adebiyi (2007)	Emotional intelligence and goal setting	Two active interventions were delivered. The first was an emotional intelligence intervention, and the second was a goal setting intervention. Both aimed to improve motivation to work. The intervention included groups sessions, where the method of instruction included lectures, discussions, demonstrations, and homework assignments.	The intervention was delivered at a local conference centre in Ibadan and Osogbo, Nigeria. Participants were recruited from a school for children with disabilities in the same areas.	The study author/s	None given	Each intervention lasted six weeks, and included two intervention sessions a week.
Grider and Wydick (2016)	Wheelchair provision	Wheelchairs were allocated by charitable organisations to individuals with physical impairments.	The wheelchairs were provided to individuals living in specific communities in Addis Ababa, Ethiopia. Study participants were recruited from the networks of nongovernmental organisations that work with people with disabilities, in these same areas.	Nongovernmental organisation/s	NA	Once provided a wheelchair, individuals had consistent access to it. Duration of access to a wheelchair at the time of the study varied among participants.
Hansen et al. (2007)	An integrated community‐based rehabilitation and vocational training initiative	A work rehabilitation programme which included prework training (physical conditioning), vocational training (including simulated work practice), workplace skills training (on issues of productivity, safety, physical tolerance and work behaviour), work placements, and follow‐up visits.	The programme was delivered through a rehabilitation centre in Dhaka, Bangladesh. Study participants were recruited via convenience sampling from communities in Dhaka.	Occupational therapists	None given	None given
Biggeri et al. (2014), Mauro et al. (2014)	Community‐based rehabilitation (CBR) programmes	A CBR programme managed by nongovernmental organisations (no additional detail given).	The CBR programme was delivered through two nongovernmental organisations operating in urban, semi‐urban, and rural areas of two districts in Karnataka state, India. Participants were recruited from the villages in which they live.	Trained community CBR workers supported by a supervisor and a project coordinator with a high involvement of people with disabilities through self‐help groups	None given	None given
Nuri et al. (2012)	Vocational training programme	A vocational training programme including individualised training plans and job placements.	The intervention was delivered by the Madhab Memorial Vocational Training Institute (MMVTI), part of the Centre for the Rehabilitation of the Paralysed in Bangladesh. Participants were individuals who had taken part in the MMVTI training programme between 1999 and 2009 and were recruited from five different districts around the area.	A multidisciplinary team of doctors, therapists, social workers, counsellors, and other professionals.	None given	None given
Vilela and Leite (2017)	Sensitisation to the Inclusion of People with Disabilities in the workplace programme	The intervention consisted of the training course ‘Sensitization to the Inclusion of People with Disabilities in the workplace’, which aimed at a critical reflection on the following issues: diversity, disability, inclusion, and employment of people with disabilities. This course was based on a continuous training programme for educators, a useful didactic and methodological approach to change conceptions and attitudes towards people with disabilities, Sensitization training course consisted of five sessions promoting critical thinking through group discussions between the instructor and the participants.	The intervention was delivered on the university campus of São Paulo State University, in São Paulo, Brazil. The intervention was delivered at the university during working hours, and the participants were released to attend the course.	The study author/s	None given	The group sessions were held once a week for 5 successive weeks (each session lasting 90 min).
Pereira‐Guizzo et al. (2012)	Professional Social Skills Training Programme	The Programme for the Development of Social Skills for the Work Environment, a professional social skills programme composed of training on civility, feedback, communication, empathy, offering help, citizenship, assertiveness, dealing with criticism, problem solving, performance in a job interview and expressing positive feelings.	The programme was delivered in, and its participants recruited from, two institutions which support people with disabilities; one linked to the Municipal Bureau of Social Welfare in one of the cities in the state of São Paulo, and one philanthropic association located in another city in the state.	The study author/s	None given	The intervention comprised 16 group sessions, carried out twice a week, lasting approximately 90 min each.
Shore and Juillerat (2012)	Wheelchair provision	Wheelchairs were allocated by nongovernmental organisations to individuals with physical impairments.	The wheelchairs were distributed by local affiliate organisations working in rural and urban communities in India and Vietnam. Participants for the study were recruited from the communities in which the wheelchairs were distributed.	Nongovernmental organisation/s	NA	Once provided a wheelchair, individuals had consistent access to it. Participants were assessed after 12 months of wheelchair use.
Zhang et al. (2017)	Integrated supported employment (ISE)	The Integrated Supported Employment programme was an evidence‐based vocational rehabilitation intervention for people with schizophrenia that integrated IPS (Individual Placement and Support) and WSST (work‐related social skills training).	Participants were recruited from Wuxi Mental Health Centre, a psychiatric hospital providing clinical services and rehabilitation interventions in Nanjing, China.	Psychiatric nurses and staff members (including an occupational therapist and nurses) of the mental health centre	No details of training, but the study authors did note that quality assurance of training delivery was assessed regularly using standardised fidelity checklists and supervision.	The WSST was 10 sessions long.

Participant characteristics and intervention setting:

List of included studies:

1. Biggeri et al. (Biggeri et al., 2014)
2. Mauro et al. (Mauro et al., 2014)
3. Eniola and Adebiyi (Eniola & Adebiyi, 2007)
4. Grider and Wydick (Grider & Wydick, 2016)
5. Hansen, Mahmud, and Bhuiyan (Hansen et al., 2007)
6. Nuri et al. (Nuri et al., 2012)
7. Vilela and Leite (Vilela & Leite, 2017)
8. Pereira‐Guizzo, Del Prette, and Del Prette (Pereira‐Guizzo et al., 2012)
9. Shore and Juillerat (Shore & Juillerat, 2012)
10. Zhang et al. (Zhang et al., 2017)

Biggeri et al. (Biggeri et al., 2014) and Mauro et al. (Mauro, 2014) reported on the same intervention, and so are discussed together in the sections below, except where we note otherwise. As such, although there are 10 papers/studies included in this review, there are only 9 interventions reported on.

##### 5.1.2.1 Target group

Only one of the nine interventions targeted children with disabilities alone (Eniola & Adebiyi, 2007), and only two included a mix of age groups (children and adults with disabilities) (Hansen et al., 2007; Shore & Juillerat, 2012). Most of the interventions targeted adults with disabilities only (Grider & Wydick, 2016; Pereira‐Guizzo et al., 2012; Zhang et al., 2017). One study included service providers (Vilela & Leite, 2017) rather than people with disabilities themselves. In this study, thirteen academic staff and administrative staff members in three different colleges of a public university in Brazil participated in an intervention on sensitization to the inclusion of people with disabilities in the workplace (Vilela & Leite, 2017). Three studies did not report participant age (Biggeri et al., 2014; Mauro et al., 2014; Nuri et al., 2012). All interventions targeted both men and women. Only two studies specifically noted participant socioeconomic status (Pereira‐Guizzo et al., 2012; Shore & Juillerat, 2012).

##### 5.1.2.2 Impairment groups

Seven of the included interventions targeted individuals with a single type of impairment, and only one intervention catered to people with a range of impairments (Biggeri et al., 2014; Mauro, 2014). There was also one programme for people without disabilities who were service providers (Vilela & Leite, 2017). Most single impairment group interventions targeted people with physical impairments alone (Grider & Wydick, 2016; Hansen et al., 2007; Nuri et al., 2012; Pereira‐Guizzo et al., 2012; Shore & Juillerat, 2012). The remaining single impairment group interventions were for people with psychosocial (Zhang et al., 2017) and visual impairments (Eniola & Adebiyi, 2007), respectively.

##### 5.1.2.3 Region

Ten countries were represented across the studies, some of which were multi‐country. Two studies were conducted in the East Asia and Pacific region (Shore & Juillerat, 2012; Zhang et al., 2017), two in Latin America and the Caribbean (Pereira‐Guizzo et al., 2012; Vilela & Leite, 2017), two in Sub‐Saharan Africa (Eniola & Adebiyi, 2007; Grider & Wydick, 2016), and four in South Asia (Biggeri et al., 2014; Hansen et al., 2007; Mauro, 2014; Nuri et al., 2012; Shore & Juillerat, 2012). There were no studies from Europe and Central Asia or the Middle East and North African region. The South Asian countries represented were Bangladesh (Hansen et al., 2007; Nuri et al., 2012) and India (Biggeri et al., 2014; Mauro, 2014; Shore & Juillerat, 2012), the African countries were Nigeria (Eniola & Adebiyi, 2007) and Ethiopia (Grider & Wydick, 2016), while Brazil (Pereira‐Guizzo et al., 2012; Vilela & Leite, 2017) represented South America, and China and Vietnam (Shore & Juillerat, 2012; Zhang et al., 2017), East Asia and the Pacific.

##### 5.1.2.4 Geographical setting of the intervention

The vast majority of the interventions were delivered in urban areas (Eniola & Adebiyi, 2007; Grider & Wydick, 2016; Hansen et al., 2007; Pereira‐Guizzo et al., 2012; Zhang et al., 2017), with none explicitly reporting targeting rural populations alone, and three noting that they reached a mix of rural and urban participants (Biggeri et al., 2014; Mauro et al., 2014; Shore & Juillerat, 2012). In two of the studies, the setting was not reported (Nuri et al., 2012; Vilela & Leite, 2017). In one of these, it was not clear from the districts in which the intervention took place, whether rural or urban settings were covered (Nuri et al., 2012), while in the other, a manual internet search of the institution where the intervention took place revealed that the participants were also likely urban‐based (Vilela & Leite, 2017).

##### 5.1.2.5 Setting and level

Four interventions were community‐based (including community‐based vocational training programmes) (Biggeri et al., 2014; Hansen et al., 2007; Mauro et al., 2014; Shore & Juillerat, 2012), while six were institution‐based (including school‐based social and life skills training) (Eniola & Adebiyi, 2007; Grider & Wydick, 2016; Nuri et al., 2012; Pereira‐Guizzo et al., 2012; Vilela & Leite, 2017; Zhang et al., 2017). Eight of the interventions focussed on the individual level, one at the programme level (Vilela & Leite, 2017) and none at the level of the system.

Table 4 presents intervention and outcome details of included studies.

#### 5.1.3 Study characteristics

##### 5.1.3.1 Study design

The research designs of the studies included one randomised controlled trial (Zhang et al., 2017), one quasi‐randomised controlled trial (a randomised, posttest only study using propensity score matching (PSM) (Mauro et al., 2014), one case‐control study with PSM (Biggeri et al., 2014), four uncontrolled before and after studies (Eniola & Adebiyi, 2007; Pereira‐Guizzo et al., 2012; Shore & Juillerat, 2012; Vilela & Leite, 2017), and three posttest only studies (Grider & Wydick, 2016; Hansen et al., 2007; Nuri et al., 2012), one where PSM was also used, and two with implied baselines as being unemployed was a prerequisite for being enroled into the programmes.

##### 5.1.3.2 Subject assignment

In two studies, subject assignment was individual random (Eniola & Adebiyi, 2007; Zhang et al., 2017), and in one study whole group random (Mauro et al., 2014). In one study, there was matched, nonrandom (Grider & Wydick, 2016) subject allocation. However, in most cases, nonmatched and nonrandom subject assignment was used in determining intervention participation and/or participation in the associated impact evaluation (Hansen et al., 2007; Nuri et al., 2012; Shore & Juillerat, 2012). Allocation was not reported in two studies (Pereira‐Guizzo et al., 2012; Vilela & Leite, 2017), and no allocation was used in the case‐control with PSM (Biggeri et al., 2014).

##### 5.1.3.3 Intervention characteristics

We used the above‐detailed table when extracting data on the included studies, loosely based on the interventions associated with the livelihoods pilar of the CBR matrix. Many of the interventions were multicomponent, and so fell into several categories. In terms of skills development, three interventions aimed to improve training opportunities for employment such as vocational training (Hansen et al., 2007; Nuri et al., 2012; Zhang et al., 2017), and three provided social, life and communications skills training (Eniola & Adebiyi, 2007; Pereira‐Guizzo et al., 2012; Zhang et al., 2017). In the area of improving access to waged employment, two programmes aimed to facilitate physical access to the workplace (Grider & Wydick, 2016; Shore & Juillerat, 2012), and one included job placement as an intervention component (Nuri et al., 2012). Two vocational training programmes were included, and both targeted formal employment as well as equipping people with disabilities to sell goods and services (Hansen et al., 2007; Nuri et al., 2012). One programme, conducted with individuals without disabilities, aimed to remove social and attitudinal barriers to access for people with disabilities (Vilela & Leite, 2017). Finally, four interventions aimed to improve livelihoods by improving access to rehabilitation (Biggeri et al., 2014; Hansen et al., 2007; Mauro et al., 2014), or assistive technology (Grider & Wydick, 2016; Shore & Juillerat, 2012). Several categories of possible intervention, including financial services, social protection and policy change were absent from the included studies. Overall, eight of the interventions focussed on the individual‐level, one at the programme‐level (Vilela & Leite, 2017) and none at the level of the system. Additional detail on intervention setting, delivery, and implementation, as well as dosage, are presented in Table 5.

##### 5.1.3.4 Outcome characteristics

The outcomes of the included interventions were mapped in a similar manner to the interventions (i.e., against a table loosely based on the CBR matrix). The main outcome of two programmes were ‘acquisition of skills for the workplace’, specifically social and communication skills needed for work (Pereira‐Guizzo et al., 2012). In the domain ‘access to the job market’, two studies examined outcomes to do with the capacity of people with disabilities to engage in job searching (Biggeri et al., 2014; Eniola & Adebiyi, 2007), and three physical and social barriers to employment (Grider & Wydick, 2016; Shore & Juillerat, 2012; Vilela & Leite, 2017). Most outcomes fell into the category of ‘employment in formal and informal sector’, with six studies examining entrepreneurship and informal sector participation as well as waged employment and formal sector participation (Biggeri et al., 2014; Grider & Wydick, 2016; Mauro et al., 2014; Nuri et al., 2012; Shore & Juillerat, 2012; Zhang et al., 2017). Four interventions used outcomes related to ‘income and earnings from work’ (Biggeri et al., 2014; Grider & Wydick, 2016; Hansen et al., 2007; Mauro et al., 2014). Finally, one study used the outcome of access to formal and informal social protection (Mauro et al., 2014). No studies reported on outcomes related to the development of inclusive policies, or access to financial services such as grants and loans. Table 6 presents a summary of the findings of this review, by outcome of interest.

**Table 6:** Summary of findings by outcome

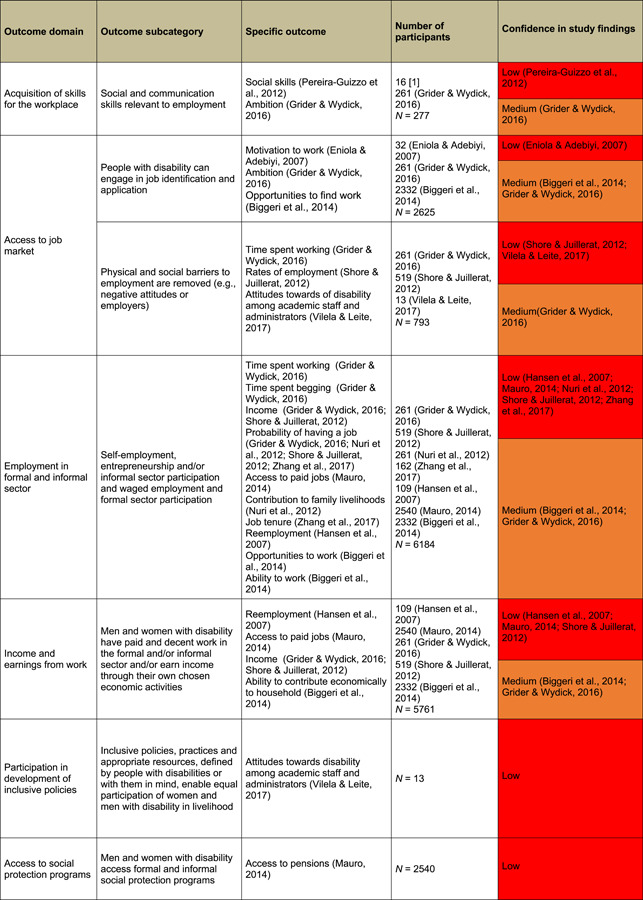				

#### 5.1.4 Excluded studies

Exclusions are recorded in the PRISMA diagram above. Excluded studies with the associated reason for exclusion are presented in Annex A. Common reasons for exclusion included that the study was not an impact evaluation, presented a protocol for which there were no associated results, focused on an ineligible population, had a livelihoods intervention but no livelihoods outcomes, presented only qualitative data, and—in one case—otherwise relevant findings were not disaggregated for people with disabilities.

### 5.2 Risk of bias in included studies

Our confidence in the overall findings is low to medium on the basis of our appraisal of the studies. Two studies (Biggeri et al., 2014; Grider & Wydick, 2016) scored medium using our assessment tool, with the remaining eight scoring low on one or more item (Eniola & Adebiyi, 2007; Hansen et al., 2007; Mauro et al., 2014; Nuri et al., 2012; Pereira‐Guizzo et al., 2012; Shore & Juillerat, 2012; Vilela & Leite, 2017; Zhang et al., 2017). There is diversity within low ratings as we employed the weakest link in the chain principle to assess confidence in study findings (Table 7). However, the findings of a study receiving a low rating on a single item (e.g., Zhang et al., 2017 for reporting of attrition) should not be treated in the same manner as those derived from a study rating low on multiple items. The latter approach allows for valuable learnings not to be overlooked due to an overall ‘low’ confidence in study findings score, in studies which had many areas of strength.

**Table 7:** Confidence in study findings appraisal

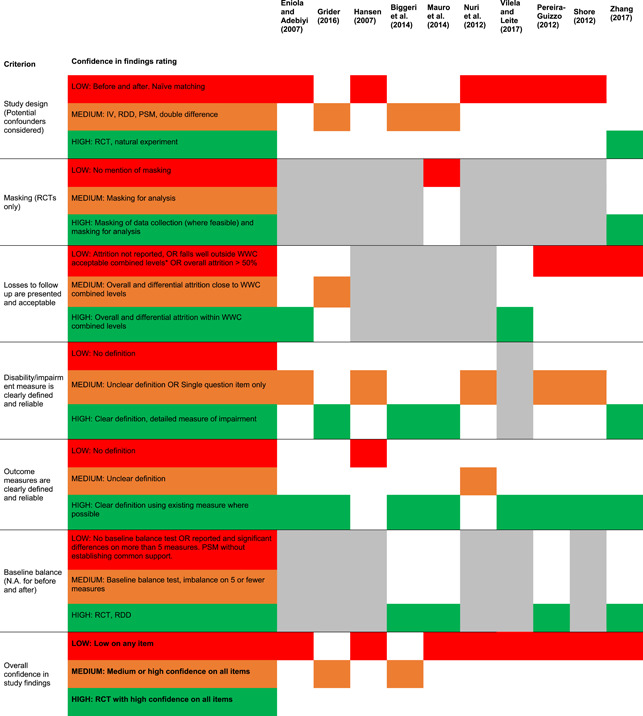

#### 5.2.1 Appraisal by criterion

##### 5.2.1.1 Study design

Most studies were rated ‘low’ on study design (Eniola & Adebiyi, 2007; Hansen et al., 2007; Nuri et al., 2012; Pereira‐Guizzo et al., 2012; Shore & Juillerat, 2012; Vilela & Leite, 2017), as many used before and after designs, often without a control group. Furthermore, two of the included before and after studies, by Nuri et al. (Nuri et al., 2012) and Hansen et al. (Hansen et al., 2007), only had implied baselines and data was only collected after the intervention was administered. However, entry into the interventions in these cases required meeting of certain baseline criteria (e.g., unemployment), and so both were treated as before and after studies. Three studies were rated medium (Biggeri et al., 2014; Grider & Wydick, 2016; Hansen et al., 2007) in our assessment of confidence in study findings based on design, as they employed PSM (in a quasi‐randomised controlled trial and in a case‐control study) or double difference techniques to mimic the conditions of a more rigorous design. There was only a single randomised controlled trial (Zhang et al., 2017), and so only one ‘high’ rated study on design.

##### 5.2.1.2 Masking

For most studies, masking was not an applicable measure of confidence in study findings, as few were RCTs. However, for the single randomised controlled trial, masking was implemented and reported (Zhang et al., 2017). For the quasi‐randomised controlled trial, masking was not reported, and so a rating of ‘low’ was recorded (Mauro et al., 2014).

##### 5.2.1.3 Losses to follow up presented and acceptable

For reporting and acceptability of loss to follow up, three studies received ratings of ‘low’ (Pereira‐Guizzo et al., 2012; Shore & Juillerat, 2012; Zhang et al., 2017), one of ‘medium’ (Grider & Wydick, 2016), and two of ‘high’ (Eniola & Adebiyi, 2007; Vilela & Leite, 2017). The low ratings were because attrition went unreported, even in otherwise well‐reported studies. The single randomised controlled trial, for instance, which received high scores on all other indices of confidence in study findings, failed to report attrition and was therefore downgraded to an overall assessment of ‘low’, despite strengths in other areas. For Hansen et al. (Hansen et al., 2007), Biggeri et al. (Biggeri et al., 2014), Mauro et al. (Mauro, 2014) and Nuri et al. (Nuri et al., 2012), loss to follow up was not applicable as the data were collected at one time point only.

##### 5.2.1.4 Disability/impairment measure definition and reliability

One of the areas which received relatively good ratings across studies was the use of disability measures or definitions which were consistently clear and reliable. No studies received a rating of ‘low’. Most studies received ratings of ‘medium’ (Eniola & Adebiyi, 2007; Hansen et al., 2007; Nuri et al., 2012; Pereira‐Guizzo et al., 2012; Shore & Juillerat, 2012). In these papers, impairment type was mentioned, and associated diagnoses listed (but how these were arrived at was not reported), or single items were used to determine disability status (i.e., ‘do you have a spinal cord injury’), but more rigorous disability assessments or clearer operational definitions were omitted. In four studies, rigorous and replicable criteria were used, and high ratings were given (Biggeri et al., 2014; Grider & Wydick, 2016; Mauro et al., 2014; Zhang et al., 2017). In Zhang et al.'s (Zhang et al., 2017) study of individuals with psychosocial disabilities (schizophrenia), The Brief Psychiatric Rating Scale was used to assess the participants' psychiatric status. In the study of (Grider & Wydick, 2016), every individual in the sample had been seen by a physician and had been deemed physically in need of a wheelchair. Finally, (Mauro et al., 2014) defined disability following the instructions of the World Health Organization's Community‐based Rehabilitation Manual. Vilela and Leite (2017) targeted participants who were people without disability working with individuals with disabilities, rather than people with disabilities themselves, and as such this publication was not assessed for this criterion. It is worth noting that reliability and validity of specific outcome measures used was scarcely discussed in the publications, and so it was not possible to systematically extract information on these indices.

##### 5.2.1.5 Outcome measure definition and reliability

Outcome measures were largely well‐defined, perhaps reflective of the tendency of the studies to be outcome‐driven interventions, and so primarily concerned with operationalizing and then acting upon, a particular dimension of livelihoods. All but two studies (Hansen et al., 2007; Nuri et al., 2012) received high ratings on this item.

##### 5.2.1.6 Baseline balance

Baseline balance was only relevant for four of the studies; the randomised controlled trial (Zhang et al., 2017), the two studies using PSM (Biggeri et al., 2014; Mauro et al., 2014), and a two group before and after study (Pereira‐Guizzo et al., 2012). The randomised controlled trial and the studies using PSM reported acceptable baseline balance and were coded as high on this item. However, the two group before and after study by Pereira‐Guizzo et al. (2012) was scored as medium on baseline balance as there was evidence of baseline balance but the sample size per group was very small (*n* = 8) for each group.

#### 5.2.2 Appraisal by study

Nuri et al. (Nuri et al., 2012) received a ‘low’ rating on study design, as they only collected data at a single time point, postintervention. However, admission to the intervention was predicated on lack of employment, and the outcomes measured at the assessment time point included employment gained, and so the study was implicitly a before and after design, and therefore included in this review. A ‘medium’ rating was given to the definition of disability used, as the authors did list conditions (impairments resulting from a variety of physiological conditions, cosmetic disfigurements, spinal cord dysfunctions, musculoskeletal losses, sensory impairments, and various types of chronic diseases) which merited inclusion but did not report how eligibility was determined. Outcome measures were not clearly defined, but a rating of medium was given as percentages of certain binary measures (secured employment/did not secure employment) were reported. Masking, attrition, and baseline balance were not relevant criteria, given the design, and so not scored. An overall score of ‘low’ was assigned to confidence in these study findings.

The study of Eniola and Adebiyi (2007) received a design rating of low, as the study employed a before and after experimental group design. The study received a rating of high for its reporting of losses to follow up, as before and after scores were reported for the total sample, indicating no attrition. The disability/impairment measure rating for this study was medium as the authors note that they enroled visually impaired students from the School for Handicapped Children in Ibadan and Osogbo, Nigeria, but did not record what degree of vision impairment was included, or whether there was variation in the group. The study received a high rating for its definition and reliability of outcome measures, as the ‘Work Value Inventory’ was an existing tool developed by Salami (2000). Masking and baseline testing were not relevant given the study design, and so not scored. The overall confidence in study findings score for this study was low.

The study of Grider and Wydick (2016) received a study design score of medium, as the authors reporting using covariate matching, seeming unrelated regressions (SUR), and a series of robustness checks for endogeneity within their two group (intervention and control) design. Losses to follow up was rated as medium, given that attrition was not explicitly reported, and for some outcomes observations were noted to be for the full sample (*n* = 261), while for other outcomes one observation was missing (*n* = 260). This could be read to imply the loss of one individual to follow up for some items, which—had it been reported—would have rendered a rating of high on this item. However, given the failure to report attrition, the score was downgraded to a medium. The definition of disability/impairment was rated as high, as the authors noted that the sample comprised individuals with a range of physical impairments, and described the range of aetiologies and impairment types included in the broader group (polio, infections, work accidents, war victims, muscular dystrophy and leprosy identified by wheelchair recipient lists and waitlists). The outcomes of interest were also score high, as they were clearly defined: more hours per day for work, fewer hours per day for street begging, and percentage increase in income. Masking and baseline testing were not relevant given the study design, and so not scored. The overall finding regarding confidence in these study findings was medium, driven by item ratings for robustness of the study design, and reporting of attrition.

Hansen et al. (Hansen et al., 2007) received a rating of ‘low’ on study design as the study employed an uncontrolled before and after design. A rating of ‘medium’ was given for the authors' definition of disability/impairment, as they did not report using a standardised measure, but did note that all participants had spinal cord injuries. Definition and reliability of outcome measures was rated ‘low’ in this study, as definitions of key outcomes were lacking. Masking, loss to follow up, and baseline testing were not relevant given the study design, and so not scored. Overall confidence in study findings was rated ‘low‘ for this study.

Biggeri et al. (Biggeri et al., 2014) and Mauro et al. (Mauro, 2014) reported on the same intervention, but using slightly different designs. As such, both similar ratings for many criteria. A rating of ‘medium’ was given to both on study design as Biggeri et al. (Biggeri et al., 2014) employed a case‐control design with PSM, and Mauro et al. conducted a quasi‐randomised controlled trial design with PSM, to evaluate the intervention. The anomaly between the two studies has to do with reporting of masking. Because Biggeri et al. (Biggeri et al., 2014) framed their study as a case control, they were not assessed on this criterion. However, Mauro et al. (Mauro, 2014) called their evaluation a quasi‐randomised trial, and so they were expected to have reported on masking, but did not and so a ‘low’ rating was given to the latter for the masking criterion. The disability/impairment measure used in both was clearly defined and reliable, and so a rating of ‘high’ was recorded for this criterion. The same applied to the outcome measures used, which were clearly defined and reliable. PSM was used in both studies to build treatment/case and control groups with balanced pretreatment covariates, and so a rating of high was given to this study on the balance criterion. An overall of ‘medium’ was given to Biggeri et al. (Biggeri et al., 2014). However, a ‘low’ rating was recorded for the Mauro et al. (Mauro, 2014) study, given deficits in reporting of masking and attrition.

Vilela and Leite (Vilela & Leite, 2017) received a rating of ‘low’ on study design for conducting an uncontrolled before and after study. However, losses to follow up were presented and acceptable (participant scores were recorded for all items at both time points). Outcome measures were clearly defined and reliable (the Conceptions of Disability Inventory). The target participants in this intervention were people without disability, and so the definition of disability criterion was not applicable, and neither were masking, nor baseline balance given the design. The overall confidence in study findings was rated ‘low’.

In the study of Pereira‐Guizzo et al. (2012), a rating of low was given for study design, as although they employed a two group before and after design, they did not specify how allocation to the groups had occurred. Losses to follow up were not reported and so a rating of ‘low’ was given. A ‘medium’ rating was given for the disability/impairment measure used, as physical impairment was stated and a list of conditions was given, but it was not clear how eligibility was established. Outcome measures were clearly defined and reliable, as the authors reported using the Professional Social Skills Observation System and the Social Skills Inventory, and as such the study was rated high on this criterion. Finally, baseline balance was evidenced, but the sample sizes were very small for each group (*n* = 8) and so a rating of medium was given, rather than one of high, as would have been the case had the sample sizes been larger. Masking was not relevant for this study and so not rated. Overall, a rating of ‘low’ was recorded.

Shore and Juillerat (2012) employed an uncontrolled before and after design, thus receiving a ‘low’ rating for study design. Losses to follow up were not reported, nor was a clear and reliable measure of disability reported, leading to two additional ‘low’ scores. However, the outcome measures were clearly defined and reliable, and so a ‘high’ rating was recorded on this criterion. Neither masking nor baseline balance were relevant given study design. Overall, a low rating for confidence in study findings was allocated to this study.

Finally, Zhang et al. (Zhang et al., 2017) was the only study to receive a high rating on study design, being the only randomised controlled trial included in the review. Zhang et al. (Zhang et al., 2017) received high ratings across all indices of our tool, bar one. Unfortunately, losses to follow up were not reported, and so a ‘low’ rating was given on this criterion. This study is exemplary of the one weakness of the weakest‐link‐in‐the‐chain principle in administering a confidence in study findings appraisal tool, as an otherwise well‐designed and well‐reported study is assigned a low rating overall, based on a single failure to report.

### 5.3 Effects of interventions

All the included studies reported positive impacts on livelihoods outcomes. However, outcomes varied substantially by study, as did the methods used to establish intervention impact, and the quality and reporting of findings.

Vilela and Leite (Vilela & Leite, 2017) reported that a critical‐reflexive intervention for people without disabilities resulted in more positive social attitudes of employees and administrative staff towards the participation of people with disabilities in the workplace (as measured using the Conceptions of Disability Inventory). However, statistically significant improvements were only reported for one construct (normality) from the pre‐ and postintervention scores on the Inventory (*p* = 0.01).

Nuri et al. (Nuri et al., 2012) reported that a vocational training programme in Bangladesh led to a 60% increase in employment rate among participants and a 74% improvement in self‐reported capacity of participants to provide for their families. Statistical significance of these findings was not evaluated by the researchers.

Shore et al. (Shore & Juillerat, 2012) reported that, following 12 months of using a wheelchair, the percentage of respondents in their study who reported having some employment had increased (*p* < 0.001), as had the percentage who reported adequate income (*p* < 0.001). In another evaluation of the impact of wheelchair allocation, Grider and Wydick et al. (Grider & Wydick, 2016) reported that people with disabilities given access to a wheelchair allocated 1.75 more hours per day to work (*p* < 0.001), 1.40 fewer hours per day to street begging (*p* = 0.0004), and realised a 77.5% increase in income (*p* = 0.0001), all of which were statistically significant.

Students in Eniola and Adebiyi's (Eniola & Adebiyi, 2007) intervention for youth with visual impairments showed a significant increase in the level of motivation post‐intervention compared preintervention across the whole sample (*p* < 0.05).

In Hansen et al.'s (Hansen et al., 2007) evaluation of a vocational training programme for people with spinal cord injuries, an estimated 50% of the participants successfully reintegrated into paid employment, of which three quarters returned to occupations very similar to their previous ones. The statistical significance of these findings was not established by the researchers.

The quasi‐randomised trial evaluation of a CBR programme in India (Mauro et al., 2014) showed that a positive and significant impact of the programme on access to paid jobs (by 12.3%, *p* ≤ 0.001) and access to pensions (by 29.7%, *p* ≤ 0.001). A case‐control evaluation of the same programme, using PSM, indicted higher rates, among cases, of opportunities as your peers to find a job, ability to work, and ability to contribute economically to their household, and lower rates of difficulties finding a job, difficulties working, and difficulties contributing economically to their household.

Pereira‐Guizzo et al.'s (Pereira‐Guizzo et al., 2012) professional social skills training programme for unemployed people with physical impairments led to statistically significant improvements in work‐relevant social skills [Their study employed two groups, each engaged in social skills interventions. Post‐intervention, significant improvements were observed in both Groups 1 (*p* = 0.008) and 2^4^ over time (*p* < 0.001)].

Finally, Zhang et al. (Zhang et al., 2017) reported that an integrated supported employment (ISE) intervention, when compared with individual placement and support (IPS) and traditional vocational rehabilitation (TVR) for people with schizophrenia, resulted in a significantly higher employment rate (*p* = 0.002) and longer job tenure (*p* = 0.002) in the ISE group compared with the IPS group and TVR group.

#### 5.3.1 Synthesis of results

A quantitative synthesis was not undertaken.

## 6 DISCUSSION

### 6.1 Summary of main results

We identified, coded, evaluated, analysed, and narratively summarised the findings from 10 studies that evaluated 9 interventions to improve the livelihoods of people with disabilities in LMIC. These studies served as the data for this review and are reported according to the interventions and outcomes identified across all studies. Due to the heterogeneity of outcomes and the low level of confidence in study findings, a meta‐analysis was not deemed appropriate to this review, and so findings were presented narratively.

These findings are discussed, broadly, according to participants and programmes.

Children, older people, and service providers were underrepresented in the studies included in this review. While it is perhaps understandable that children were not often targeted for employment‐related interventions, the omission of programmes targeting older persons with disabilities (>65 years) is concerning. Moreover, both children and older people with disabilities are vulnerable to poverty (Cullinan et al., 2013; Kwan & Walsh, 2018), and so may benefit from inclusion in social protection programmes. It also appears that programmes targeting very low‐income participants were lacking, although this was possibly a function of poor reporting. Given the relationship between disability and poverty (Braithwaite & Mont, 2009; Groce et al., 2011; Mitra, 2018; Mitra et al., 2011, 2013; Trani & Loeb, 2012), it is surprising to see that few of the included studies reported the socioeconomic status of the participants.

The studies overrepresented people with physical impairments (five out of nine interventions only included people with physical impairments). This focus is possibly due to the perception that they are a relatively easy group for delivery of programmes and conduct of research, as there is no need to overcome communication or cognitive difficulties. However, there are many opportunities for meaningful intervention with people with other impairment types. Moreover, people with intellectual impairments may experience the greatest barriers to employment and livelihoods and the greatest socioeconomic disadvantage of all impairment groups (Gouvier et al., 2003; Kavanagh et al., 2015). Indeed, a recent report by Mitra and Yap (Mitra & Yap, 2021) found that people with mobility, cognitive and self‐care difficulties had the lowest employment rates. As such, more programming targeting individuals with cognitive and self‐care difficulties may be warranted.

Two regions were not represented in our review—‘Europe and Central Asia’ and ‘Middle East and North Africa’. This gap may be an attributable to the language of reporting, as our search only covered literature in English, while many of these areas are not English‐language dominant. Further, as this review only included studies conducted in LMIC, regions with a high proportion of HIC (such as Europe) may be underrepresented simply because their constituent nations are not eligible. However, it may also be the case that programming for people with disabilities' livelihoods in these regions is comparatively lacking. Future reviews may benefit from focusing on the non‐English language literature to examine this question.

It is also worth commenting on the setting of interventions. Most of the programmes were delivered through institutions such as schools, places of work, or higher education centres, and almost all the programmes were delivered in urban areas. Programmes delivered in the community, virtually, or in rural areas, were largely missing from the included papers. Considering the drive for interventions for people with disabilities to be delivered near where they reside (a core tenet of CBR), this is surprising, and indicates an area in need of improvement. Particularly, the opportunities which might be provided by online interventions to bridge gaps in access between low‐ and high‐infrastructure communities, seem to be unexplored.

Finally, regarding intervention content and outcome areas addressed, most of the included programmes aimed to improve the livelihoods of people with disabilities through removing physical barriers to work, improving willingness to seek employment and confidence to work, and equipping people with disabilities with skills with which to earn a living. Vocational training, attitude change, and wheelchair provision interventions were most common, and outcomes related to engagement in labour, and ‘soft' (i.e., instrument‐based) measures of proxies for employability (like social skills and willingness to work). This left important intervention gaps. There were no interventions which sought to improve access to or utilisation of financial services and only one which aimed to improve access to pensions. There were also no programmes aimed at improving policy involvement and provision for people with disabilities. Social protection interventions were most glaringly absent, given the importance of this intervention for people with disabilities and the high profile which these types of interventions hold in disability discourse (Banks, Mearkle, et al., 2017; Palmer, 2013). The interventions were almost all aimed at the individual level, only one at programme level and none at system level. There was therefore implicitly a focus on ‘fixing' people with disabilities rather than addressing broader barriers and facilitators.

Further, while several of the included studies reported on ‘hard' outcomes, such as increases in rates of employment or higher income, there were no studies which examined people with disabilities' access to financial services such as grants and loans. Given the lack of interventions in this area, there were also no outcomes related to the development of inclusive policies.

A final critique of the outcomes and methodologies employed, pertains to the relative absence of information on the local validity and reliability of measures. It would make sense for future work to clearly establish the validity and reliability of measures used, to strengthen the rigour of research and build confidence in conclusions reached.

Nonetheless, and despite the methodological and programmatic limitations of the studies comprising this review, all reported interventions noted positive impacts on important aspects of livelihoods including income, employment, social skills, willingness to work, and time spent working. This pattern seems to suggest that it may be possible for a variety of programming approaches to improve outcomes related to the livelihoods of people with disabilities. It is imperative that future research evaluate these approaches with more rigorous study designs, to develop a firmer evidence base to inform intervention at scale.

### 6.2 Overall completeness and applicability of evidence

The evidence presented here highlights examples of potentially effective interventions to improve the livelihoods of people with disabilities in LMIC. However, these interventions need to be evaluated using rigorous study designs before firm conclusions about their effectiveness can be drawn.

### 6.3 Quality of the evidence

The quality of the included studies is generally low, as assessed by the confidence in study findings tool. Most study designs employed were unable to consider many potential confounders. Losses to follow up and other important dimensions of study rigour were either poorly recorded or poorly reported. Although some studies did attempt to undertake robust analyses on quantitative outcomes, the ability to make definitive judgements about programme effect was undermined by the absence of controlled trials. Given our assessment of confidence in study findings, it is difficult to draw definitive conclusions from the papers included in this review.

### 6.4 Potential biases in the review process

Potential bias may be introduced about the lack of grey literature included in the review, as well as the absence of non‐English literature. The first limitation—the lack of grey literature—means that we are less likely to have identified interventions which were found to have null results. Studies finding no significant effects of interventions are more likely to go unpublished, and so by not looking beyond the published peer‐reviewed literature, we may have biased our review to including significant, positive findings. The absence of literature published in languages other than English may produce biased estimates of the scope of the literature, as Chinese, Arabic, Francophone, Hispanophone, and Lusophone LMIC (such as those in South America and West Africa) may be missed if study results are not translated or published in English.

Bias could also have been introduced were the research team to have had different ideas about relevant interventions and outcomes, or understandings of disability programming. To address this potential source of bias, all full text reviews and coding decisions were made by at least two researchers on the team coming to consensus on the decision of whether an article should be included, and how relevant information should be extracted.

### 6.5 Agreements and disagreements with other studies or reviews

Our review contributes to the body of literature concerning the livelihood component of the CBR matrix, as a previous systematic review (Iemmi et al., 2015) focussed on CBR‐specific interventions for people with disabilities did not find any studies which specifically addressed livelihoods (although one included study had livelihoods elements as minor components). However, our review had in common with that of this earlier one by Iemmi et al. (Iemmi et al., 2015), a finding of heterogeneity of interventions and scarcity of good‐quality evidence. A similar dearth of evidence, variety of studies, and low quality of evidence was found by Tripney et al. (Tripney et al., 2015) in their review of interventions to improve the labour market situation of adults with physical and/or sensory disabilities in LMIC. These authors also found that the majority of studies were conducted in a limited range of LMIC (in Asia, Africa and Latin America), and that most programmes were focused on persons with physical impairments (Tripney et al., 2015). This review also found a preponderance of single‐group pre‐test/posttest designs.

## 7 AUTHORS' CONCLUSIONS

### 7.1 Implications for practice and policy

- Despite the methodological and programmatic limitations of the studies comprising this review, all reported interventions noted positive impacts on important aspects of livelihoods, which suggests that it may be possible for a variety of programming approaches to improve outcomes related to the livelihoods of people with disabilities.
- Research evaluating programmes for people with disabilities other than physical impairments are needed, and not only programmes focussed on the individual level
- Programmes should integrate impact evaluations into their practice to improve the evidence base.

### 7.2 Implications for research

- There is not a great deal of evidence on livelihoods interventions for people with disabilities in LMIC and so more studies are needed.
- Researchers should work with OPDs and NGOs to identify priority interventions to evaluate.
- Social protection programmes, particularly, need to be rigorously evaluated and outcomes reported for subgroups of beneficiaries including people with disabilities.
- Generally, methodological details are reported poorly, making it difficult to judge inclusion, and assess risk of bias. Consistent use of outcome measures and clear reporting (e.g., standard deviations and sample sizes for treatment and control groups) would help support their inclusion in systematic reviews
- Future research could usefully evaluate those programmatic elements associated with livelihoods outcomes to distil the core components of programming which are responsible for change in key livelihoods outcomes.
- Future research could also focus on livelihoods outcomes not examined in the literature yet (interventions—financial services, social protection and policy change were absent from the included interventions; outcomes—the development of inclusive policies, or access to financial services such as grants and loans), in particular those targeted at the programme or system level, rather than the individual level.
- Overall, there is a need for more and better‐quality data to inform policy and programming, including data from people with a broader range of impairment types and from different parts of the world. Central to this project will be the collection, by countries, of disability‐disaggregated data through national information management systems. Such data could form the platform from which livelihoods programming could then be developed, saving researchers the time‐ and resource‐intensive tasks of conducting formative work, and allowing them to develop programming which responds to the needs of people with disabilities.

## CONTRIBUTIONS OF AUTHORS

- Content: XH, HK, MB
- Systematic review methods: AS, HW
- Statistical analysis: AS
- Information retrieval: AS, XH

## DECLARATIONS OF INTEREST

The authors have no interests to declare.

## DIFFERENCES BETWEEN PROTOCOL AND REVIEW

There are no differences between the protocol and the review, aside from the addition of one intervention category (equipping people with disabilities to sell goods and services) which was deemed important for the coding sheet to capture relevant data from all studies.

## SOURCES OF SUPPORT

**Internal sources**

• No sources of support provided

**External sources**

This systematic review is supported by the Commonwealth and Development Office (FCDO) under its support for the Centre for Excellence for Development Impact and Learning (CEDIL) and the Programme for Evidence to iNform Disability Action (PENDA).

EXCLUDED STUDIES

## Supporting information

Supporting information.

## Footnotes

1 Recent examples include the Law on the Rights of Persons with Disabilities adopted in India in 2016 and Indonesia Law no. 8/2016 on Persons with Disabilities.

2 Thanks also to Hugh Waddington (3ie and Campbell IDCG) for suggestions used in developing the tool.

3 See Table 1 https://homvee.acf.hhs.gov/HomVEE-Attrition-White_Paper-7-2015.pdf.

4 On the Total Score (TS) of the SSI‐Del‐Prette, Group 1 showed statistically significant improvements at time 2 (*U* = 0.10; *z* = −3.4; *p* = 0.001) time 3 (*U* = 8.5; *z* = −2.5; *p* = 0.014), and time 4 (*U* = 3.5; *z* = −2.7; *p* = 0.008). Group 2 also improved and then maintained higher scores after the intervention (*χ* ^2^ = 20.700; *p* < 0.001).

## Annex A

### Excluded studies

**Table:**

Study	Summary	Reason for exclusion
Van Niekerk, L., Coetzee, Z., Engelbrecht, M., Landman, S., Motimele, M., Terreblanche, S., & Hajwani, Z. (2011). Supported employment: Recommendations for successful implementation in South Africa. *South African Journal of Occupational Therapy*, *41*, 85–90.	This article presents findings from a descriptive qualitative study where focus a group interview was used to explore supported employment (SE) among service providers who had initiated SE programmes in South Africa. The focus group centred on questions regarding barriers to successful implementation and adaptations required to make SE work to facilitate employment of people with disabilities. The authors present findings from a thematic analysis of the focus group data and make recommendations for the successful implementation of SE services in South Africa.	Not an impact evaluation
Mactaggart, I., Banks, L. M., Kuper, H., Murthy, G. V. S., Sagar, J., Oye, J., & Polack, S. (2018). Livelihood opportunities amongst adults with and without disabilities in Cameroon and India: A case control study. *PloS one*, *13*(4), e0194105.	This article presents findings from a population‐based case–control study of adults with and without disabilities in North‐West Cameroon and in Telangana State, India to examine livelihood opportunities amongst adults with disabilities. The authors found that adults with disabilities were five times less likely to be working compared to age‐sex matched controls in both settings, and also present key predictors of working. The paper concludes with recommendations for inclusive programming in LMIC to improve livelihood prospects for adults with disabilities.	Not an impact evaluation
Rath, S., Prost, A., Samal, S., Pradhan, H., Copas, A., Gagrai, S., Rath, S., Gope, R.K., Nair, N., Tripathy, P. & Bhatia, K. (2020). Community youth teams facilitating participatory adolescent groups, youth leadership activities and livelihood promotion to improve school attendance, dietary diversity and mental health among adolescent girls in rural eastern India: protocol for a cluster‐randomised controlled trial. *Trials*, *21*(1), 1–14.	In this protocol, the authors describe the evaluation of the Jharkhand Initiative for Adolescent Health (JIAH), a community intervention which aims to improve school attendance, dietary diversity and mental health among adolescent girls aged 10–19 years in rural Jharkhand, eastern India.	Protocol; Ineligible population; No livelihoods outcomes
Johannsmeier, C. (2007). *The social and economic effects of the disability grant for people with disabilities and their households: A qualitative study in KwaZulu‐Natal Province* (Doctoral dissertation).	This Doctoral Dissertation explores the social and economic effects of he South African Disability Grant (DG). The study employed qualitative and participatory methods to explore the impact of the DG among people with physical, visual and hearing disabilities who are DG recipients, in eight urban and rural areas of KwaZulu Natal Province of South Africa. The findings, which are qualitative, highlight interactions between the DG recipients, their households, and the physical and attitudinal barriers they face. The Dissertation concludes with a thorough discussion of implications for social protection planning and programming.	Cross‐sectional; Descriptive
Shimizu, M., Yi, S., Tuot, S., Suong, S., Sron, S., Shibanuma, A., & Jimba, M. (2016). The impact of a livelihood programme on depressive symptoms among people living with HIV in Cambodia. *Global Health Action*, *9*(1), 31999.	This article presents findings from a quasi‐experimental, nonequivalent comparison group study conducted in Cambodia to examine the impact of a livelihood programme on depressive symptoms and associated factors among people living with HIV. Data were collected from an intervention group comprising 357 people living with HIV who had participated in the livelihood programme and a comparison group comprising 328 people living with HIV who had not participated in this programme. The authors report that participants in the intervention group had significantly lower odds of having depressive symptoms than those in the control. Recommendations for programme scale up are made.	Ineligible population
Banks, L. M. (2019). *Investigating disability‐inclusion in social protection programmes in low‐and middle‐income countries, with case studies from Vietnam and Nepal* (Doctoral dissertation, London School of Hygiene & Tropical Medicine).	This Doctoral Dissertation is presented in the research paper style format, and as such includes several different but related journal articles that have been published in, accepted by, or submitted to peer‐reviewed journals. Each paper included in the Dissertation is centrally concerned with disability and poverty and social protection in LMIC. The document concludes with an analysis of the adequacy of social protection in meeting its intended aims for people with disabilities in LMIC.	This Dissertation comprises several peer‐reviewed papers which were examined individually for their relevance to this Systematic Review.
Anderson, W. L., Wiener, J. M., & Khatutsky, G. (2006). Workforce issues and consumer satisfaction in Medicaid personal assistance services. *Health Care Financing Review*, *28*(1), 87.	This article presents findings from a survey of older people and younger persons with disabilities who were receiving Medicaid‐financed home and community‐based services (HCBS), to explore the effect of workforce issues on consumer satisfaction. The authors report that recruitment problems had very strong negative and significant effects on consumer satisfaction. They go on to discuss other key indicators of consumer satisfaction among people with disabilities. They conclude with recommendations for HCDSs.	Not an impact evaluation
Drosos, N., & Theodoroulakis, M. (2019). Employment as an Integral Part of Social Inclusion: The Case of Mental Health Patients in Greece. In *Promoting Social Inclusion*. Emerald Publishing Limited.	This chapter described an approach to career counselling which combines elements from the Individual Placement and Support (IPS) model with contemporary career theories that have been developed to address challenges in the world of work. The chapter reflects on this model—developed by the Pan‐Hellenic Association for Psychosocial Rehabilitation and Work Integration (PEPSAEE) in Greece—and discusses implications for the model's implementation in vocational rehabilitation of mental health service users.	Not an impact evaluation
Paul, N., Guleria, B., & Gupta, S. (2019). A comprehensive study of community‐based inclusion, rehabilitation, and multidisciplinary approach toward cross‐disabilities in panchayats of North India. *The Indian Journal of Occupational Therapy*, *51*(3), 77.	This article reports the findings from a study which explored the Community‐Based Inclusion and Rehabilitation (CBIR) programme model of the Chinmaya Organisation for Rural Development (CORD) in India. This mixed methods study presents descriptive data on a sample of people with disabilities and conclude the people with disabilities are marginalised and require supportive programming and make recommendations for the implementation of CBIR models.	Not an impact evaluation
Higashida, M. (2019). Experiences of disabled people making the transition from vocational training to employment in Sri Lanka: An exploratory study. Asia Pacific *Journal of Social Work and Development*, *29*(3), 194–208.	This study presents findings from a qualitative study examining the experiences of people with disabilities transitioning out of vocational training programmes into employment, in Sri Lanka. Drawing on data from interviews with 12 people with disabilities, the study suggests that gain work satisfaction, earn and spend income, expand social relationships, and face challenges, following vocational training. Implications for policy and practice are discussed.	Qualitative
Makanya, M. W., Runo, M., & Wawire, V. (2014). Effectiveness of transitional and follow‐up programmes to community integration of young adults with intellectual disabilities (YAWID) in Kiambu County, Kenya. *Journal of the American Academy of Special Education Professionals*, *87*, 106.	This study describes how vocational education and transitional services offered in vocational institutions affect young adults with intellectual disabilities' community integration. This case study employs mixed methods to present a description of service users' experiences of the programming. The author discusses the implications of the findings for developing a more appropriate vocational training and transitional services programme.	Not an impact evaluation
Kallio, S. (2019). Improving the livelihoods of persons with disabilities through income generating activities: Towards more effective and sustainable development projects in Sierra Leone.	This Masters Thesis presents findings from a which sought to evaluate the effectiveness and sustainability of income generation projects in Sierra Leone, and to identify the factors influencing the livelihoods of persons with disabilities in Sierra Leone. Drawing on semi‐structured interviews and a focus group discussion with adult persons with disabilities who had participated the programme, the Thesis shows that limited financial assets and strong competition limit people with disabilities from expanding their businesses. Other findings are discussed and recommendations for successful poverty reduction and livelihood programmes are made.	Qualitative
Berhane, G., Devereus, S., Hoddinott, J., Hoel, J., Kimmel, M., Ledlie, N., & Roelen, K. (2015). *Endline report*. ‘*Evaluation of the social cash transfer programme, Tigray Region, Ethiopia*’. International Food Policy Research Institute, Institute of Development Studies, and University of Mekelle, 194.	This paper presents findings from an impact evaluation of a Social Cash Transfer Pilot Programme (SCTPP) in Ethiopia. The programme, run in two *woredas*, aims to improve the quality of life for vulnerable children, older persons, and persons with disabilities. The paper reports that the SCTPP effectively communicated with beneficiaries, reached its target group and provided full transfers on a timely and consistent basis, improved household food security and reduced hunger, and had modest effects on schooling and asset formation. There were no large or measurable impacts on a range of other outcomes, including education.	Findings not disaggregated for people with disabilities
