# Supplementary material for: Effectiveness of interventions for improving livelihood outcomes for people with disabilities in low‐ and middle‐income countries: A systematic review
Source: Campbell Syst Rev. 2022 Jun 30;18(3):e1257. doi: 10.1002/cl2.1257 (PMC9246293; doi:10.1002/cl2.1257)
Supplement: Supplementary file 1 — Supporting information. [file CL2-18-e1257-s001.docx]

# Appendices

**Disability EGM Update Searches – February 2020**

1. **Ovid MEDLINE(R) and In-Process & Other Non-Indexed Citations and Daily <1946 to February 25, 2020>Searched 26^th^ February 2020**

1 ((disable* or disabilit* or handicapped) adj5 (person* or people or child* or adolescen* or women or mother* or maternal or group*)).ti,kw. (16354)

2 ((physical* or intellectual* or learning or psychiatric* or sensory or motor or neuromotor or cognitive or mental* or developmental or communication or learning) adj2 (disabilit* or disabl* or handicap*)).ti,kw. (20515)

3 ((cognitive* or learning or mobility or sensory or visual* or vision or sight or hearing or physical* or mental* or intellectual*) adj2 (impair* or disabilit* or disabl* or handicap*)).ti,kw. (47712)

4 ((communication or language or speech or learning) adj5 disorder*).ti,kw. (4172)

5 ((depression or depressive or anxiety or psychiat* or well-being or quality of life or self-esteem or self perception) adj2 (impair* or disabilit* or disabl* or handicap*)).ti,kw. (2717)

6 mental health.ti,kw. (56608)

7 ((schizophreni* or psychos* or psychotic or schizoaffective or schizophreniform or dementia* or alzheimer*) adj2 (impair* or disabilit* or disabl* or handicap*)).ti,kw. (2932)

8 ((mental* or emotional* or psychiatric or neurologic*) adj2 (disorder* or ill or illness*)).ti,kw. (37898)

9 (autis* or dyslexi* or Down* syndrome or mongolism or trisomy 21).ti,kw. (54829)

10 ((intellectual* or educational* or mental* or psychological* or developmental) adj5 (impair* or retard* or deficien* or disable* or disabili* or handicap* or ill*)).ti,kw. (43259)

11 (((hearing or acoustic or ear*) adj5 (loss* or impair* or deficien* or disable* or disabili* or handicap*)) or deaf*).ti,kw. (43401)

12 (((visual* or vision or eye* or ocular) adj5 (loss* or impair* or deficien* or disable* or disabili* or handicap*)) or blind*).ti,kw. (73473)

13 ((cerebral pals* or spina bifida or muscular dystroph* or arthriti* or osteogenesis imperfecta or musculoskeletal abnormalit* or musculo-skeletal abnormalit* or muscular abnormalit* or skeletal abnormalit* or limb abnormalit* or brain injur* or amput* or clubfoot or polio* or paraplegi* or paralys* or paralyz* or hemiplegi* or stroke* or cerebrovascular accident*) adj2 (impair* or disabilit* or disabl* or handicap*)).ti,kw. (1278)

14 (physical* adj5 (impair* or deficien* or disable* or disabili* or handicap*)).ti,kw. (4015)

15 [exp disabled persons/ed, pc, rh, st, td, ut] (0)

16 exp *intellectual disability/ep, mo, pc or exp *developmental disabilities/ep, mo, pc or exp *child development disorders, pervasive/ep, mo, pc or exp *communication disorders/ep, mo, pc (9246)

17 exp *cerebral palsy/ep, mo, pc or exp *spina bifida cystica/ep, mo, pc or exp *spina bifida occulta/ep, mo, pc or exp *muscular dystrophies/ep, mo, pc or exp *arthritis/ep, mo, pc or exp *osteogenesis imperfecta/ep, mo, pc or exp *musculoskeletal abnormalities/ep, mo, pc or exp *brain injuries/ep, mo, pc or exp *amputation/ep, mo, pc or exp *clubfoot/ep, mo, pc or exp *poliomyelitis/ep, mo, pc or exp *paraplegia/ep, mo, pc or exp *hemiplegia/ep, mo, pc or exp *stroke/ep, mo, pc (43035)

18 exp *hearing loss/ep, mo, pc or exp *vision, low/ep, mo, pc or exp *deafness/ep, mo, pc or exp *blindness/ep, mo, pc (6464)

19 exp *"schizophrenia and disorders with psychotic features"/ep, mo, pc or exp *dementia/ep, mo, pc or exp *alzheimer disease/ep, mo, pc (17042)

20 exp *mental disorders/ep, mo, pc (119244)

21 exp *neurodevelopmental disorders/ep, mo, pc (12457)

22 or/1-20 (477974)

23 Developing Countries.sh,kf. (85006)

24 Africa/ or Asia/ or Caribbean/ or West Indies/ or Middle East/ or South America/ or Latin America/ or Central America/ (80823)

25 (Africa or Asia or Caribbean or West Indies or Middle East or South America or Latin America or Central America).tw. (190173)

26 (Afghanistan or Albania or Algeria or Angola or Argentina or Armenia or Armenian or Azerbaijan or Bangladesh or Benin or Byelarus or Byelorussian or Belarus or Belorussian or Belorussia or Belize or Bhutan or Bolivia or Bosnia or Herzegovina or Hercegovina or Botswana or Brazil or Bulgaria or Burkina Faso or Burkina Fasso or Upper Volta or Burundi or Urundi or Cambodia or Khmer Republic or Kampuchea or Cameroon or Cameroons or Cameron or Camerons or Cape Verde or Central African Republic or Chad or China or Colombia or Comoros or Comoro Islands or Comores or Mayotte or Congo or Zaire or Costa Rica or Cote d'Ivoire or Ivory Coast or Cuba or Djibouti or French Somaliland or Dominica or Dominican Republic or East Timor or East Timur or Timor Leste or Ecuador or Egypt or United Arab Republic or El Salvador or Eritrea or Ethiopia or Fiji or Gabon or Gabonese Republic or Gambia or Gaza or Georgia Republic or Georgian Republic or Ghana or Grenada or Guatemala or Guinea or Guiana or Guyana or Haiti or Honduras or India or Maldives or Indonesia or Iran or Iraq or Jamaica or Jordan or Kazakhstan or Kazakh or Kenya or Kiribati or Korea or Kosovo or Kyrgyzstan or Kirghizia or Kyrgyz Republic or Kirghiz or Kirgizstan or Lao PDR or Laos or Lebanon or Lesotho or Basutoland or Liberia or Libya or Macedonia or Madagascar or Malagasy Republic or Malaysia or Malaya or Malay or Sabah or Sarawak or Malawi or Mali or Marshall Islands or Mauritania or Mauritius or Agalega Islands or Mexico or Micronesia or Middle East or Moldova or Moldovia or Moldovian or Mongolia or Montenegro or Morocco or Ifni or Mozambique or Myanmar or Myanma or Burma or Namibia or Nepal or Netherlands Antilles or Nicaragua or Niger or Nigeria or Muscat or Pakistan or Palau or Palestine or Panama or Paraguay or Peru or Philippines or Philipines or Phillipines or Phillippines or Papua New Guinea or Romania or Rumania or Roumania or Rwanda or Ruanda or Saint Lucia or St Lucia or Saint Vincent or St Vincent or Grenadines or Samoa or Samoan Islands or Navigator Island or Navigator Islands or Sao Tome or Senegal or Serbia or Montenegro or Seychelles or Sierra Leone or Sri Lanka or Solomon Islands or Somalia or Sudan or Suriname or Surinam or Swaziland or South Africa or Syria or Tajikistan or Tadzhikistan or Tadjikistan or Tadzhik or Tanzania or Thailand or Togo or Togolese Republic or Tonga or Tunisia or Turkey or Turkmenistan or Turkmen or Uganda or Ukraine or Uzbekistan or Uzbek or Vanuatu or New Hebrides or Venezuela or Vietnam or Viet Nam or West Bank or Yemen or Zambia or Zimbabwe).tw. (1020832)

27 exp africa/ or algeria/ or egypt/ or libya/ or morocco/ or tunisia/ or cameroon/ or central african republic/ or chad/ or congo/ or "democratic republic of the congo"/ or equatorial guinea/ or gabon/ or burundi/ or djibouti/ or eritrea/ or ethiopia/ or kenya/ or rwanda/ or somalia/ or south sudan/ or sudan/ or tanzania/ or uganda/ or angola/ or botswana/ or lesotho/ or malawi/ or mozambique/ or namibia/ or south africa/ or swaziland/ or zambia/ or zimbabwe/ or benin/ or burkina faso/ or cape verde/ or cote d'ivoire/ or gambia/ or ghana/ or guinea/ or guinea-bissau/ or liberia/ or mali/ or mauritania/ or niger/ or nigeria/ or senegal/ or sierra leone/ or togo/ or americas/ or exp caribbean region/ or exp west indies/ or exp central america/ or belize/ or costa rica/ or el salvador/ or guatemala/ or honduras/ or nicaragua/ or panama/ or panama canal zone/ or latin america/ or mexico/ or exp south america/ or argentina/ or bolivia/ or brazil/ or chile/ or colombia/ or ecuador/ or french guiana/ or guyana/ or paraguay/ or peru/ or suriname/ or uruguay/ or venezuela/ or asia/ or asia, central/ or kazakhstan/ or kyrgyzstan/ or tajikistan/ or turkmenistan/ or uzbekistan/ or exp asia, southeastern/ or borneo/ or brunei/ or cambodia/ or timor-leste/ or indonesia/ or laos/ or malaysia/ or mekong valley/ or myanmar/ or philippines/ or singapore/ or thailand/ or vietnam/ or asia, western/ or bangladesh/ or bhutan/ or india/ or sikkim/ or middle east/ or afghanistan/ or bahrain/ or iran/ or iraq/ or israel/ or jordan/ or kuwait/ or lebanon/ or oman/ or qatar/ or saudi arabia/ or syria/ or turkey/ or united arab emirates/ or yemen/ or nepal/ or pakistan/ or sri lanka/ or far east/ or china/ or beijing/ or macau/ or tibet/ or korea/ or mongolia/ or taiwan/ or indian ocean islands/ or comoros/ or madagascar/ or mauritius/ or reunion/ or seychelles/ or pacific islands/ or exp melanesia/ or exp micronesia/ or polynesia/ or pitcairn island/ or exp samoa/ or tonga/ or prince edward island/ or west indies/ or "antigua and barbuda"/ or bahamas/ or barbados/ or cuba/ or dominica/ or dominican republic/ or grenada/ or guadeloupe/ or haiti/ or jamaica/ or martinique/ or netherlands antilles/ or puerto rico/ or "saint kitts and nevis"/ or saint lucia/ or "saint vincent and the grenadines"/ or "trinidad and tobago"/ or united states virgin islands/ or oceania/ (1095245)

28 ((developing or less* developed or under developed or underdeveloped or middle income or low* income or underserved or under served or deprived or poor*) adj (countr* or nation? or population? or world or state*)).ti,ab. (95470)

29 ((developing or less* developed or under developed or underdeveloped or middle income or low* income) adj (economy or economies)).ti,ab. (507)

30 (low* adj (gdp or gnp or gross domestic or gross national)).tw. (236)

31 (low adj3 middle adj3 countr*).tw. (14542)

32 (lmic or lmics or third world or lami countr*).tw. (6850)

33 transitional countr*.tw. (156)

34 or/23-33 (1649612)

35 ((systematic* or synthes*) adj3 (research or evaluation* or finding* or thematic* or report or descriptive or explanatory or narrative or meta* or review* or data or literature or studies or evidence or map or quantitative or study or studies or paper or impact or impacts or effect* or compar*)).ti,ab,kw. (328129)

36 ("meta regression" or "meta synth*" or "meta-synth*" or "meta analy*" or "metaanaly*" or "meta-analy*" or "metanaly*" or "metaregression" or "metaregression" or "methodologic* overview" or "pool* analys*" or "pool* data" or "quantitative* overview" or "research integration").ti,ab,kw. (174826)

37 (review adj3 (effectiveness or effects or systemat* or synth* or integrat* or map* or methodologic* or quantitative or evidence or literature)).ti,ab,kw. (404761)

38 ("meta ethnograph*" or "meta synthesis" or (synthesis and ("qualitative literature" or "qualitative research")) or "critical interpretive synthesis" or ("systematic review" and ("qualitative research" or "qualitative literature" or "qualitative stud*")) or "thematic synthesis" or "framework synthesis" or "realist review" or "realist synthesis" or "qualitative systematic review*" or "qualitative evidence synthes*" or (("quality assessment" or "critical appraisal" or "literature search*") and ("qualitative research" or "qualitative literature" or "qualitative stud*")) or (Noblit and Hare) or "meta narrative*" or "narrative synthesis").ti,ab,kw. (7484)

39 meta-analysis/ or evaluation studies/ or qualitative research/ or systematic review/ (481597)

40 controlled clinical trial/ or randomized controlled trial/ or equivalence trial/ or pragmatic clinical trial/ or case-control studies/ or retrospective studies/ or cohort studies/ or follow-up studies/ or longitudinal studies/ or prospective studies/ or epidemiologic methods/ or epidemiologic studies/ or controlled before-after studies/ or cross-sectional studies/ or interrupted time series analysis/ or control groups/ or cross-over studies/ or double-blind method/ or matched-pair analysis/ or meta-analysis as topic/ or random allocation/ or single-blind method/ or "retraction of publication"/ or case reports/ (4991800)

41 (random$ or placebo$ or single blind$ or double blind$ or triple blind$ or cohort$ or ((case$ or cohort or follow up or follow-up) adj2 (control$ or series or report$ or study or studies)) or retrospective$ or (observ$ adj3 (study or studies))).ti,ab,kw. (3137636)

42 or/35-41 (6813425)

43 22 and 34 and 42 (23469)

44 limit 43 to yr="2018 -Current" (**4067)**

1. **Embase Classic+Embase (Ovid) <1947 to 2020 February 25>Searched 26^th^ February 2020**

1 ((disable* or disabilit* or handicapped) adj5 (person* or people or child* or adolescen* or women or mother* or maternal or group*)).ti,kw. (23241)

2 ((physical* or intellectual* or learning or psychiatric* or sensory or motor or neuromotor or cognitive or mental* or developmental or communication or learning) adj2 (disabilit* or disabl* or handicap*)).ti,kw. (31740)

3 ((cognitive* or learning or mobility or sensory or visual* or vision or sight or hearing or physical* or mental* or intellectual*) adj2 (impair* or disabilit* or disabl* or handicap*)).ti,kw. (81539)

4 ((communication or language or speech or learning) adj5 disorder*).ti,kw. (7284)

5 ((depression or depressive or anxiety or psychiat* or well-being or quality of life or self-esteem or self perception) adj2 (impair* or disabilit* or disabl* or handicap*)).ti,kw. (3071)

6 mental health.ti,kw. (75380)

7 ((schizophreni* or psychos* or psychotic or schizoaffective or schizophreniform or dementia* or alzheimer*) adj2 (impair* or disabilit* or disabl* or handicap*)).ti,kw. (4009)

8 ((mental* or emotional* or psychiatric or neurologic*) adj2 (disorder* or ill or illness*)).ti,kw. (58575)

9 (autis* or dyslexi* or Down* syndrome or mongolism or trisomy 21).ti,kw. (73218)

10 ((intellectual* or educational* or mental* or psychological* or developmental) adj5 (impair* or retard* or deficien* or disable* or disabili* or handicap* or ill*)).ti,kw. (63629)

11 (((hearing or acoustic or ear*) adj5 (loss* or impair* or deficien* or disable* or disabili* or handicap*)) or deaf*).ti,kw. (61540)

12 (((visual* or vision or eye* or ocular) adj5 (loss* or impair* or deficien* or disable* or disabili* or handicap*)) or blind*).ti,kw. (103804)

13 ((cerebral pals* or spina bifida or muscular dystroph* or arthriti* or osteogenesis imperfecta or musculoskeletal abnormalit* or musculo-skeletal abnormalit* or muscular abnormalit* or skeletal abnormalit* or limb abnormalit* or brain injur* or amput* or clubfoot or polio* or paraplegi* or paralys* or paralyz* or hemiplegi* or stroke* or cerebrovascular accident*) adj2 (impair* or disabilit* or disabl* or handicap*)).ti,kw. (1379)

14 (physical* adj5 (impair* or deficien* or disable* or disabili* or handicap*)).ti,kw. (5799)

15 exp *disability/dm, ep, pc, rh, th [Disease Management, Epidemiology, Prevention, Rehabilitation, Therapy] (3305)

16 exp *mental disease/dm, ep, pc, rh, th [Disease Management, Epidemiology, Prevention, Rehabilitation, Therapy] (241319)

17 exp *"disorders of higher cerebral function"/dm, ep, pc, rh, th [Disease Management, Epidemiology, Prevention, Rehabilitation, Therapy] (46137)

18 exp *developmental disorder/dm, ep, pc, rh, th [Disease Management, Epidemiology, Prevention, Rehabilitation, Therapy] (2226)

19 exp *disabled person/ (27928)

20 *cerebral palsy/dm, ep, pc, rh, th [Disease Management, Epidemiology, Prevention, Rehabilitation] (4546)

21 exp *spinal dysraphism/dm, ep, pc, rh, th [Disease Management, Epidemiology, Prevention, Rehabilitation, Therapy] (470)

22 exp *neural tube defect/dm, ep, pc, rh, th [Disease Management, Epidemiology, Prevention, Rehabilitation, Therapy] (2833)

23 exp *dystrophy/dm, ep, pc, rh, th [Disease Management, Epidemiology, Prevention, Rehabilitation, Therapy] (3481)

24 exp *arthritis/dm, ep, pc, rh, th [Disease Management, Epidemiology, Prevention, Rehabilitation, Therapy] (28190)

25 *osteogenesis imperfecta/dm, ep, pc, rh, th [Disease Management, Epidemiology, Prevention, Rehabilitation, Therapy] (299)

26 exp *musculoskeletal system malformation/dm, ep, pc, rh, th [Disease Management, Epidemiology, Prevention, Rehabilitation, Therapy] (5142)

27 exp *limb malformation/dm, ep, pc, rh, th [Disease Management, Epidemiology, Prevention, Rehabilitation, Therapy] (2389)

28 exp *brain malformation/dm, ep, pc, rh, th [Disease Management, Epidemiology, Prevention, Rehabilitation, Therapy] (2655)

29 exp *limb amputation/ (6728)

30 exp *clubfoot/dm, ep, pc, rh, th [Disease Management, Epidemiology, Prevention, Rehabilitation, Therapy] (697)

31 exp *poliomyelitis/dm, ep, pc, rh, th [Disease Management, Epidemiology, Prevention, Rehabilitation, Therapy] (4509)

32 exp *paralysis/dm, ep, pc, rh, th [Disease Management, Epidemiology, Prevention, Rehabilitation, Therapy] (18455)

33 exp *cerebrovascular accident/dm, ep, pc, rh, th [Disease Management, Epidemiology, Prevention, Rehabilitation, Therapy] (14445)

34 exp *hearing disorder/dm, ep, pc, rh, th [Disease Management, Epidemiology, Prevention, Rehabilitation, Therapy] (12848)

35 exp *hearing impairment/dm, ep, pc, rh, th [Disease Management, Epidemiology, Prevention, Rehabilitation, Therapy] (9118)

36 exp *visual disorder/dm, ep, pc, rh, th [Disease Management, Epidemiology, Prevention, Rehabilitation, Therapy] (13295)

37 or/1-36 (800216)

38 developing country/ (95013)

39 low income country/ (5340)

40 middle income country/ (7663)

41 ((developing or less* developed or under developed or underdeveloped or middle income or low* income or underserved or under served or deprived or poor*) adj (economy or economies)).ti,ab. (801)

42 ((developing or less* developed or under developed or underdeveloped or middle income or low* income or underserved or under served or deprived or poor*) adj (countr* or nation? or population? or world)).ti,ab. (124055)

43 (low* adj (gdp or gnp or gross domestic or gross national)).ti,ab. (344)

44 (low adj3 middle adj3 countr*).ti,ab. (17826)

45 (lmic or lmics or third world or lami countr*).ti,ab. (8820)

46 transitional countr*.ti,ab. (226)

47 global south.ti,ab. (351)

48 "Africa south of the Sahara"/ (13771)

49 ("africa south of the sahara" or sub-saharan africa or central africa or eastern africa or southern africa or western africa).ti,ab. (32858)

50 Botswana/ (2676)

51 (Botswana or Bechuanaland or Kalahari).ti,ab. (2905)

52 Equatorial Guinea/ (467)

53 (Equatorial Guinea or Spanish Guinea).ti,ab. (563)

54 Gabon/ (1749)

55 (Gabon or Gabonese Republic).ti,ab. (1919)

56 Mauritius/ (892)

57 (Mauritius or Agalega Islands).ti,ab. (1035)

58 Namibia/ (1579)

59 Namibia.ti,ab. (1611)

60 South Africa/ (51310)

61 South Africa.ti,ab. (39328)

62 Angola/ (1461)

63 angola.ti,ab. (1570)

64 Cameroon/ (7093)

65 Cameroon.ti,ab. (7783)

66 Cape Verde/ (353)

67 (Cape Verde or Cabo Verde).ti,ab. (598)

68 Congo/ (4008)

69 (congo not ((democratic republic adj3 congo) or congo red or crimean-congo)).ti,ab. (3388)

70 Cote d'Ivoire/ (3321)

71 (Cote d'Ivoire or Ivory Coast).ti,ab. (4378)

72 Ghana/ (11563)

73 (Ghana or Gold Coast).ti,ab. (12283)

74 Kenya/ (21482)

75 kenya.mp. (25409)

76 Lesotho/ (697)

77 (Lesotho or Basutoland).ti,ab. (767)

78 Mauritania/ (638)

79 Mauritania.ti,ab. (670)

80 Nigeria/ (38425)

81 Nigeria.ti,ab. (35599)

82 "Sao Tome and Principe"/ (77)

83 (sao tome adj2 principe).ti,ab. (155)

84 Sudan/ (7300)

85 (Sudan not south sudan).ti,ab. (9827)

86 Swaziland/ (915)

87 Swaziland.ti,ab. (943)

88 Zambia/ (6263)

89 (Zambia or Northern Rhodesia).ti,ab. (5971)

90 Benin/ (2499)

91 (Benin or Dahomey).ti,ab. (4713)

92 Burkina Faso/ (4263)

93 (Burkina Faso or Burkina Fasso or Upper Volta).ti,ab. (4940)

94 Burundi/ (852)

95 Burundi.ti,ab. (887)

96 Central African Republic/ (927)

97 (Central African Republic or Ubangi-Shari).ti,ab. (1108)

98 Chad/ (994)

99 Chad.ti,ab. (1402)

100 Comoros/ (332)

101 (Comoros or Comoro Islands or Mayotte or Iles Comores).ti,ab. (608)

102 "Democratic Republic Congo"/ (4183)

103 ((democratic republic adj2 congo) or belgian congo or zaire).ti,ab. (4885)

104 Eritrea/ (557)

105 Eritrea.ti,ab. (651)

106 Ethiopia/ (16392)

107 Ethiopia.ti,ab. (15662)

108 Gambia/ (2816)

109 Gambia.ti,ab. (2543)

110 Guinea/ (2716)

111 (Guinea not (New Guinea or Guinea Pig* or Guinea Fowl)).ti,ab. (4748)

112 Guinea-Bissau/ (1086)

113 (Guinea-Bissau or Portuguese Guinea).ti,ab. (1124)

114 Liberia/ (1781)

115 Liberia.ti,ab. (1793)

116 Madagascar/ (4596)

117 (Madagascar or Malagasy Republic).ti,ab. (5272)

118 Malawi/ (7318)

119 (Malawi or Nyasaland).ti,ab. (7711)

120 Mali/ (3518)

121 Mali.ti,ab. (4351)

122 Mozambique/ (3717)

123 (Mozambique or Mocambique or Portuguese East Africa).ti,ab. (4027)

124 Niger/ (2444)

125 (Niger not (Aspergillus or Peptococcus or Schizothorax or Cruciferae or Gobius or Lasius or Agelastes or Melanosuchus or radish or Parastromateus or Orius or Apergillus or Parastromateus or Stomoxys)).ti,ab. (4238)

126 Rwanda/ (3571)

127 (Rwanda or Ruanda).ti,ab. (3537)

128 Senegal/ (6989)

129 senegal.ti,ab. (6820)

130 Sierra Leone/ (2344)

131 Sierra Leone.mp. (3003)

132 exp Somalia/ (2010)

133 Somalia.ti,ab. (1454)

134 South Sudan/ (274)

135 south sudan.ti,ab. (597)

136 Tanzania/ (15236)

137 (Tanzania or Tanganyika or Zanzibar).ti,ab. (15351)

138 Togo/ (1458)

139 (Togo or Togolese Republic).ti,ab. (1669)

140 Uganda/ (18170)

141 Uganda.ti,ab. (17168)

142 Zimbabwe/ (6973)

143 (Zimbabwe or Rhodesia).ti,ab. (6427)

144 Maldives/ (277)

145 Maldives.ti,ab. (324)

146 Algeria/ (4938)

147 Algeria.ti,ab. (4911)

148 Iran/ (49002)

149 Iran.ti,ab. (50966)

150 exp Iraq/ (8629)

151 Iraq.ti,ab. (9387)

152 Jordan/ (6583)

153 Jordan.ti,ab. (7808)

154 Lebanon/ (5802)

155 Lebanon.ti,ab. (5437)

156 Libyan Arab Jamahiriya/ (1705)

157 Libya.ti,ab. (1501)

158 Argentina/ (22507)

159 Argentina.ti,ab. (21909)

160 Belize/ (775)

161 Belize.ti,ab. (858)

162 exp Brazil/ (113311)

163 Brazil.ti,ab. (99808)

164 Colombia/ (19388)

165 Colombia.ti,ab. (16497)

166 Costa Rica/ (4882)

167 Costa Rica.ti,ab. (5140)

168 Cuba/ (6958)

169 Cuba.ti,ab. (5931)

170 Dominica/ (185)

171 Dominica.ti,ab. (522)

172 Dominican Republic/ (2504)

173 Dominican Republic.ti,ab. (2297)

174 Ecuador/ (5348)

175 Ecuador.ti,ab. (5330)

176 Grenada/ (261)

177 Grenada.ti,ab. (372)

178 Guyana/ (1026)

179 Guyana.mp. (1578)

180 Jamaica/ (4350)

181 Jamaica.ti,ab. (3990)

182 Mexico/ (46440)

183 Mexico.ti,ab. (51211)

184 exp Panama/ (3314)

185 Panama.ti,ab. (4295)

186 Paraguay/ (1486)

187 Paraguay.mp. (2190)

188 Peru/ (12235)

189 Peru.ti,ab. (12771)

190 Saint Lucia/ (123)

191 (St Lucia or Saint Lucia).ti,ab. (385)

192 "Saint Vincent and the Grenadines"/ (72)

193 Grenadines.ti,ab. (88)

194 Suriname/ (1259)

195 Suriname.ti,ab. (686)

196 Venezuela/ (6804)

197 Venezuela.ti,ab. (6557)

198 Albania/ (1816)

199 Albania.ti,ab. (1661)

200 Azerbaijan/ (1832)

201 Azerbaijan.ti,ab. (1941)

202 Belarus/ (2831)

203 (belarus or byelarus or belorussia).ti,ab. (2243)

204 exp "Bosnia and Herzegovina"/ (2723)

205 (bosnia or herzegovina).ti,ab. (2923)

206 Bulgaria/ (9799)

207 Bulgaria.ti,ab. (6723)

208 Croatia/ (9946)

209 croatia.ti,ab. (9492)

210 Kazakhstan/ (3943)

211 (Kazakhstan or kazakh).ti,ab. (4003)

212 "Macedonia (Republic)"/ (1107)

213 Macedonia.ti,ab. (1939)

214 "Montenegro (republic)"/ (718)

215 Montenegro.ti,ab. (1135)

216 Romania/ (14558)

217 Romania.ti,ab. (9271)

218 exp Russian Federation/ (63691)

219 USSR/ (48320)

220 (Russia or Russian Federation or USSR or Union of Soviet Socialist Republics or Soviet Union).mp. (121177)

221 exp Serbia/ (5714)

222 serbia.ti,ab. (6678)

223 "Turkey (republic)"/ (36041)

224 turkey.ti,ab. not animal/ (42306)

225 Turkmenistan/ (630)

226 Turkmenistan.ti,ab. (369)

227 Yugoslavia/ (9598)

228 yugoslavia.ti,ab. (2903)

229 exp Samoan Islands/ (981)

230 american samoa.ti,ab. (412)

231 exp China/ (221199)

232 china.ti,ab. (200641)

233 Fiji/ (1509)

234 fiji.ti,ab. (2109)

235 Malaysia/ (21974)

236 malaysia.ti,ab. (20542)

237 Marshall Islands/ (165)

238 marshall islands.ti,ab. (348)

239 Nauru/ (67)

240 nauru.ti,ab. (164)

241 ("independent state of samoa" or (samoa not american samoa) or western samoa or navigator islands or samoan islands).ti,ab. (634)

242 Thailand/ (33871)

243 Thailand.ti,ab. (31471)

244 Tonga/ (360)

245 tonga.ti,ab. (470)

246 Tuvalu/ (45)

247 Tuvalu.ti,ab. (64)

248 Bangladesh/ (15971)

249 Bangladesh.ti,ab. (15950)

250 Bhutan/ (772)

251 Bhutan.ti,ab. (759)

252 exp India/ (150311)

253 India.ti,ab. (132004)

254 exp Pakistan/ (27273)

255 Pakistan.ti,ab. (23991)

256 Sri Lanka/ (8633)

257 Sri Lanka.ti,ab. (7623)

258 Djibouti/ (348)

259 (Djibouti or French Somaliland).ti,ab. (438)

260 Egypt/ (21006)

261 Egypt.ti,ab. (18683)

262 Jordan/ (6583)

263 Jordan.ti,ab. (7808)

264 Morocco/ (7791)

265 Morocco.ti,ab. (7288)

266 Syrian Arab Republic/ (2609)

267 (Syria or Syrian Arab Republic).ti,ab. (2362)

268 Tunisia/ (9911)

269 tunisia.mp. (12045)

270 Palestine/ (1670)

271 Gaza.ti,ab. (1223)

272 Yemen/ (2034)

273 Yemen.ti,ab. (2093)

274 Bolivia/ (3567)

275 Bolivia.ti,ab. (3704)

276 El Salvador/ (1944)

277 El Salvador.ti,ab. (1528)

278 Guatemala/ (4528)

279 Guatemala.ti,ab. (4254)

280 Honduras/ (1973)

281 Honduras.ti,ab. (2064)

282 Nicaragua/ (2255)

283 Nicaragua.ti,ab. (2241)

284 Armenia/ (1985)

285 Armenia.ti,ab. (1533)

286 "Georgia (Republic)"/ (1969)

287 Kosovo/ (496)

288 Kosovo.ti,ab. (1225)

289 Kyrgyzstan/ (1656)

290 (kyrgyzstan or kyrgyz republic or kirghizia or kirghiz).ti,ab. (1257)

291 Moldova/ (1169)

292 Moldova.ti,ab. (886)

293 Tajikistan/ (965)

294 tajikistan.ti,ab. (690)

295 exp Ukraine/ (16664)

296 Ukraine.ti,ab. (6681)

297 Uzbekistan/ (2223)

298 Uzbekistan.ti,ab. (1483)

299 Cambodia/ (4970)

300 cambodia.ti,ab. (4637)

301 exp Indonesia/ (17860)

302 indonesia.ti,ab. (17231)

303 Kiribati/ (112)

304 Kiribati.ti,ab. (181)

305 Laos/ (2166)

306 (laos or (lao adj1 democratic republic)).ti,ab. (2081)

307 "Marshall Islands"/ (165)

308 "Federated States of Micronesia"/ (956)

309 (marshall island* or caroline island* or ellice island* or gilbert island* or johnston island* or mariana island* or micronesia or pacific island*).ti,ab. (8316)

310 Mongolia/ (3163)

311 mongolia.ti,ab. (4696)

312 Myanmar/ (3882)

313 (myanmar or burma).ti,ab. (4557)

314 Papua New Guinea/ (6672)

315 Papua New Guinea.ti,ab. (4829)

316 Philippines/ (11887)

317 Philippines.ti,ab. (10107)

318 Timor-Leste/ (569)

319 Timor-Leste.ti,ab. (368)

320 Vanuatu/ (448)

321 Vanuatu.ti,ab. (655)

322 Viet Nam/ (16683)

323 (Viet Nam or vietnam).ti,ab. (17580)

324 Afghanistan/ (5860)

325 Afghanistan.ti,ab. (6831)

326 Nepal/ (11509)

327 Nepal.ti,ab. (11194)

328 Haiti/ (4193)

329 Haiti.ti,ab. (3779)

330 "North Korea"/ (558)

331 (north korea or (democratic people* republic adj2 korea)).ti,ab. (455)

332 or/38-331 [ALL LMICs] (1746480)

333 ((systematic* or synthes*) adj3 (research or evaluation* or finding* or thematic* or report or descriptive or explanatory or narrative or meta* or review* or data or literature or studies or evidence or map or quantitative or study or studies or paper or impact or impacts or effect* or compar*)).ti,ab,kw. (421979)

334 ("meta regression" or "meta synth*" or "meta-synth*" or "meta analy*" or "metaanaly*" or "meta-analy*" or "metanaly*" or "metaregression" or "metaregression" or "methodologic* overview" or "pool* analys*" or "pool* data" or "quantitative* overview" or "research integration").ti,ab,kw. (244165)

335 (review adj3 (effectiveness or effects or systemat* or synth* or integrat* or map* or methodologic* or quantitative or evidence or literature)).ti,ab,kw. (523786)

336 ("meta ethnograph*" or "meta synthesis" or (synthesis and ("qualitative literature" or "qualitative research")) or "critical interpretive synthesis" or ("systematic review" and ("qualitative research" or "qualitative literature" or "qualitative stud*")) or "thematic synthesis" or "framework synthesis" or "realist review" or "realist synthesis" or "qualitative systematic review*" or "qualitative evidence synthes*" or (("quality assessment" or "critical appraisal" or "literature search*") and ("qualitative research" or "qualitative literature" or "qualitative stud*")) or (Noblit and Hare) or "meta narrative*" or "narrative synthesis").ti,ab,kw. (9028)

337 "systematic review"/ or meta analysis/ or exp evaluation study/ or qualitative analysis/ or qualitative research/ (515422)

338 (random$ or placebo$ or single blind$ or double blind$ or triple blind$ or cohort$ or ((case$ or cohort or follow up or follow-up) adj2 (control$ or series or report$ or study or studies)) or retrospective$ or (observ$ adj3 (study or studies))).ti,ab,kw. (4777384)

339 controlled clinical trial/ or randomized controlled trial/ or case control study/ or population based case control study/ or retrospective study/ or cohort analysis/ or follow up/ or longitudinal study/ or prospective study/ or cross-sectional study/ or times series analysis/ or control group/ or randomization/ (4102624)

340 or/333-339 (7259102)

341 37 and 332 and 340 (21356)

342 limit 341 to yr="2018 -Current" (4482)

343 limit 342 to embase (**3275**)

1. **PsycINFO (Ovid) <1806 to February Week 3 2020>Searched 26^th^ February 2020**

1 ((disable* or disabilit* or handicapped) adj5 (person* or people or child* or adolescen* or women or mother* or maternal or group*)).ti,hw. (22522)

2 ((physical* or intellectual* or learning or psychiatric* or sensory or motor or neuromotor or cognitive or mental* or developmental or communication or learning) adj2 (disabilit* or disabl* or handicap*)).ti,hw. (48731)

3 ((cognitive* or learning or mobility or sensory or visual* or vision or sight or hearing or physical* or mental* or intellectual*) adj2 (impair* or disabilit* or disabl* or handicap*)).ti,hw. (81483)

4 ((communication or language or speech or learning) adj5 disorder*).ti,hw. (19968)

5 ((depression or depressive or anxiety or psychiat* or well-being or quality of life or self-esteem or self perception) adj2 (impair* or disabilit* or disabl* or handicap*)).ti,hw. (1756)

6 mental health.ti,hw. (126256)

7 ((schizophreni* or psychos* or psychotic or schizoaffective or schizophreniform or dementia* or alzheimer*) adj2 (impair* or disabilit* or disabl* or handicap*)).ti,hw. (2036)

8 ((mental* or emotional* or psychiatric or neurologic*) adj2 (disorder* or ill or illness*)).ti,hw. (97032)

9 (autis* or dyslexi* or Down* syndrome or mongolism or trisomy 21).ti,hw. (56753)

10 ((intellectual* or educational* or mental* or psychological* or developmental) adj5 (impair* or retard* or deficien* or disable* or disabili* or handicap* or ill*)).ti,hw. (62656)

11 (((hearing or acoustic or ear*) adj5 (loss* or impair* or deficien* or disable* or disabili* or handicap*)) or deaf*).ti,hw. (20671)

12 (((visual* or vision or eye* or ocular) adj5 (loss* or impair* or deficien* or disable* or disabili* or handicap*)) or blind*).ti,hw. (20068)

13 ((cerebral pals* or spina bifida or muscular dystroph* or arthriti* or osteogenesis imperfecta or musculoskeletal abnormalit* or musculo-skeletal abnormalit* or muscular abnormalit* or skeletal abnormalit* or limb abnormalit* or brain injur* or amput* or clubfoot or polio* or paraplegi* or paralys* or paralyz* or hemiplegi* or stroke* or cerebrovascular accident*) adj2 (impair* or disabilit* or disabl* or handicap*)).ti,hw. (276)

14 (physical* adj5 (impair* or deficien* or disable* or disabili* or handicap*)).ti,hw. (4552)

15 exp *disabilities/ (45529)

16 exp *mental disorders/ (753876)

17 exp *communication disorders/ (53729)

18 exp *physical disorders/ (510075)

19 exp *intellectual development disorder/ or exp *brain damage/ or *cognitive impairment/ (72524)

20 poliomyelitis/ (259)

21 exp amputation/ (1470)

22 exp *paralysis/ or exp *central nervous system disorders/ or *dysarthria/ or exp *spinal cord injuries/ (197900)

23 exp *movement disorders/ (27400)

24 or/1-23 (1315904)

25 developing countries/ or emerging economies/ (6282)

26 (Africa or Asia or Caribbean or West Indies or Middle East or South America or Latin America or Central America).lo,tw. (49922)

27 (Afghanistan or Albania or Algeria or Angola or Argentina or Armenia or Armenian or Azerbaijan or Bangladesh or Benin or Byelarus or Byelorussian or Belarus or Belorussian or Belorussia or Belize or Bhutan or Bolivia or Bosnia or Herzegovina or Hercegovina or Botswana or Brazil or Bulgaria or Burkina Faso or Burkina Fasso or Upper Volta or Burundi or Urundi or Cambodia or Khmer Republic or Kampuchea or Cameroon or Cameroons or Cameron or Camerons or Cape Verde or Central African Republic or Chad or China or Colombia or Comoros or Comoro Islands or Comores or Mayotte or Congo or Zaire or Costa Rica or Cote d'Ivoire or Ivory Coast or Cuba or Djibouti or French Somaliland or Dominica or Dominican Republic or East Timor or East Timur or Timor Leste or Ecuador or Egypt or United Arab Republic or El Salvador or Eritrea or Ethiopia or Fiji or Gabon or Gabonese Republic or Gambia or Gaza or Georgia Republic or Georgian Republic or Ghana or Grenada or Guatemala or Guinea or Guiana or Guyana or Haiti or Honduras or India or Maldives or Indonesia or Iran or Iraq or Jamaica or Jordan or Kazakhstan or Kazakh or Kenya or Kiribati or Korea or Kosovo or Kyrgyzstan or Kirghizia or Kyrgyz Republic or Kirghiz or Kirgizstan or Lao PDR or Laos or Lebanon or Lesotho or Basutoland or Liberia or Libya or Macedonia or Madagascar or Malagasy Republic or Malaysia or Malaya or Malay or Sabah or Sarawak or Malawi or Mali or Marshall Islands or Mauritania or Mauritius or Agalega Islands or Mexico or Micronesia or Middle East or Moldova or Moldovia or Moldovian or Mongolia or Montenegro or Morocco or Ifni or Mozambique or Myanmar or Myanma or Burma or Namibia or Nepal or Netherlands Antilles or Nicaragua or Niger or Nigeria or Muscat or Pakistan or Palau or Palestine or Panama or Paraguay or Peru or Philippines or Philipines or Phillipines or Phillippines or Papua New Guinea or Romania or Rumania or Roumania or Rwanda or Ruanda or Saint Lucia or St Lucia or Saint Vincent or St Vincent or Grenadines or Samoa or Samoan Islands or Navigator Island or Navigator Islands or Sao Tome or Senegal or Serbia or Montenegro or Seychelles or Sierra Leone or Sri Lanka or Solomon Islands or Somalia or Sudan or Suriname or Surinam or Swaziland or South Africa or Syria or Tajikistan or Tadzhikistan or Tadjikistan or Tadzhik or Tanzania or Thailand or Togo or Togolese Republic or Tonga or Tunisia or Turkey or Turkmenistan or Turkmen or Uganda or Ukraine or Uzbekistan or Uzbek or Vanuatu or New Hebrides or Venezuela or Vietnam or Viet Nam or West Bank or Yemen or Zambia or Zimbabwe).lo,tw. (272978)

28 ((developing or less* developed or under developed or underdeveloped or middle income or low* income or underserved or under served or deprived or poor*) adj (countr* or nation? or population? or world or state*)).ti,ab. (17560)

29 ((developing or less* developed or under developed or underdeveloped or middle income or low* income) adj (economy or economies)).ti,ab. (366)

30 (low* adj (gdp or gnp or gross domestic or gross national)).tw. (45)

31 (lmic or lmics or third world or lami countr*).tw. (1862)

32 transitional countr*.tw. (66)

33 or/25-32 (297854)

34 ((systematic* or synthes*) adj3 (research or evaluation* or finding* or thematic* or report or descriptive or explanatory or narrative or meta* or review* or data or literature or studies or evidence or map or quantitative or study or studies or paper or impact or impacts or effect* or compar*)).ti,ab. (62108)

35 ("meta regression" or "meta synth*" or "meta-synth*" or "meta analy*" or "metaanaly*" or "meta-analy*" or "metanaly*" or "metaregression" or "metaregression" or "methodologic* overview" or "pool* analys*" or "pool* data" or "quantitative* overview" or "research integration").ti,ab. (38042)

36 (review adj3 (effectiveness or effects or systemat* or synth* or integrat* or map* or methodologic* or quantitative or evidence or literature)).ti,ab. (87936)

37 ("meta ethnograph*" or "meta synthesis" or (synthesis and ("qualitative literature" or "qualitative research")) or "critical interpretive synthesis" or ("systematic review" and ("qualitative research" or "qualitative literature" or "qualitative stud*")) or "thematic synthesis" or "framework synthesis" or "realist review" or "realist synthesis" or "qualitative systematic review*" or "qualitative evidence synthes*" or (("quality assessment" or "critical appraisal" or "literature search*") and ("qualitative research" or "qualitative literature" or "qualitative stud*")) or (Noblit and Hare) or "meta narrative*" or "narrative synthesis").ti,ab. (3267)

38 experimental design/ or clinical trials/ or cohort analysis/ or followup studies/ or exp longitudinal studies/ or qualitative research/ or quantitative methods/ or quasi experimental methods/ or cohort analysis/ or retrospective studies/ or time series/ or case report/ or meta analysis/ or systematic review/ (91294)

39 or/34-38 (226160)

40 24 and 33 and 39 (4225)

41 limit 40 to yr="2018 -Current" (**624**)

1. **CAB Global Health (Ovid) <1910 to 2020 Week 07>Searched 26^th^ Feb 2020**

1 ((disable* or disabilit* or handicapped) adj5 (person* or people or child* or adolescen* or women or mother* or maternal or group*)).ti. (1594)

2 ((physical* or intellectual* or learning or psychiatric* or sensory or motor or neuromotor or cognitive or mental* or developmental or communication or learning) adj2 (disabilit* or disabl* or handicap*)).ti. (1204)

3 ((cognitive* or learning or mobility or sensory or visual* or vision or sight or hearing or physical* or mental* or intellectual*) adj2 (impair* or disabilit* or disabl* or handicap*)).ti. (3692)

4 ((communication or language or speech or learning) adj5 disorder*).ti. (104)

5 ((depression or depressive or anxiety or psychiat* or well-being or quality of life or self-esteem or self perception) adj2 (impair* or disabilit* or disabl* or handicap*)).ti. (126)

6 mental health.ti. (6986)

7 ((schizophreni* or psychos* or psychotic or schizoaffective or schizophreniform or dementia* or alzheimer*) adj2 (impair* or disabilit* or disabl* or handicap*)).ti. (167)

8 ((mental* or emotional* or psychiatric or neurologic*) adj2 (disorder* or ill or illness*)).ti. (3181)

9 (autis* or dyslexi* or Down* syndrome or mongolism or trisomy 21).ti. (2888)

10 ((intellectual* or educational* or mental* or psychological* or developmental) adj5 (impair* or retard* or deficien* or disable* or disabili* or handicap* or ill*)).ti. (2519)

11 (((hearing or acoustic or ear*) adj5 (loss* or impair* or deficien* or disable* or disabili* or handicap*)) or deaf*).ti. (2517)

12 (((visual* or vision or eye* or ocular) adj5 (loss* or impair* or deficien* or disable* or disabili* or handicap*)) or blind*).ti. (9722)

13 ((cerebral pals* or spina bifida or muscular dystroph* or arthriti* or osteogenesis imperfecta or musculoskeletal abnormalit* or musculo-skeletal abnormalit* or muscular abnormalit* or skeletal abnormalit* or limb abnormalit* or brain injur* or amput* or clubfoot or polio* or paraplegi* or paralys* or paralyz* or hemiplegi* or stroke* or cerebrovascular accident*) adj2 (impair* or disabilit* or disabl* or handicap*)).ti. (32)

14 (physical* adj5 (impair* or deficien* or disable* or disabili* or handicap*)).ti. (561)

15 people with disabilities/ or children with disabilities/ or people with mental disabilities/ or people with physical disabilities/ (4785)

16 abnormalities/ or exp congenital abnormalities/ or exp deformities/ or exp disabilities/ or exp malformations/ (32518)

17 exp mental disorders/ or exp mental health/ or learning disabilities/ or paralysis/ or paraparesis/ or paraplegia/ or poliomyelitis/ or hearing impairment/ or deafness/ or people with hearing impairment/ or vision disorders/ or blindness/ or people with visual impairment/ (103874)

18 or/1-17 (146038)

19 exp africa/ (259467)

20 exp Central America/ or exp Latin America/ or exp South America/ (192441)

21 mexico/ (23681)

22 exp central asia/ (13399)

23 east asia/ or china/ or korea democratic people's republic/ or korea republic/ or mongolia/ (257544)

24 exp south asia/ or himalaya/ (161877)

25 exp south east asia/ or pacific rim/ (104598)

26 exp caribbean/ (22864)

27 exp pacific islands/ (14813)

28 exp developing countries/ (970661)

29 (Africa or Asia or Caribbean or West Indies or South America or Latin America or Central America).tw. (1090170)

30 (Afghanistan or Albania or Algeria or Angola or Argentina or Armenia or Armenian or Azerbaijan or Bangladesh or Benin or Byelarus or Byelorussian or Belarus or Belorussian or Belorussia or Belize or Bhutan or Bolivia or Bosnia or Herzegovina or Hercegovina or Botswana or Brazil or Bulgaria or Burkina Faso or Burkina Fasso or Upper Volta or Burundi or Urundi or Cambodia or Khmer Republic or Kampuchea or Cameroon or Cameroons or Cameron or Camerons or Cape Verde or Central African Republic or Chad or China or Colombia or Comoros or Comoro Islands or Comores or Mayotte or Congo or Zaire or Costa Rica or Cote d'Ivoire or Ivory Coast or Cuba or Djibouti or French Somaliland or Dominica or Dominican Republic or East Timor or East Timur or Timor Leste or Ecuador or Egypt or United Arab Republic or El Salvador or Eritrea or Ethiopia or Fiji or Gabon or Gabonese Republic or Gambia or Gaza or Georgia Republic or Georgian Republic or Ghana or Grenada or Guatemala or Guinea or Guiana or Guyana or Haiti or Honduras or India or Maldives or Indonesia or Iran or Iraq or Jamaica or Jordan or Kazakhstan or Kazakh or Kenya or Kiribati or Korea or Kosovo or Kyrgyzstan or Kirghizia or Kyrgyz Republic or Kirghiz or Kirgizstan or Lao PDR or Laos or Lebanon or Lesotho or Basutoland or Liberia or Libya or Macedonia or Madagascar or Malagasy Republic or Malaysia or Malaya or Malay or Sabah or Sarawak or Malawi or Mali or Marshall Islands or Mauritania or Mauritius or Agalega Islands or Mexico or Micronesia or Middle East or Moldova or Moldovia or Moldovian or Mongolia or Montenegro or Morocco or Ifni or Mozambique or Myanmar or Myanma or Burma or Namibia or Nepal or Netherlands Antilles or Nicaragua or Niger or Nigeria or Muscat or Pakistan or Palau or Palestine or Panama or Paraguay or Peru or Philippines or Philipines or Phillipines or Phillippines or Papua New Guinea or Romania or Rumania or Roumania or Rwanda or Ruanda or Saint Lucia or St Lucia or Saint Vincent or St Vincent or Grenadines or Samoa or Samoan Islands or Navigator Island or Navigator Islands or Sao Tome or Senegal or Serbia or Montenegro or Seychelles or Sierra Leone or Sri Lanka or Solomon Islands or Somalia or Sudan or Suriname or Surinam or Swaziland or South Africa or Syria or Tajikistan or Tadzhikistan or Tadjikistan or Tadzhik or Tanzania or Thailand or Togo or Togolese Republic or Tonga or Tunisia or Turkey or Turkmenistan or Turkmen or Uganda or Ukraine or Uzbekistan or Uzbek or Vanuatu or New Hebrides or Venezuela or Vietnam or Viet Nam or West Bank or Yemen or Zambia or Zimbabwe).tw. (1062515)

31 ((developing or less* developed or under developed or underdeveloped or middle income or low* income or underserved or under served or deprived or poor*) adj (countr* or nation? or population? or world or state*)).ti,ab. (55654)

32 ((developing or less* developed or under developed or underdeveloped or middle income or low* income) adj (economy or economies)).ti,ab. (301)

33 (low* adj (gdp or gnp or gross domestic or gross national)).tw. (73)

34 (low adj3 middle adj3 countr*).tw. (8189)

35 (lmic or lmics or third world or lami countr*).tw. (24530)

36 transitional countr*.tw. (98)

37 or/19-35 (1221707)

38 ((systematic* or synthes*) adj3 (research or evaluation* or finding* or thematic* or report or descriptive or explanatory or narrative or meta* or review* or data or literature or studies or evidence or map or quantitative or study or studies or paper or impact or impacts or effect* or compar*)).ti,ab. (52329)

39 ("meta regression" or "meta synth*" or "meta-synth*" or "meta analy*" or "metaanaly*" or "meta-analy*" or "metanaly*" or "metaregression" or "metaregression" or "methodologic* overview" or "pool* analys*" or "pool* data" or "quantitative* overview" or "research integration").ti,ab. (35021)

40 (review adj3 (effectiveness or effects or systemat* or synth* or integrat* or map* or methodologic* or quantitative or evidence or literature)).ti,ab. (60987)

41 ("meta ethnograph*" or "meta synthesis" or (synthesis and ("qualitative literature" or "qualitative research")) or "critical interpretive synthesis" or ("systematic review" and ("qualitative research" or "qualitative literature" or "qualitative stud*")) or "thematic synthesis" or "framework synthesis" or "realist review" or "realist synthesis" or "qualitative systematic review*" or "qualitative evidence synthes*" or (("quality assessment" or "critical appraisal" or "literature search*") and ("qualitative research" or "qualitative literature" or "qualitative stud*")) or (Noblit and Hare) or "meta narrative*" or "narrative synthesis").ti,ab. (1545)

42 meta-analysis/ or evaluation studies/ or qualitative research/ (26345)

43 controlled clinical trial/ or randomized controlled trial/ or equivalence trial/ or pragmatic clinical trial/ or case-control studies/ or retrospective studies/ or cohort studies/ or follow-up studies/ or longitudinal studies/ or prospective studies/ or epidemiologic methods/ or epidemiologic studies/ or controlled before-after studies/ or cross-sectional studies/ or interrupted time series analysis/ or control groups/ or cross-over studies/ or double-blind method/ or matched-pair analysis/ or meta-analysis as topic/ or random allocation/ or single-blind method/ or "retraction of publication"/ or case reports/ (205801)

44 (random$ or placebo$ or single blind$ or double blind$ or triple blind$ or cohort$ or ((case$ or cohort or follow up or follow-up) adj2 (control$ or series or report$ or study or studies)) or retrospective$ or (observ$ adj3 (study or studies))).ti,ab. (572678)

45 meta-analysis/ or systematic reviews/ (41842)

46 qualitative analysis/ (2128)

47 clinical trials/ or randomized controlled trials/ (57929)

48 case-control studies/ or experimental design/ or retrospective studies/ or longitudinal studies/ or cohort studies/ or follow up/ or time series/ (101230)

49 or/38-48 (715947)

50 18 and 37 and 49 (17493)

51 limit 50 to yr="2018 -Current" (**2912**)

1. **CINAHL (Ebsco) – Searched 26^th^ February 2020**

S41 S19 AND S30 AND S40 Limiters - Published Date: 20180201-20201231; Exclude MEDLINE records

Database - CINAHL Plus with Full Text **1,632**

S40 S31 OR S32 OR S33 OR S34 OR S35 OR S36 OR S37 OR S38 OR S39 Limiters - Published Date: 20180201-20201231

Database - CINAHL Plus with Full Text 143,470

S39 (MH "Random Assignment") Limiters - Published Date: 20180201-20201231

Database - CINAHL Plus with Full Text 7,738

S38 (MH "Crossover Design") OR (MH "Qualitative Studies+") OR (MH "Quasi-Experimental Studies+") OR (MH "Quantitative Studies") OR (MH "Retrospective Design") Limiters - Published Date: 20180201-20201231

Database - CINAHL Plus with Full Text 66,891

S37 (MH "Systematic Review") Limiters - Published Date: 20180201-20201231

Database - CINAHL Plus with Full Text 17,614

S36 (MH "Meta Analysis") Limiters - Published Date: 20180201-20201231

Database - CINAHL Plus with Full Text 9,381

S35 (MH "Controlled Before-After Studies") OR (MH "Interrupted Time Series Analysis") OR (MH "Pretest-Posttest Design") OR (MH "Randomized Controlled Trials") OR (MH "Clinical Trials") OR (MH "Intervention Trials") OR (MH "Double-Blind Studies") OR (MH "Preventive Trials") OR (MH "Community Trials") Limiters - Published Date: 20180201-20201231

Database - CINAHL Plus with Full Text 32,692

S34 TI("meta ethnograph*" or "meta synthesis" or (synthesis and ("qualitative literature" or "qualitative research")) or "critical interpretive synthesis" or ("systematic review" and ("qualitative research" or "qualitative literature" or "qualitative stud*")) or "thematic synthesis" or "framework synthesis" or "realist review" or "realist synthesis" or "qualitative systematic review*" or "qualitative evidence synthes*" or (("quality assessment" or "critical appraisal" or "literature search*") and ("qualitative research" or "qualitative literature" or "qualitative stud*")) or (Noblit and Hare) or "meta narrative*" or "narrative synthesis") OR AB("meta ethnograph*" or "meta synthesis" or (synthesis and ("qualitative literature" or "qualitative research")) or "critical interpretive synthesis" or ("systematic review" and ("qualitative research" or "qualitative literature" or "qualitative stud*")) or "thematic synthesis" or "framework synthesis" or "realist review" or "realist synthesis" or "qualitative systematic review*" or "qualitative evidence synthes*" or (("quality assessment" or "critical appraisal" or "literature search*") and ("qualitative research" or "qualitative literature" or "qualitative stud*")) or (Noblit and Hare) or "meta narrative*" or "narrative synthesis") Limiters - Published Date: 20180201-20201231

Database - CINAHL Plus with Full Text 1,596

S33 TI(review N3 (effectiveness or effects or systemat* or synth* or integrat* or map* or methodologic* or quantitative or evidence or literature)) OR AB(review N3 (effectiveness or effects or systemat* or synth* or integrat* or map* or methodologic* or quantitative or evidence or literature)) Limiters - Published Date: 20180201-20201231

Database - CINAHL Plus with Full Text 37,760

S32 TI("meta regression" or "meta synth*" or "meta-synth*" or "meta analy*" or "metaanaly*" or "meta-analy*" or "metanaly*" or "metaregression" or "metaregression" or "methodologic* overview" or "pool* analys*" or "pool* data" or "quantitative* overview" or "research integration") OR AB("meta regression" or "meta synth*" or "meta-synth*" or "meta analy*" or "metaanaly*" or "meta-analy*" or "metanaly*" or "metaregression" or "metaregression" or "methodologic* overview" or "pool* analys*" or "pool* data" or "quantitative* overview" or "research integration") Limiters - Published Date: 20180201-20201231

Database - CINAHL Plus with Full Text 19,606

S31 TI((systematic* or synthes*) N3 (research or evaluation* or finding* or thematic* or report or descriptive or explanatory or narrative or meta* or review* or data or literature or studies or evidence or map or quantitative or study or studies or paper or impact or impacts or effect* or compar*)) OR AB((systematic* or synthes*) N3 (research or evaluation* or finding* or thematic* or report or descriptive or explanatory or narrative or meta* or review* or data or literature or studies or evidence or map or quantitative or study or studies or paper or impact or impacts or effect* or compar*)) Limiters - Published Date: 20180201-20201231

Database - CINAHL Plus with Full Text 29,586

S30 S20 OR S21 OR S22 OR S23 OR S24 OR S25 OR S26 OR S27 OR S28 OR S29 Limiters - Published Date: 20180201-20201231

Database - CINAHL Plus with Full Text 71,730

S29 TI (Africa or Asia or Caribbean or "West Indies" or "South America" or "Latin America" or "Central America") OR AB (Africa or Asia or Caribbean or "West Indies" or "South America" or "Latin America" or "Central America") OR SU (Africa or Asia or Caribbean or "West Indies" or "South America" or "Latin America" or "Central America") OR GE (Africa or Asia or Caribbean or "West Indies" or "South America" or "Latin America" or "Central America") Limiters - Published Date: 20180201-20201231

Database - CINAHL Plus with Full Text 9,582

S28 TI ( ("transitional country" or "transitional countries") ) OR AB ( ("transitional country" or "transitional countries") ) OR SU ( ("transitional country" or "transitional countries") ) Limiters - Published Date: 20180201-20201231

Database - CINAHL Plus with Full Text 8

S27 TI ( (lmic or lmics or "third world" or "lami country" or "lami countries") ) OR AB ( (lmic or lmics or "third world" or "lami country" or "lami countries") ) OR SU ( (lmic or lmics or "third world" or "lami country" or "lami countries") ) Limiters - Published Date: 20180201-20201231

Database - CINAHL Plus with Full Text 752

S26 TI (low N3 middle N3 countr*) OR AB (low N3 middle N3 countr*) OR SU (low N3 middle N3 countr*) Limiters - Published Date: 20180201-20201231

Database - CINAHL Plus with Full Text 2,772

S25 TI ( low* N1 (gdp or gnp or "gross domestic" or "gross national") ) OR AB ( low* N1 (gdp or gnp or "gross domestic" or "gross national") ) OR SU ( low* N1 (gdp or gnp or "gross domestic" or "gross national") ) Limiters - Published Date: 20180201-20201231

Database - CINAHL Plus with Full Text 11

S24 TI ( (developing or less* N1 developed or "under developed" or underdeveloped or "middle income" or low* N1 income) N1 (economy or economies) ) OR AB ( (developing or less* N1 developed or "under developed" or underdeveloped or "middle income" or low* N1 income) N1 (economy or economies) ) OR SU ( (developing or less* N1 developed or "under developed" or underdeveloped or "middle income" or low* N1 income) N1 (economy or economies) ) Limiters - Published Date: 20180201-20201231

Database - CINAHL Plus with Full Text 29

S23 TI ( (developing or less* N1 developed or "under developed" or underdeveloped or "middle income" or low* N1 income or underserved or "under served" or deprived or poor*) N1 (countr* or nation* or population* or world) ) OR AB ( (developing or less* N1 developed or "under developed" or underdeveloped or "middle income" or low* N1 income or underserved or "under served" or deprived or poor*) N1 (countr* or nation* or population* or world) ) OR SU ( (developing or less* N1 developed or "under developed" or underdeveloped or "middle income" or low* N1 income or underserved or "under served" or deprived or poor*) N1 (countr* or nation* or population* or world) ) Limiters - Published Date: 20180201-20201231

Database - CINAHL Plus with Full Text 6,976

S22 AB Afghanistan OR Albania OR Algeria OR Angola OR Antigua OR Barbuda OR Argentina OR Armenia OR Armenian OR Aruba OR Azerbaijan OR Bahrain OR Bangladesh OR Barbados OR Benin OR Belize OR Bhutan OR Bolivia OR Botswana OR Brazil OR Brasil OR "Burkina Faso" OR "Burkina Fasso" OR "Upper Volta" OR Burundi OR Urundi OR Cambodia OR "Khmer Republic" OR Kampuchea OR Cameroon OR Cameroons OR Cameron OR Camerons OR "Cape Verde" OR "Central African Republic" OR Chad OR Chile OR China OR Colombia OR Comoros OR "Comoro Islands" OR Comores OR Mayotte OR Congo OR Zaire OR "Costa Rica" OR "Cote d'Ivoire" OR "Ivory Coast" OR Cuba OR "Djibouti" OR "French Somaliland" OR Dominica OR "Dominican Republic" OR "East Timor" OR "East Timur" OR "Timor Leste" OR Ecuador OR Egypt OR "United Arab Republic" OR "El Salvador" OR Eritrea OR Ethiopia OR Fiji OR Gabon OR "Gabonese Republic" OR Gambia OR Gaza OR "Georgia Republic" OR "Georgian Republic" OR Ghana OR "Gold Coast" OR Grenada OR Guatemala OR Guinea OR Guam OR Guiana OR Guyana OR Haiti OR Honduras OR India OR Maldives OR Indonesia OR Iran OR Iraq OR Jamaica OR Jordan OR Kazakhstan OR Kazakh OR Kenya OR Kiribati OR Korea OR Kosovo OR Kyrgyzstan OR Kirghizia OR "Kyrgyz Republic" OR Kirghiz OR Kirgizstan OR "Lao PDR" OR Laos OR Lebanon OR Lesotho OR Basutoland OR Liberia OR Libya OR Madagascar OR "Malagasy Republic" OR Malaysia OR Malaya OR Malay OR Sabah OR Sarawak OR Malawi OR Nyasaland OR Mali OR "Marshall Islands" OR Mauritania OR Mauritius OR "Agalega Islands" OR Mexico OR Micronesia OR "Middle East" OR Moldova OR Moldovia OR Moldovian OR Mongolia OR Montenegro OR Morocco OR Ifni OR Mozambique OR Myanmar OR Myanma OR Burma OR Namibia OR Nepal OR Antilles OR "New Caledonia" OR Nicaragua OR Niger OR Nigeria OR "Mariana Islands" OR Oman OR Muscat OR Pakistan OR Palau OR Palestine OR Panama OR Paraguay OR Peru OR Philippines OR Philipines OR Phillipines OR Phillippines OR "Puerto Rico" OR Rwanda OR Ruanda OR "Saint Kitts" OR "St Kitts" OR Nevis OR "Saint Lucia" OR "St Lucia" OR "Saint Vincent" OR "St Vincent" OR "Grenadines" OR "Samoa" OR "Samoan Islands" OR "Navigator Island" OR "Navigator Islands" OR "Sao Tome" OR "Saudi Arabia" OR Senegal OR Seychelles OR "Sierra Leone" OR "Sri Lanka" OR "Solomon Islands" OR Somalia OR Sudan OR Suriname OR Surinam OR Swaziland OR Syria OR Tajikistan OR Tadzhikistan OR Tadjikistan OR Tadzhik OR Tanzania OR Thailand OR Togo OR "Togolese Republic" OR Tonga OR Trinidad OR Tobago OR Tunisia OR Turkey OR Turkmenistan OR Turkmen OR Uganda OR Ukraine OR Uruguay OR Uzbekistan OR Uzbek OR Vanuatu OR "New Hebrides" OR Venezuela OR Vietnam OR "Viet Nam" OR "West Bank" OR Yemen OR Zambia OR Zimbabwe OR Jamahiriya OR Jamahiryria OR Libia OR Mocambique OR Principe OR Syrian OR "Indian Ocean" OR Melanesia OR "Western Sahara" Limiters - Published Date: 20180201-20201231

Database - CINAHL Plus with Full Text 62,549

S21 TI Afghanistan OR Albania OR Algeria OR Angola OR Antigua OR Barbuda OR Argentina OR Armenia OR Armenian OR Aruba OR Azerbaijan OR Bahrain OR Bangladesh OR Barbados OR Benin OR Belize OR Bhutan OR Bolivia OR Botswana OR Brazil OR Brasil OR "Burkina Faso" OR "Burkina Fasso" OR "Upper Volta" OR Burundi OR Urundi OR Cambodia OR "Khmer Republic" OR Kampuchea OR Cameroon OR Cameroons OR Cameron OR Camerons OR "Cape Verde" OR "Central African Republic" OR Chad OR Chile OR China OR Colombia OR Comoros OR "Comoro Islands" OR Comores OR Mayotte OR Congo OR Zaire OR "Costa Rica" OR "Cote d'Ivoire" OR "Ivory Coast" OR Cuba OR "Djibouti" OR "French Somaliland" OR Dominica OR "Dominican Republic" OR "East Timor" OR "East Timur" OR "Timor Leste" OR Ecuador OR Egypt OR "United Arab Republic" OR "El Salvador" OR Eritrea OR Ethiopia OR Fiji OR Gabon OR "Gabonese Republic" OR Gambia OR Gaza OR "Georgia Republic" OR "Georgian Republic" OR Ghana OR "Gold Coast" OR Grenada OR Guatemala OR Guinea OR Guam OR Guiana OR Guyana OR Haiti OR Honduras OR India OR Maldives OR Indonesia OR Iran OR Iraq OR Jamaica OR Jordan OR Kazakhstan OR Kazakh OR Kenya OR Kiribati OR Korea OR Kosovo OR Kyrgyzstan OR Kirghizia OR "Kyrgyz Republic" OR Kirghiz OR Kirgizstan OR "Lao PDR" OR Laos OR Lebanon OR Lesotho OR Basutoland OR Liberia OR Libya OR Madagascar OR "Malagasy Republic" OR Malaysia OR Malaya OR Malay OR Sabah OR Sarawak OR Malawi OR Nyasaland OR Mali OR "Marshall Islands" OR Mauritania OR Mauritius OR "Agalega Islands" OR Mexico OR Micronesia OR "Middle East" OR Moldova OR Moldovia OR Moldovian OR Mongolia OR Montenegro OR Morocco OR Ifni OR Mozambique OR Myanmar OR Myanma OR Burma OR Namibia OR Nepal OR Antilles OR "New Caledonia" OR Nicaragua OR Niger OR Nigeria OR "Mariana Islands" OR Oman OR Muscat OR Pakistan OR Palau OR Palestine OR Panama OR Paraguay OR Peru OR Philippines OR Philipines OR Phillipines OR Phillippines OR "Puerto Rico" OR Rwanda OR Ruanda OR "Saint Kitts" OR "St Kitts" OR Nevis OR "Saint Lucia" OR "St Lucia" OR "Saint Vincent" OR "St Vincent" OR "Grenadines" OR "Samoa" OR "Samoan Islands" OR "Navigator Island" OR "Navigator Islands" OR "Sao Tome" OR "Saudi Arabia" OR Senegal OR Seychelles OR "Sierra Leone" OR "Sri Lanka" OR "Solomon Islands" OR Somalia OR Sudan OR Suriname OR Surinam OR Swaziland OR Syria OR Tajikistan OR Tadzhikistan OR Tadjikistan OR Tadzhik OR Tanzania OR Thailand OR Togo OR "Togolese Republic" OR Tonga OR Trinidad OR Tobago OR Tunisia OR Turkey OR Turkmenistan OR Turkmen OR Uganda OR Ukraine OR Uruguay OR Uzbekistan OR Uzbek OR Vanuatu OR "New Hebrides" OR Venezuela OR Vietnam OR "Viet Nam" OR "West Bank" OR Yemen OR Zambia OR Zimbabwe OR Jamahiriya OR Jamahiryria OR Libia OR Mocambique OR Principe OR Syrian OR "Indian Ocean" OR Melanesia OR "Western Sahara" Limiters - Published Date: 20180201-20201231

Database - CINAHL Plus with Full Text 62,515

S20 SU Afghanistan OR Albania OR Algeria OR Angola OR Antigua OR Barbuda OR Argentina OR Armenia OR Armenian OR Aruba OR Azerbaijan OR Bahrain OR Bangladesh OR Barbados OR Benin OR Belize OR Bhutan OR Bolivia OR Botswana OR Brazil OR Brasil OR "Burkina Faso" OR "Burkina Fasso" OR "Upper Volta" OR Burundi OR Urundi OR Cambodia OR "Khmer Republic" OR Kampuchea OR Cameroon OR Cameroons OR Cameron OR Camerons OR "Cape Verde" OR "Central African Republic" OR Chad OR Chile OR China OR Colombia OR Comoros OR "Comoro Islands" OR Comores OR Mayotte OR Congo OR Zaire OR "Costa Rica" OR "Cote d'Ivoire" OR "Ivory Coast" OR Cuba OR "Djibouti" OR "French Somaliland" OR Dominica OR "Dominican Republic" OR "East Timor" OR "East Timur" OR "Timor Leste" OR Ecuador OR Egypt OR "United Arab Republic" OR "El Salvador" OR Eritrea OR Ethiopia OR Fiji OR Gabon OR "Gabonese Republic" OR Gambia OR Gaza OR "Georgia Republic" OR "Georgian Republic" OR Ghana OR "Gold Coast" OR Grenada OR Guatemala OR Guinea OR Guam OR Guiana OR Guyana OR Haiti OR Honduras OR India OR Maldives OR Indonesia OR Iran OR Iraq OR Jamaica OR Jordan OR Kazakhstan OR Kazakh OR Kenya OR Kiribati OR Korea OR Kosovo OR Kyrgyzstan OR Kirghizia OR "Kyrgyz Republic" OR Kirghiz OR Kirgizstan OR "Lao PDR" OR Laos OR Lebanon OR Lesotho OR Basutoland OR Liberia OR Libya OR Madagascar OR "Malagasy Republic" OR Malaysia OR Malaya OR Malay OR Sabah OR Sarawak OR Malawi OR Nyasaland OR Mali OR "Marshall Islands" OR Mauritania OR Mauritius OR "Agalega Islands" OR Mexico OR Micronesia OR "Middle East" OR Moldova OR Moldovia OR Moldovian OR Mongolia OR Montenegro OR Morocco OR Ifni OR Mozambique OR Myanmar OR Myanma OR Burma OR Namibia OR Nepal OR Antilles OR "New Caledonia" OR Nicaragua OR Niger OR Nigeria OR "Mariana Islands" OR Oman OR Muscat OR Pakistan OR Palau OR Palestine OR Panama OR Paraguay OR Peru OR Philippines OR Philipines OR Phillipines OR Phillippines OR "Puerto Rico" OR Rwanda OR Ruanda OR "Saint Kitts" OR "St Kitts" OR Nevis OR "Saint Lucia" OR "St Lucia" OR "Saint Vincent" OR "St Vincent" OR "Grenadines" OR "Samoa" OR "Samoan Islands" OR "Navigator Island" OR "Navigator Islands" OR "Sao Tome" OR "Saudi Arabia" OR Senegal OR Seychelles OR "Sierra Leone" OR "Sri Lanka" OR "Solomon Islands" OR Somalia OR Sudan OR Suriname OR Surinam OR Swaziland OR Syria OR Tajikistan OR Tadzhikistan OR Tadjikistan OR Tadzhik OR Tanzania OR Thailand OR Togo OR "Togolese Republic" OR Tonga OR Trinidad OR Tobago OR Tunisia OR Turkey OR Turkmenistan OR Turkmen OR Uganda OR Ukraine OR Uruguay OR Uzbekistan OR Uzbek OR Vanuatu OR "New Hebrides" OR Venezuela OR Vietnam OR "Viet Nam" OR "West Bank" OR Yemen OR Zambia OR Zimbabwe OR Jamahiriya OR Jamahiryria OR Libia OR Mocambique OR Principe OR Syrian OR "Indian Ocean" OR Melanesia OR "Western Sahara" Limiters - Published Date: 20180201-20201231

Database - CINAHL Plus with Full Text 62,544

S19 S1 OR S2 OR S3 OR S4 OR S5 OR S6 OR S7 OR S8 OR S9 OR S10 OR S11 OR S12 OR S13 OR S14 OR S15 OR S16 OR S17 OR S18 Limiters - Published Date: 20180201-20201231

Database - CINAHL Plus with Full Text 71,585

S18 MM "Disabled+") OR (MM "Child, Disabled") OR (MM "Health Services for Persons with Disabilities") OR (MM "Mentally Disabled Persons") Limiters - Published Date: 20180201-20201231

Database - CINAHL Plus with Full Text 3,976

S17 (MM "Vision Disorders") OR (MM "Blindness+") OR (MH "Vision, Subnormal/ED/EP/PC/TH/RH") Limiters - Published Date: 20180201-20201231

Database - CINAHL Plus with Full Text 1,088

S16 (MM "Hearing Disorders+/ED/EP/PC/RH/TH") Limiters - Published Date: 20180201-20201231

Database - CINAHL Plus with Full Text 786

S15 (MM "Mental Disorders+/EP/ED/PC/RH/TH") Limiters - Published Date: 20180201-20201231

Database - CINAHL Plus with Full Text 13,979

S14 TI(physical* N5 (impair* or deficien* or disable* or disabili* or handicap*)) OR AB(physical* N5 (impair* or deficien* or disable* or disabili* or handicap*)) Limiters - Published Date: 20180201-20201231

Database - CINAHL Plus with Full Text 1,736

S13 TI(("cerebral pals*" or "spina bifida" or "muscular dystroph*" or arthriti* or "osteogenesis imperfecta" or "musculoskeletal abnormalit*" or "musculo-skeletal abnormalit*" or "muscular abnormalit*" or "skeletal abnormalit*" or "limb abnormalit*" or "brain injur*" or amput* or clubfoot or polio* or paraplegi* or paralys* or paralyz* or hemiplegi* or stroke* or "cerebrovascular accident*") N2 (impair* or disabilit* or disabl* or handicap*)) OR AB(("cerebral pals*" or "spina bifida" or "muscular dystroph*" or arthriti* or "osteogenesis imperfecta" or "musculoskeletal abnormalit*" or "musculo-skeletal abnormalit*" or "muscular abnormalit*" or "skeletal abnormalit*" or "limb abnormalit*" or "brain injur*" or amput* or clubfoot or polio* or paraplegi* or paralys* or paralyz* or hemiplegi* or stroke* or "cerebrovascular accident*") N2 (impair* or disabilit* or disabl* or handicap*)) Limiters - Published Date: 20180201-20201231

Database - CINAHL Plus with Full Text 583

S12 TI(((visual* or vision or eye* or ocular) N5 (loss* or impair* or deficien* or disable* or disabili* or handicap*)) or blind*) OR AB(((visual* or vision or eye* or ocular) N5 (loss* or impair* or deficien* or disable* or disabili* or handicap*)) or blind*) Limiters - Published Date: 20180201-20201231

Database - CINAHL Plus with Full Text 14,417

S11 TI(((hearing or acoustic or ear*) N5 (loss* or impair* or deficien* or disable* or disabili* or handicap*)) or deaf*) OR AB(((hearing or acoustic or ear*) N5 (loss* or impair* or deficien* or disable* or disabili* or handicap*)) or deaf*) Limiters - Published Date: 20180201-20201231

Database - CINAHL Plus with Full Text 5,154

S10 TI((intellectual* or educational* or mental* or psychological* or developmental) N5 (impair* or retard* or deficien* or disable* or disabili* or handicap* or ill*)) OR AB((intellectual* or educational* or mental* or psychological* or developmental) N5 (impair* or retard* or deficien* or disable* or disabili* or handicap* or ill*)) Limiters - Published Date: 20180201-20201231

Database - CINAHL Plus with Full Text 7,736

S9 TI(autis* or dyslexi* or "Down* syndrome" or mongolism or "trisomy 21") OR AB(autis* or dyslexi* or "Down* syndrome" or mongolism or "trisomy 21") Limiters - Published Date: 20180201-20201231

Database - CINAHL Plus with Full Text 5,411

S8 TI((mental* or emotional* or psychiatric or neurologic*) N2 (disorder* or ill or illness*)) OR AB((mental* or emotional* or psychiatric or neurologic*) N2 (disorder* or ill or illness*)) Limiters - Published Date: 20180201-20201231

Database - CINAHL Plus with Full Text 9,379

S7 TI((schizophreni* or psychos* or psychotic or schizoaffective or schizophreniform or dementia* or alzheimer*) N2 (impair* or disabilit* or disabl* or handicap*)) OR AB((schizophreni* or psychos* or psychotic or schizoaffective or schizophreniform or dementia* or alzheimer*) N2 (impair* or disabilit* or disabl* or handicap*)) Limiters - Published Date: 20180201-20201231

Database - CINAHL Plus with Full Text 1,140

S6 TI("mental health") OR AB("mental health") Limiters - Published Date: 20180201-20201231

Database - CINAHL Plus with Full Text 17,746

S5 TI((depression or depressive or anxiety or psychiat* or well-being or "quality of life" or self-esteem or "self perception") N2 (impair* or disabilit* or disabl* or handicap*)) OR AB((depression or depressive or anxiety or psychiat* or well-being or "quality of life" or self-esteem or "self perception") N2 (impair* or disabilit* or disabl* or handicap*) Limiters - Published Date: 20180201-20201231

Database - CINAHL Plus with Full Text 1,511

S4 TI((communication or language or speech or learning) N5 disorder*) OR AB((communication or language or speech or learning) N5 disorder*) Limiters - Published Date: 20180201-20201231

Database - CINAHL Plus with Full Text 1,107

S3 TI((cognitive* or learning or mobility or sensory or visual* or vision or sight or hearing or physical* or mental* or intellectual*) N2 (impair* or disabilit* or disabl* or handicap*)) OR AB((cognitive* or learning or mobility or sensory or visual* or vision or sight or hearing or physical* or mental* or intellectual*) N2 (impair* or disabilit* or disabl* or handicap*)) Limiters - Published Date: 20180201-20201231

Database - CINAHL Plus with Full Text 11,556

S2 TI((physical* or intellectual* or learning or psychiatric* or sensory or motor or neuromotor or cognitive or mental* or developmental or communication or learning) N2 (disabilit* or disabl* or handicap*)) OR AB ((physical* or intellectual* or learning or psychiatric* or sensory or motor or neuromotor or cognitive or mental* or developmental or communication or learning) N2 (disabilit* or disabl* or handicap*)) Limiters - Published Date: 20180201-20201231

Database - CINAHL Plus with Full Text 4,472

S1 TI((disable* or disabilit* or handicapped) N5 (person* or people or child* or adolescen* or women or mother* or maternal or group*)) OR AB ((disable* or disabilit* or handicapped) N5 (person* or people or child* or adolescen* or women or mother* or maternal or group*)) Limiters - Published Date: 20180201-20201231

Database - CINAHL Plus with Full Text 4,528

1. **ERIC (Ebsco) – Searched 26^th^ February 2020**

S34 S16 AND S27 AND S33

Database - ERIC 83

S33 S28 OR S29 OR S30 OR S31 OR S32 Limiters - Date Published: 20180201-20201231

Database - ERIC 5,619

S32 (DE "Meta Analysis" OR DE "Multiple Regression Analysis" OR DE "Mixed Methods Research" OR DE "Randomized Controlled Trials" OR DE "Evaluation Methods" OR DE "Qualitative Research" OR DE "Quasiexperimental Design" OR DE "Control Groups" OR DE "Matched Groups" OR DE "Pretests Posttests") Limiters - Date Published: 20180201-20201231

Database - ERIC 3,695

S31 TI("meta ethnograph*" or "meta synthesis" or (synthesis and ("qualitative literature" or "qualitative research")) or "critical interpretive synthesis" or ("systematic review" and ("qualitative research" or "qualitative literature" or "qualitative stud*")) or "thematic synthesis" or "framework synthesis" or "realist review" or "realist synthesis" or "qualitative systematic review*" or "qualitative evidence synthes*" or (("quality assessment" or "critical appraisal" or "literature search*") and ("qualitative research" or "qualitative literature" or "qualitative stud*")) or (Noblit and Hare) or "meta narrative*" or "narrative synthesis") OR AB("meta ethnograph*" or "meta synthesis" or (synthesis and ("qualitative literature" or "qualitative research")) or "critical interpretive synthesis" or ("systematic review" and ("qualitative research" or "qualitative literature" or "qualitative stud*")) or "thematic synthesis" or "framework synthesis" or "realist review" or "realist synthesis" or "qualitative systematic review*" or "qualitative evidence synthes*" or (("quality assessment" or "critical appraisal" or "literature search*") and ("qualitative research" or "qualitative literature" or "qualitative stud*")) or (Noblit and Hare) or "meta narrative*" or "narrative synthesis") Limiters - Date Published: 20180201-20201231

Database - ERIC 67

S30 TI(review N3 (effectiveness or effects or systemat* or synth* or integrat* or map* or methodologic* or quantitative or evidence or literature)) OR AB(review N3 (effectiveness or effects or systemat* or synth* or integrat* or map* or methodologic* or quantitative or evidence or literature)) Limiters - Date Published: 20180201-20201231

Database - ERIC 1,487

S29 TI("meta regression" or "meta synth*" or "meta-synth*" or "meta analy*" or "metaanaly*" or "meta-analy*" or "metanaly*" or "metaregression" or "metaregression" or "methodologic* overview" or "pool* analys*" or "pool* data" or "quantitative* overview" or "research integration") OR AB("meta regression" or "meta synth*" or "meta-synth*" or "meta analy*" or "metaanaly*" or "meta-analy*" or "metanaly*" or "metaregression" or "metaregression" or "methodologic* overview" or "pool* analys*" or "pool* data" or "quantitative* overview" or "research integration") Limiters - Date Published: 20180201-20201231

Database - ERIC 396

S28 TI((systematic* or synthes*) N3 (research or evaluation* or finding* or thematic* or report or descriptive or explanatory or narrative or meta* or review* or data or literature or studies or evidence or map or quantitative or study or studies or paper or impact or impacts or effect* or compar*)) OR AB((systematic* or synthes*) N3 (research or evaluation* or finding* or thematic* or report or descriptive or explanatory or narrative or meta* or review* or data or literature or studies or evidence or map or quantitative or study or studies or paper or impact or impacts or effect* or compar*)) Limiters - Date Published: 20180201-20201231

Database - ERIC 1,205

S27 S17 OR S18 OR S19 OR S20 OR S21 OR S22 OR S23 OR S24 OR S25 OR S26 Limiters - Date Published: 20180201-20201231

Database - ERIC 10,382

S26 TI (Africa or Asia or Caribbean or "West Indies" or "South America" or "Latin America" or "Central America") OR AB (Africa or Asia or Caribbean or "West Indies" or "South America" or "Latin America" or "Central America") OR SU (Africa or Asia or Caribbean or "West Indies" or "South America" or "Latin America" or "Central America") OR GE (Africa or Asia or Caribbean or "West Indies" or "South America" or "Latin America" or "Central America") Limiters - Date Published: 20180201-20201231

Database - ERIC 1,074

S25 TI ( ("transitional country" or "transitional countries") ) OR AB ( ("transitional country" or "transitional countries") ) OR SU ( ("transitional country" or "transitional countries") ) Limiters - Date Published: 20180201-20201231

Database - ERIC 2

S24 TI ( (lmic or lmics or "third world" or "lami country" or "lami countries") ) OR AB ( (lmic or lmics or "third world" or "lami country" or "lami countries") ) OR SU ( (lmic or lmics or "third world" or "lami country" or "lami countries") ) Limiters - Date Published: 20180201-20201231

Database - ERIC 18

S23 TI (low N3 middle N3 countr*) OR AB (low N3 middle N3 countr*) OR SU (low N3 middle N3 countr*) Limiters - Date Published: 20180201-20201231

Database - ERIC 52

S22 TI ( low* N1 (gdp or gnp or "gross domestic" or "gross national") ) OR AB ( low* N1 (gdp or gnp or "gross domestic" or "gross national") ) OR SU ( low* N1 (gdp or gnp or "gross domestic" or "gross national") ) Limiters - Date Published: 20180201-20201231

Database - ERIC 2

S21 TI ( (developing or less* N1 developed or "under developed" or underdeveloped or "middle income" or low* N1 income) N1 (economy or economies) ) OR AB ( (developing or less* N1 developed or "under developed" or underdeveloped or "middle income" or low* N1 income) N1 (economy or economies) ) OR SU ( (developing or less* N1 developed or "under developed" or underdeveloped or "middle income" or low* N1 income) N1 (economy or economies) ) Limiters - Date Published: 20180201-20201231

Database - ERIC 12

S20 TI ( (developing or less* N1 developed or "under developed" or underdeveloped or "middle income" or low* N1 income or underserved or "under served" or deprived or poor*) N1 (countr* or nation* or population* or world) ) OR AB ( (developing or less* N1 developed or "under developed" or underdeveloped or "middle income" or low* N1 income or underserved or "under served" or deprived or poor*) N1 (countr* or nation* or population* or world) ) OR SU ( (developing or less* N1 developed or "under developed" or underdeveloped or "middle income" or low* N1 income or underserved or "under served" or deprived or poor*) N1 (countr* or nation* or population* or world) ) Limiters - Date Published: 20180201-20201231

Database - ERIC 447

S19 AB Afghanistan OR Albania OR Algeria OR Angola OR Antigua OR Barbuda OR Argentina OR Armenia OR Armenian OR Aruba OR Azerbaijan OR Bahrain OR Bangladesh OR Barbados OR Benin OR Belize OR Bhutan OR Bolivia OR Botswana OR Brazil OR Brasil OR "Burkina Faso" OR "Burkina Fasso" OR "Upper Volta" OR Burundi OR Urundi OR Cambodia OR "Khmer Republic" OR Kampuchea OR Cameroon OR Cameroons OR Cameron OR Camerons OR "Cape Verde" OR "Central African Republic" OR Chad OR Chile OR China OR Colombia OR Comoros OR "Comoro Islands" OR Comores OR Mayotte OR Congo OR Zaire OR "Costa Rica" OR "Cote d'Ivoire" OR "Ivory Coast" OR Cuba OR "Djibouti" OR "French Somaliland" OR Dominica OR "Dominican Republic" OR "East Timor" OR "East Timur" OR "Timor Leste" OR Ecuador OR Egypt OR "United Arab Republic" OR "El Salvador" OR Eritrea OR Ethiopia OR Fiji OR Gabon OR "Gabonese Republic" OR Gambia OR Gaza OR "Georgia Republic" OR "Georgian Republic" OR Ghana OR "Gold Coast" OR Grenada OR Guatemala OR Guinea OR Guam OR Guiana OR Guyana OR Haiti OR Honduras OR India OR Maldives OR Indonesia OR Iran OR Iraq OR Jamaica OR Jordan OR Kazakhstan OR Kazakh OR Kenya OR Kiribati OR Korea OR Kosovo OR Kyrgyzstan OR Kirghizia OR "Kyrgyz Republic" OR Kirghiz OR Kirgizstan OR "Lao PDR" OR Laos OR Lebanon OR Lesotho OR Basutoland OR Liberia OR Libya OR Madagascar OR "Malagasy Republic" OR Malaysia OR Malaya OR Malay OR Sabah OR Sarawak OR Malawi OR Nyasaland OR Mali OR "Marshall Islands" OR Mauritania OR Mauritius OR "Agalega Islands" OR Mexico OR Micronesia OR "Middle East" OR Moldova OR Moldovia OR Moldovian OR Mongolia OR Montenegro OR Morocco OR Ifni OR Mozambique OR Myanmar OR Myanma OR Burma OR Namibia OR Nepal OR Antilles OR "New Caledonia" OR Nicaragua OR Niger OR Nigeria OR "Mariana Islands" OR Oman OR Muscat OR Pakistan OR Palau OR Palestine OR Panama OR Paraguay OR Peru OR Philippines OR Philipines OR Phillipines OR Phillippines OR "Puerto Rico" OR Rwanda OR Ruanda OR "Saint Kitts" OR "St Kitts" OR Nevis OR "Saint Lucia" OR "St Lucia" OR "Saint Vincent" OR "St Vincent" OR "Grenadines" OR "Samoa" OR "Samoan Islands" OR "Navigator Island" OR "Navigator Islands" OR "Sao Tome" OR "Saudi Arabia" OR Senegal OR Seychelles OR "Sierra Leone" OR "Sri Lanka" OR "Solomon Islands" OR Somalia OR Sudan OR Suriname OR Surinam OR Swaziland OR Syria OR Tajikistan OR Tadzhikistan OR Tadjikistan OR Tadzhik OR Tanzania OR Thailand OR Togo OR "Togolese Republic" OR Tonga OR Trinidad OR Tobago OR Tunisia OR Turkey OR Turkmenistan OR Turkmen OR Uganda OR Ukraine OR Uruguay OR Uzbekistan OR Uzbek OR Vanuatu OR "New Hebrides" OR Venezuela OR Vietnam OR "Viet Nam" OR "West Bank" OR Yemen OR Zambia OR Zimbabwe OR Jamahiriya OR Jamahiryria OR Libia OR Mocambique OR Principe OR Syrian OR "Indian Ocean" OR Melanesia OR "Western Sahara" Limiters - Date Published: 20180201-20201231

Database - ERIC 9,570

S18 TI Afghanistan OR Albania OR Algeria OR Angola OR Antigua OR Barbuda OR Argentina OR Armenia OR Armenian OR Aruba OR Azerbaijan OR Bahrain OR Bangladesh OR Barbados OR Benin OR Belize OR Bhutan OR Bolivia OR Botswana OR Brazil OR Brasil OR "Burkina Faso" OR "Burkina Fasso" OR "Upper Volta" OR Burundi OR Urundi OR Cambodia OR "Khmer Republic" OR Kampuchea OR Cameroon OR Cameroons OR Cameron OR Camerons OR "Cape Verde" OR "Central African Republic" OR Chad OR Chile OR China OR Colombia OR Comoros OR "Comoro Islands" OR Comores OR Mayotte OR Congo OR Zaire OR "Costa Rica" OR "Cote d'Ivoire" OR "Ivory Coast" OR Cuba OR "Djibouti" OR "French Somaliland" OR Dominica OR "Dominican Republic" OR "East Timor" OR "East Timur" OR "Timor Leste" OR Ecuador OR Egypt OR "United Arab Republic" OR "El Salvador" OR Eritrea OR Ethiopia OR Fiji OR Gabon OR "Gabonese Republic" OR Gambia OR Gaza OR "Georgia Republic" OR "Georgian Republic" OR Ghana OR "Gold Coast" OR Grenada OR Guatemala OR Guinea OR Guam OR Guiana OR Guyana OR Haiti OR Honduras OR India OR Maldives OR Indonesia OR Iran OR Iraq OR Jamaica OR Jordan OR Kazakhstan OR Kazakh OR Kenya OR Kiribati OR Korea OR Kosovo OR Kyrgyzstan OR Kirghizia OR "Kyrgyz Republic" OR Kirghiz OR Kirgizstan OR "Lao PDR" OR Laos OR Lebanon OR Lesotho OR Basutoland OR Liberia OR Libya OR Madagascar OR "Malagasy Republic" OR Malaysia OR Malaya OR Malay OR Sabah OR Sarawak OR Malawi OR Nyasaland OR Mali OR "Marshall Islands" OR Mauritania OR Mauritius OR "Agalega Islands" OR Mexico OR Micronesia OR "Middle East" OR Moldova OR Moldovia OR Moldovian OR Mongolia OR Montenegro OR Morocco OR Ifni OR Mozambique OR Myanmar OR Myanma OR Burma OR Namibia OR Nepal OR Antilles OR "New Caledonia" OR Nicaragua OR Niger OR Nigeria OR "Mariana Islands" OR Oman OR Muscat OR Pakistan OR Palau OR Palestine OR Panama OR Paraguay OR Peru OR Philippines OR Philipines OR Phillipines OR Phillippines OR "Puerto Rico" OR Rwanda OR Ruanda OR "Saint Kitts" OR "St Kitts" OR Nevis OR "Saint Lucia" OR "St Lucia" OR "Saint Vincent" OR "St Vincent" OR "Grenadines" OR "Samoa" OR "Samoan Islands" OR "Navigator Island" OR "Navigator Islands" OR "Sao Tome" OR "Saudi Arabia" OR Senegal OR Seychelles OR "Sierra Leone" OR "Sri Lanka" OR "Solomon Islands" OR Somalia OR Sudan OR Suriname OR Surinam OR Swaziland OR Syria OR Tajikistan OR Tadzhikistan OR Tadjikistan OR Tadzhik OR Tanzania OR Thailand OR Togo OR "Togolese Republic" OR Tonga OR Trinidad OR Tobago OR Tunisia OR Turkey OR Turkmenistan OR Turkmen OR Uganda OR Ukraine OR Uruguay OR Uzbekistan OR Uzbek OR Vanuatu OR "New Hebrides" OR Venezuela OR Vietnam OR "Viet Nam" OR "West Bank" OR Yemen OR Zambia OR Zimbabwe OR Jamahiriya OR Jamahiryria OR Libia OR Mocambique OR Principe OR Syrian OR "Indian Ocean" OR Melanesia OR "Western Sahara" Limiters - Date Published: 20180201-20201231

Database - ERIC 9,569

S17 SU Afghanistan OR Albania OR Algeria OR Angola OR Antigua OR Barbuda OR Argentina OR Armenia OR Armenian OR Aruba OR Azerbaijan OR Bahrain OR Bangladesh OR Barbados OR Benin OR Belize OR Bhutan OR Bolivia OR Botswana OR Brazil OR Brasil OR "Burkina Faso" OR "Burkina Fasso" OR "Upper Volta" OR Burundi OR Urundi OR Cambodia OR "Khmer Republic" OR Kampuchea OR Cameroon OR Cameroons OR Cameron OR Camerons OR "Cape Verde" OR "Central African Republic" OR Chad OR Chile OR China OR Colombia OR Comoros OR "Comoro Islands" OR Comores OR Mayotte OR Congo OR Zaire OR "Costa Rica" OR "Cote d'Ivoire" OR "Ivory Coast" OR Cuba OR "Djibouti" OR "French Somaliland" OR Dominica OR "Dominican Republic" OR "East Timor" OR "East Timur" OR "Timor Leste" OR Ecuador OR Egypt OR "United Arab Republic" OR "El Salvador" OR Eritrea OR Ethiopia OR Fiji OR Gabon OR "Gabonese Republic" OR Gambia OR Gaza OR "Georgia Republic" OR "Georgian Republic" OR Ghana OR "Gold Coast" OR Grenada OR Guatemala OR Guinea OR Guam OR Guiana OR Guyana OR Haiti OR Honduras OR India OR Maldives OR Indonesia OR Iran OR Iraq OR Jamaica OR Jordan OR Kazakhstan OR Kazakh OR Kenya OR Kiribati OR Korea OR Kosovo OR Kyrgyzstan OR Kirghizia OR "Kyrgyz Republic" OR Kirghiz OR Kirgizstan OR "Lao PDR" OR Laos OR Lebanon OR Lesotho OR Basutoland OR Liberia OR Libya OR Madagascar OR "Malagasy Republic" OR Malaysia OR Malaya OR Malay OR Sabah OR Sarawak OR Malawi OR Nyasaland OR Mali OR "Marshall Islands" OR Mauritania OR Mauritius OR "Agalega Islands" OR Mexico OR Micronesia OR "Middle East" OR Moldova OR Moldovia OR Moldovian OR Mongolia OR Montenegro OR Morocco OR Ifni OR Mozambique OR Myanmar OR Myanma OR Burma OR Namibia OR Nepal OR Antilles OR "New Caledonia" OR Nicaragua OR Niger OR Nigeria OR "Mariana Islands" OR Oman OR Muscat OR Pakistan OR Palau OR Palestine OR Panama OR Paraguay OR Peru OR Philippines OR Philipines OR Phillipines OR Phillippines OR "Puerto Rico" OR Rwanda OR Ruanda OR "Saint Kitts" OR "St Kitts" OR Nevis OR "Saint Lucia" OR "St Lucia" OR "Saint Vincent" OR "St Vincent" OR "Grenadines" OR "Samoa" OR "Samoan Islands" OR "Navigator Island" OR "Navigator Islands" OR "Sao Tome" OR "Saudi Arabia" OR Senegal OR Seychelles OR "Sierra Leone" OR "Sri Lanka" OR "Solomon Islands" OR Somalia OR Sudan OR Suriname OR Surinam OR Swaziland OR Syria OR Tajikistan OR Tadzhikistan OR Tadjikistan OR Tadzhik OR Tanzania OR Thailand OR Togo OR "Togolese Republic" OR Tonga OR Trinidad OR Tobago OR Tunisia OR Turkey OR Turkmenistan OR Turkmen OR Uganda OR Ukraine OR Uruguay OR Uzbekistan OR Uzbek OR Vanuatu OR "New Hebrides" OR Venezuela OR Vietnam OR "Viet Nam" OR "West Bank" OR Yemen OR Zambia OR Zimbabwe OR Jamahiriya OR Jamahiryria OR Libia OR Mocambique OR Principe OR Syrian OR "Indian Ocean" OR Melanesia OR "Western Sahara" Limiters - Date Published: 20180201-20201231

Database - ERIC 9,572

S16 S1 OR S2 OR S3 OR S4 OR S5 OR S6 OR S7 OR S8 OR S9 OR S10 OR S11 OR S12 OR S13 OR S14 OR S15 Limiters - Date Published: 20180201-20201231

Database - ERIC 5,429

S15 (DE "Intellectual Disability" OR DE "Down Syndrome" OR DE "Mild Intellectual Disability" OR DE "Moderate Intellectual Disability" OR DE "Severe Intellectual Disability" OR DE "Severe Intellectual Disability" OR DE "Moderate Intellectual Disability" OR DE "Mild Intellectual Disability" OR DE "Severity (of Disability)" OR DE "Disability Identification" OR DE "Disability Discrimination" OR DE "Developmental Disabilities" OR DE "Learning Disabilities" OR DE "Mental Health" OR DE "Mental Disorders" OR DE "Anxiety Disorders" OR DE "Dementia" OR DE "Emotional Disturbances" OR DE "Neurosis" OR DE "Pervasive Developmental Disorders" OR DE "Psychosis" OR DE "Mental Health Programs" OR DE "Disability Identification" OR DE "Deafness" OR DE "Deaf Blind" OR DE "Autism" OR DE "Asperger Syndrome" OR DE "Partial Vision" OR DE "Blindness" OR DE "Depression (Psychology)" OR DE "Speech Impairments" OR DE "Articulation Impairments" OR DE "Delayed Speech" OR DE "Stuttering" OR DE "Voice Disorders" OR DE "Stuttering") Limiters - Date Published: 20180201-20201231

Database - ERIC 4,343

S14 TI(physical* N5 (impair* or deficien* or disable* or disabili* or handicap*)) OR AB(physical* N5 (impair* or deficien* or disable* or disabili* or handicap*)) Limiters - Date Published: 20180201-20201231

Database - ERIC 91

S13 TI(("cerebral pals*" or "spina bifida" or "muscular dystroph*" or arthriti* or "osteogenesis imperfecta" or "musculoskeletal abnormalit*" or "musculo-skeletal abnormalit*" or "muscular abnormalit*" or "skeletal abnormalit*" or "limb abnormalit*" or "brain injur*" or amput* or clubfoot or polio* or paraplegi* or paralys* or paralyz* or hemiplegi* or stroke* or "cerebrovascular accident*") N2 (impair* or disabilit* or disabl* or handicap*)) OR AB(("cerebral pals*" or "spina bifida" or "muscular dystroph*" or arthriti* or "osteogenesis imperfecta" or "musculoskeletal abnormalit*" or "musculo-skeletal abnormalit*" or "muscular abnormalit*" or "skeletal abnormalit*" or "limb abnormalit*" or "brain injur*" or amput* or clubfoot or polio* or paraplegi* or paralys* or paralyz* or hemiplegi* or stroke* or "cerebrovascular accident*") N2 (impair* or disabilit* or disabl* or handicap*)) Limiters - Date Published: 20180201-20201231

Database - ERIC 7

S12 TI(((visual* or vision or eye* or ocular) N5 (loss* or impair* or deficien* or disable* or disabili* or handicap*)) or blind*) OR AB(((visual* or vision or eye* or ocular) N5 (loss* or impair* or deficien* or disable* or disabili* or handicap*)) or blind*) Limiters - Date Published: 20180201-20201231

Database - ERIC 261

S11 TI(((hearing or acoustic or ear*) N5 (loss* or impair* or deficien* or disable* or disabili* or handicap*)) or deaf*) OR AB(((hearing or acoustic or ear*) N5 (loss* or impair* or deficien* or disable* or disabili* or handicap*)) or deaf*) Limiters - Date Published: 20180201-20201231

Database - ERIC 366

S10 TI((intellectual* or educational* or mental* or psychological* or developmental) N5 (impair* or retard* or deficien* or disable* or disabili* or handicap* or ill*)) OR AB((intellectual* or educational* or mental* or psychological* or developmental) N5 (impair* or retard* or deficien* or disable* or disabili* or handicap* or ill*)) Limiters - Date Published: 20180201-20201231

Database - ERIC 1,013

S9 TI(autis* or dyslexi* or "Down* syndrome" or mongolism or "trisomy 21") OR AB(autis* or dyslexi* or "Down* syndrome" or mongolism or "trisomy 21") Limiters - Date Published: 20180201-20201231

Database - ERIC 1,766

S8 TI((mental* or emotional* or psychiatric or neurologic*) N2 (disorder* or ill or illness*)) OR AB((mental* or emotional* or psychiatric or neurologic*) N2 (disorder* or ill or illness*)) Limiters - Date Published: 20180201-20201231

Database - ERIC 254

S7 TI((schizophreni* or psychos* or psychotic or schizoaffective or schizophreniform or dementia* or alzheimer*) N2 (impair* or disabilit* or disabl* or handicap*)) OR AB((schizophreni* or psychos* or psychotic or schizoaffective or schizophreniform or dementia* or alzheimer*) N2 (impair* or disabilit* or disabl* or handicap*)) Limiters - Date Published: 20180201-20201231

Database - ERIC 5

S6 TI("mental health") OR AB("mental health") Limiters - Date Published: 20180201-20201231

Database - ERIC 721

S5 TI((depression or depressive or anxiety or psychiat* or well-being or "quality of life" or self-esteem or "self perception") N2 (impair* or disabilit* or disabl* or handicap*)) OR AB((depression or depressive or anxiety or psychiat* or well-being or "quality of life" or self-esteem or "self perception") N2 (impair* or disabilit* or disabl* or handicap*) Limiters - Date Published: 20180201-20201231

Database - ERIC 31

S4 TI((communication or language or speech or learning) N5 disorder*) OR AB((communication or language or speech or learning) N5 disorder*) Limiters - Date Published: 20180201-20201231

Database - ERIC 275

S3 TI((cognitive* or learning or mobility or sensory or visual* or vision or sight or hearing or physical* or mental* or intellectual*) N2 (impair* or disabilit* or disabl* or handicap*)) OR AB((cognitive* or learning or mobility or sensory or visual* or vision or sight or hearing or physical* or mental* or intellectual*) N2 (impair* or disabilit* or disabl* or handicap*)) Limiters - Date Published: 20180201-20201231

Database - ERIC 1,222

S2 TI((physical* or intellectual* or learning or psychiatric* or sensory or motor or neuromotor or cognitive or mental* or developmental or communication or learning) N2 (disabilit* or disabl* or handicap*)) OR AB ((physical* or intellectual* or learning or psychiatric* or sensory or motor or neuromotor or cognitive or mental* or developmental or communication or learning) N2 (disabilit* or disabl* or handicap*)) Limiters - Date Published: 20180201-20201231

Database - ERIC 1,123

S1 TI((disable* or disabilit* or handicapped) N5 (person* or people or child* or adolescen* or women or mother* or maternal or group*)) OR AB ((disable* or disabilit* or handicapped) N5 (person* or people or child* or adolescen* or women or mother* or maternal or group*)) Limiters - Date Published: 20180201-20201231

Database - ERIC **889**

1. **Scopus – Searched 26^th^ February 2020**

( ( TITLE ( ( ( intellectual* OR educational* OR mental* OR psychological* OR developmental ) W/5 ( impair* OR retard* OR deficien* OR disable* OR disabili* OR handicap* OR ill* ) ) ) ) OR ( TITLE ( ( ( hearing OR acoustic OR ear* ) W/5 ( loss* OR impair* OR deficien* OR disable* OR disabili* OR handicap* ) ) OR deaf* ) ) OR ( TITLE ( ( ( ( visual* OR vision OR eye* OR ocular ) W/5 ( loss* OR impair* OR deficien* OR disable* OR disabili* OR handicap* ) ) OR blind* ) ) ) OR ( TITLE ( ( ( "cerebral pals*" OR "spina bifida" OR "muscular dystroph*" OR arthriti* OR "osteogenesis imperfecta" OR "musculoskeletal abnormalit*" OR "musculo-skeletal abnormalit*" OR "muscular abnormalit*" OR "skeletal abnormalit*" OR "limb abnormalit*" OR "brain injur*" OR amput* OR clubfoot OR polio* OR paraplegi* OR paralys* OR paralyz* OR hemiplegi* OR stroke* OR "cerebrovascular accident*" ) W/2 ( impair* OR disabilit* OR disabl* OR handicap* ) ) ) ) OR ( TITLE ( ( physical* W/5 ( impair* OR deficien* OR disable* OR disabili* OR handicap* ) ) ) ) OR ( ( TITLE ( ( ( disable* OR disabilit* OR handicapped ) W/5 ( person* OR people OR child* OR adolescen* OR women OR mother* OR maternal OR group* ) ) ) ) OR ( TITLE ( ( ( physical* OR intellectual* OR learning OR psychiatric* OR sensory OR motor OR neuromotor OR cognitive OR mental* OR developmental OR communication OR learning ) W/2 ( disabilit* OR disabl* OR handicap* ) ) ) ) OR ( TITLE ( ( ( cognitive* OR learning OR mobility OR sensory OR visual* OR vision OR sight OR hearing OR physical* OR mental* OR intellectual* ) W/2 ( impair* OR disabilit* OR disabl* OR handicap* ) ) ) ) OR ( TITLE ( ( ( communication OR language OR speech OR learning ) W/5 disorder* ) ) ) OR ( TITLE ( ( ( depression OR depressive OR anxiety OR psychiat* OR well-being OR "quality of life" OR self-esteem OR "self perception" ) W/2 ( impair* OR disabilit* OR disabl* OR handicap* ) ) ) ) OR ( TITLE ( "mental health" ) ) OR ( TITLE ( ( ( schizophreni* OR psychos* OR psychotic OR schizoaffective OR schizophreniform OR dementia* OR alzheimer* ) W/2 ( impair* OR disabilit* OR disabl* OR handicap* ) ) ) ) OR ( TITLE ( ( ( mental* OR emotional* OR psychiatric OR neurologic* ) W/2 ( disorder* OR ill OR illness* ) ) ) ) OR ( TITLE ( ( autis* OR dyslexi* OR "Down* syndrome" OR mongolism OR "trisomy 21" ) ) ) ) ) AND ( TITLE-ABS-KEY ( afghanistan OR albania OR algeria OR angola OR argentina OR armenia OR armenian OR aruba OR azerbaijan OR bangladesh OR benin OR byelarus OR byelorussian OR belarus OR belorussian OR belorussia OR belize OR bhutan OR bolivia OR bosnia OR herzegovina OR hercegovina OR botswana OR brasil OR brazil OR bulgaria OR "Burkina Faso" OR "Burkina Fasso" OR "Upper Volta" OR burundi OR urundi OR cambodia OR "Khmer Republic" OR kampuchea OR cameroon OR cameroons OR cameron OR camerons OR "Cape Verde" OR "Central African Republic" OR chad OR china OR colombia OR comoros OR "Comoro Islands" OR comores OR mayotte OR congo OR zaire OR "Costa Rica*" OR "Cote d'Ivoire" OR "Ivory Coast" OR cuba OR djibouti OR "French Somaliland" OR dominica OR "Dominican Republic" OR "East Timor" OR "East Timur" OR "Timor Leste" OR ecuador OR egypt OR "United Arab Republic" OR "El Salvador" OR eritrea OR ethiopia OR fiji OR gabon OR "Gabonese Republic" OR gambia OR gaza OR "Georgia Republic" OR "Georgian Republic" OR ghana OR grenada OR guatemala OR guinea OR guiana OR guyana OR haiti OR honduras OR india OR maldives OR indonesia OR iran OR iraq OR jamaica OR jordan OR kazakhstan OR kazakh OR kenya OR kiribati OR korea OR kosovo OR kyrgyzstan OR kirghizia OR "Kyrgyz Republic" OR kirghiz OR kirgizstan OR "Lao PDR" OR laos OR lebanon OR lesotho OR basutoland OR liberia OR libya OR macedonia OR madagascar OR "Malagasy Republic" OR malaysia OR malaya OR malay OR sabah OR sarawak OR malawi OR mali OR "Marshall Islands" OR mauritania OR mauritius OR "Agalega Islands" OR mexico OR micronesia OR "Middle East" OR moldova OR moldovia OR moldovian OR mongolia OR montenegro OR morocco OR ifni OR mozambique OR myanmar OR myanma OR burma OR namibia OR nepal OR "Netherlands Antilles" OR "New Caledonia" OR nicaragua OR niger OR nigeria OR pakistan OR palau OR palestine OR panama OR paraguay OR peru OR philippines OR philipines OR phillipines OR phillippines OR "Puerto Ric*" OR romania OR rumania OR roumania OR rwanda OR ruanda OR "Saint Lucia" OR "St Lucia" OR "Saint Vincent" OR "St Vincent" OR grenadines OR samoa OR "Samoan Islands" OR "Navigator Island" OR "Navigator Islands" OR "Sao Tome" OR senegal OR serbia OR montenegro OR seychelles OR "Sierra Leone" OR "Sri Lanka" OR "Solomon Islands" OR somalia OR "South Africa" OR sudan OR suriname OR surinam OR swaziland OR syria OR tajikistan OR tadzhikistan OR tadjikistan OR tadzhik OR tanzania OR thailand OR togo OR togolese AND republic OR tonga OR tunisia OR turkey OR turkmenistan OR turkmen OR uganda OR ukraine OR uzbekistan OR uzbek OR vanuatu OR "New Hebrides" OR venezuela OR vietnam OR "Viet Nam" OR "West Bank" OR yemen OR yugoslavia OR zambia OR zimbabwe OR "Developing Countries" OR africa OR asia OR caribbean OR "West Indies" OR "South America" OR "Latin America" OR "Central America" OR ( ( developing OR "less* developed" OR "under developed" OR underdeveloped OR "middle income" OR "low* income" OR underserved OR "under served" OR deprived OR poor* ) W/1 ( countr* OR nation* OR population* OR world ) ) OR ( ( developing OR "less* developed" OR "under developed" OR underdeveloped OR "middle income" OR "low* income" ) W/1 ( economy OR economies ) ) OR ( low* W/1 ( gdp OR gnp OR "gross domestic" OR "gross national" ) ) OR ( low W/3 middle W/3 countr* ) OR lmic OR lmics OR "third world" OR "lami countr*" OR "transitional countr*" ) ) AND ( ( TITLE-ABS-KEY ( ( ( systematic* OR synthes* ) W/3 ( research OR evaluation* OR finding* OR thematic* OR report OR descriptive OR explanatory OR narrative OR meta* OR review* OR data OR literature OR studies OR evidence OR map OR quantitative OR study OR studies OR paper OR impact OR impacts OR effect* OR compar* ) ) ) ) OR ( TITLE-ABS-KEY ( ( "meta regression" OR "meta synth*" OR "meta-synth*" OR "meta analy*" OR "metaanaly*" OR "meta-analy*" OR "metanaly*" OR "metaregression" OR "metaregression" OR "methodologic* overview" OR "pool* analys*" OR "pool* data" OR "quantitative* overview" OR "research integration" ) ) ) OR ( TITLE-ABS-KEY ( ( review W/3 ( effectiveness OR effects OR systemat* OR synth* OR integrat* OR map* OR methodologic* OR quantitative OR evidence OR literature ) ) ) ) OR ( TITLE-ABS-KEY ( ( "meta ethnograph*" OR "meta synthesis" OR ( synthesis AND ( "qualitative literature" OR "qualitative research" ) ) OR "critical interpretive synthesis" OR ( "systematic review" AND ( "qualitative research" OR "qualitative literature" OR "qualitative stud*" ) ) OR "thematic synthesis" OR "framework synthesis" OR "realist review" OR "realist synthesis" OR "qualitative systematic review*" OR "qualitative evidence synthes*" OR ( ( "quality assessment" OR "critical appraisal" OR "literature search*" ) AND ( "qualitative research" OR "qualitative literature" OR "qualitative stud*" ) ) OR ( noblit AND hare ) OR "meta narrative*" OR "narrative synthesis" ) ) ) OR ( TITLE-ABS-KEY ( ( random$ OR placebo$ OR "single blind$" OR "double blind$" OR "triple blind$" OR cohort$ OR ( ( case$ OR cohort OR "follow up" OR follow-up ) W/2 ( control$ OR series OR report$ OR study OR studies ) ) OR retrospective$ OR ( observ$ W/3 ( study OR studies ) ) ) ) ) ) AND ( LIMIT-TO ( PUBYEAR , 2020 ) OR LIMIT-TO ( PUBYEAR , 2019 ) OR LIMIT-TO ( PUBYEAR , 2018 ) ) - **339**

1. **Web of Science (Social Sciences Citation Index) – Searched 26^th^ February 2020**

# 30 1,524

#29 AND #23 AND #15

Indexes=SSCI Timespan=2018-2020

# 29 129,728

#28 OR #27 OR #26 OR #25 OR #24

# 28 82,678

TS=(random$ or placebo$ or "single blind$" or "double blind$" or "triple blind$" or cohort$ or ((case$ or cohort or "follow up" or follow-up) NEAR/2 (control$ or series or report$ or study or studies)) or retrospective$ or (observ$ NEAR/3 (study or studies)))

# 27 2,265

TS=("meta ethnograph*" or "meta synthesis" or (synthesis and ("qualitative literature" or "qualitative research")) or "critical interpretive synthesis" or ("systematic review" and ("qualitative research" or "qualitative literature" or "qualitative stud*")) or "thematic synthesis" or "framework synthesis" or "realist review" or "realist synthesis" or "qualitative systematic review*" or "qualitative evidence synthes*" or (("quality assessment" or "critical appraisal" or "literature search*") and ("qualitative research" or "qualitative literature" or "qualitative stud*")) or (Noblit and Hare) or "meta narrative*" or "narrative synthesis")

# 26 32,626

TS=(review NEAR/3 (effectiveness or effects or systemat* or synth* or integrat* or map* or methodologic* or quantitative or evidence or literature))

# 25 25,301

TS=("meta regression" or "meta synth*" or "meta-synth*" or "meta analy*" or "metaanaly*" or "meta-analy*" or "metanaly*" or "metaregression" or "metaregression" or "methodologic* overview" or "pool* analys*" or "pool* data" or "quantitative* overview" or "research integration")

# 24 26,382

TS=((systematic* or synthes*) NEAR/3 (research or evaluation* or finding* or thematic* or report or descriptive or explanatory or narrative or meta* or review* or data or literature or studies or evidence or map or quantitative or study or studies or paper or impact or impacts or effect* or compar*))

# 23 218,259

#22 OR #21 OR #20 OR #19 OR #18 OR #17 OR #16

# 22 1,125

TS=(lmic or lmics or "third world" or "lami countr*" or "transitional countr*")

# 21 2,953

TS=(low NEAR/3 middle NEAR/3 countr*)

# 20 286

TS=(low* NEAR (gdp or gnp or "gross domestic" or "gross national"))

# 19 2,100

TS=((developing or "less* developed" or "under developed" or underdeveloped or "middle income" or "low* income") NEAR (economy or economies))

# 18 15,712

TS=((developing or "less* developed" or "under developed" or underdeveloped or "middle income" or "low* income" or underserved or "under served" or deprived or poor*) NEAR/1 (countr* or nation? or population? or world or state*))

# 17 207,412

TS=((Afghanistan or Albania or Algeria or Angola or Argentina or Armenia or Armenian or Aruba or Azerbaijan or Bahrain or Bangladesh or Benin or Byelarus or Byelorussian or Belarus or Belorussian or Belorussia or Belize or Bhutan or Bolivia or Bosnia or Herzegovina or Hercegovina or Botswana or Brasil or Brazil or Bulgaria or "Burkina Faso" or "Burkina Fasso" or "Upper Volta" or Burundi or Urundi or Cambodia or "Khmer Republic" or Kampuchea or Cameroon or Cameroons or Cameron or Camerons or "Cape Verde" or "Central African Republic" or Chad or China or Colombia or Comoros or "Comoro Islands" or Comores or Mayotte or Congo or Zaire or "Costa Rica*" or "Cote d'Ivoire" or "Ivory Coast" or Cuba or Djibouti or "French Somaliland" or Dominica or "Dominican Republic" or "East Timor" or "East Timur" or "Timor Leste" or Ecuador or Egypt or "United Arab Republic" or "El Salvador" or Eritrea or Ethiopia or Fiji or Gabon or "Gabonese Republic" or Gambia or Gaza or "Georgia Republic" or "Georgian Republic" or Ghana or Grenada or Guatemala or Guinea or Guiana or Guyana or Haiti or Honduras or Hungary or India or Maldives or Indonesia or Iran or Iraq or Jamaica or Jordan or Kazakhstan or Kazakh or Kenya or Kiribati or Korea or Kosovo or Kyrgyzstan or Kirghizia or "Kyrgyz Republic" or Kirghiz or Kirgizstan or "Lao PDR" or Laos or Lebanon or Lesotho or Basutoland or Liberia or Libya or Macedonia or Madagascar or "Malagasy Republic" or Malaysia or Malaya or Malay or Sabah or Sarawak or Malawi or Mali or "Marshall Islands" or Mauritania or Mauritius or "Agalega Islands" or Mexico or Micronesia or "Middle East" or Moldova or Moldovia or Moldovian or Mongolia or Montenegro or Morocco or Ifni or Mozambique or Myanmar or Myanma or Burma or Namibia or Nepal or "Netherlands Antilles" or "New Caledonia" or Nicaragua or Niger or Nigeria or Muscat or Pakistan or Palau or Palestine or Panama or Paraguay or Peru or Philippines or Philipines or Phillipines or Phillippines or "Puerto Ric*" or Romania or Rumania or Roumania or Rwanda or Ruanda or "Saint Lucia" or "St Lucia" or "Saint Vincent" or "St Vincent" or Grenadines or Samoa or "Samoan Islands" or "Navigator Island" or "Navigator Islands" or "Sao Tome" or Senegal or Serbia or Montenegro or Seychelles or "Sierra Leone" or "Sri Lanka" or "Solomon Islands" or Somalia or "South Africa" or Sudan or Suriname or Surinam or Swaziland or Syria or Tajikistan or Tadzhikistan or Tadjikistan or Tadzhik or Tanzania or Thailand or Togo or Togolese Republic or Tonga or Tunisia or Turkey or Turkmenistan or Turkmen or Uganda or Ukraine or Uzbekistan or Uzbek or Vanuatu or "New Hebrides" or Venezuela or Vietnam or "Viet Nam" or "West Bank" or Yemen or Yugoslavia or Zambia or Zimbabwe) NOT ("African-American*" OR "African-American*" OR "Mexican American*" OR "American Indian*" OR "Asian American*" OR "native american*")) OR CU=((Afghanistan or Albania or Algeria or Angola or Argentina or Armenia or Armenian or Aruba or Azerbaijan or Bahrain or Bangladesh or Benin or Byelarus or Byelorussian or Belarus or Belorussian or Belorussia or Belize or Bhutan or Bolivia or Bosnia or Herzegovina or Hercegovina or Botswana or Brasil or Brazil or Bulgaria or "Burkina Faso" or "Burkina Fasso" or "Upper Volta" or Burundi or Urundi or Cambodia or "Khmer Republic" or Kampuchea or Cameroon or Cameroons or Cameron or Camerons or "Cape Verde" or "Central African Republic" or Chad or China or Colombia or Comoros or "Comoro Islands" or Comores or Mayotte or Congo or Zaire or "Costa Rica*" or "Cote d'Ivoire" or "Ivory Coast" or Cuba or Djibouti or "French Somaliland" or Dominica or "Dominican Republic" or "East Timor" or "East Timur" or "Timor Leste" or Ecuador or Egypt or "United Arab Republic" or "El Salvador" or Eritrea or Ethiopia or Fiji or Gabon or "Gabonese Republic" or Gambia or Gaza or "Georgia Republic" or "Georgian Republic" or Ghana or Grenada or Guatemala or Guinea or Guiana or Guyana or Haiti or Honduras or Hungary or India or Maldives or Indonesia or Iran or Iraq or Jamaica or Jordan or Kazakhstan or Kazakh or Kenya or Kiribati or Korea or Kosovo or Kyrgyzstan or Kirghizia or "Kyrgyz Republic" or Kirghiz or Kirgizstan or "Lao PDR" or Laos or Lebanon or Lesotho or Basutoland or Liberia or Libya or Macedonia or Madagascar or "Malagasy Republic" or Malaysia or Malaya or Malay or Sabah or Sarawak or Malawi or Mali or "Marshall Islands" or Mauritania or Mauritius or "Agalega Islands" or Mexico or Micronesia or "Middle East" or Moldova or Moldovia or Moldovian or Mongolia or Montenegro or Morocco or Ifni or Mozambique or Myanmar or Myanma or Burma or Namibia or Nepal or "Netherlands Antilles" or "New Caledonia" or Nicaragua or Niger or Nigeria or Muscat or Pakistan or Palau or Palestine or Panama or Paraguay or Peru or Philippines or Philipines or Phillipines or Phillippines or "Puerto Ric*" or Romania or Rumania or Roumania or Rwanda or Ruanda or "Saint Lucia" or "St Lucia" or "Saint Vincent" or "St Vincent" or Grenadines or Samoa or "Samoan Islands" or "Navigator Island" or "Navigator Islands" or "Sao Tome" or Senegal or Serbia or Montenegro or Seychelles or "Sierra Leone" or "Sri Lanka" or "Solomon Islands" or Somalia or "South Africa" or Sudan or Suriname or Surinam or Swaziland or Syria or Tajikistan or Tadzhikistan or Tadjikistan or Tadzhik or Tanzania or Thailand or Togo or Togolese Republic or Tonga or Tunisia or Turkey or Turkmenistan or Turkmen or Uganda or Ukraine or Uzbekistan or Uzbek or Vanuatu or "New Hebrides" or Venezuela or Vietnam or "Viet Nam" or "West Bank" or Yemen or Yugoslavia or Zambia or Zimbabwe) NOT ("African-American*" OR "African-American*" OR "Mexican American*" OR "American Indian*" OR "Asian American*" OR "native american*"))

# 16 31,104

TS=(Africa or Asia or Caribbean or "West Indies" or "Middle East" or "South America" or "Latin America" or "Central America") or CU=(Africa or Asia or Caribbean or "West Indies" or "Middle East" or "South America" or "Latin America" or "Central America")

# 15 31,763

#14 OR #13 OR #12 OR #11 OR #10 OR #9 OR #8 OR #7 OR #6 OR #5 OR #4 OR #3 OR #2 OR #1

# 14 408

TI=(physical* NEAR/5 (impair* or deficien* or disable* or disabili* or handicap*))

# 13 110

TI=(("cerebral pals*" or "spina bifida" or "muscular dystroph*" or arthriti* or "osteogenesis imperfecta" or "musculoskeletal abnormalit*" or "musculo-skeletal abnormalit*" or "muscular abnormalit*" or "skeletal abnormalit*" or "limb abnormalit*" or "brain injur*" or amput* or clubfoot or polio* or paraplegi* or paralys* or paralyz* or hemiplegi* or stroke* or "cerebrovascular accident*") NEAR/2 (impair* or disabilit* or disabl* or handicap*))

# 12 1,845

TI=(((visual* or vision or eye* or ocular) NEAR/5 (loss* or impair* or deficien* or disable* or disabili* or handicap*)) or blind*)

# 11 1,194

TI=(((hearing or acoustic or ear*) NEAR/5 (loss* or impair* or deficien* or disable* or disabili* or handicap*)) or deaf*)

# 10 4,909

TI=((intellectual* or educational* or mental* or psychological* or developmental) NEAR/5 (impair* or retard* or deficien* or disable* or disabili* or handicap* or ill*))

# 9 7,175

TI=(autis* or dyslexi* or "Down* syndrome" or mongolism or "trisomy 21")

# 8 4,557

TI=((mental* or emotional* or psychiatric or neurologic*) NEAR/2 (disorder* or ill or illness*))

# 7 378

TI=((schizophreni* or psychos* or psychotic or schizoaffective or schizophreniform or dementia* or alzheimer*) NEAR/2 (impair* or disabilit* or disabl* or handicap*))

# 6 9,358

TI=("mental health")

# 5 1,830

TS=((depression or depressive or anxiety or psychiat* or well-being or "quality of life" or self-esteem or "self perception") NEAR/2 (impair* or disabilit* or disabl* or handicap*))

# 4 506

TI=((communication or language or speech or learning) NEAR/5 disorder*)

# 3 5,906

TI=((cognitive* or learning or mobility or sensory or visual* or vision or sight or hearing or physical* or mental* or intellectual*) NEAR/2 (impair* or disabilit* or disabl* or handicap*))

# 2 3,336

TI=((physical* or intellectual* or learning or psychiatric* or sensory or motor or neuromotor or cognitive or mental* or developmental or communication or learning) NEAR/2 (disabilit* or disabl* or handicap*))

# 1 2,568

TI=((disable* or disabilit* or handicapped) NEAR/5 (person* or people or child* or adolescen* or women or mother* or maternal or group*))

1. **WHO Global Health Index (2016-2020) – Searched 26^th^ February 2020**

**Total after duplicates removed: 1054**

Search 1: tw: (((disable* OR disabilit* OR handicapped OR "mental health" OR impair*) AND ((systematic* OR synthes*) AND (research OR evaluation* OR finding* OR thematic* OR report OR descriptive OR explanatory OR narrative OR meta* OR review* OR data OR literature OR studies OR evidence OR map OR quantitative OR study OR studies OR paper OR impact OR impacts OR effect* OR compar*)))) AND (instance:"ghl") AND ( db:("LILACS" OR "WPRIM" OR "WHOLIS" OR "IMEMR" OR "AIM") AND mj:("Mental Health" OR "Disabled Persons" OR "Hearing Loss" OR "Quality of Life" OR "Mental Disorders" OR "Intellectual Disability" OR "Disability Evaluation" OR "Mental Health Services") AND year_cluster:("2020" OR "2019" OR "2018" OR "2017" OR "2016"))

Search 2: tw:((disable* OR disabilit* OR handicapped OR "mental health" OR impair*) AND (review AND (effectiveness OR effects OR systemat* OR synth* OR integrat* OR map* OR methodologic* OR quantitative OR evidence OR literature)) ) AND (instance:"ghl") AND ( db:("LILACS" OR "WPRIM" OR "WHOLIS" OR "IMEMR" OR "AIM") AND mj:("Mental Health" OR "Disabled Persons" OR "Hearing Loss" OR "Quality of Life" OR "Mental Disorders" OR "Intellectual Disability" OR "Disability Evaluation" OR "Mental Health Services") AND year_cluster:("2020" OR "2019" OR "2018" OR "2017" OR "2016"))

Search 3: tw:((disable* OR disabilit* OR handicapped OR "mental health" OR impair*) AND (random* OR placebo* OR "single blind*" OR "double blind*" OR "triple blind*" OR cohort* )) AND (instance:"ghl") AND ( db:("LILACS" OR "WPRIM" OR "WHOLIS" OR "IMEMR" OR "AIM") AND mj:("Mental Health" OR "Disabled Persons" OR "Hearing Loss" OR "Quality of Life" OR "Mental Disorders" OR "Intellectual Disability" OR "Disability Evaluation" OR "Mental Health Services") AND year_cluster:("2020" OR "2019" OR "2018" OR "2017" OR "2016"))

Search 4: tw:(((disable* OR disabilit* OR handicapped OR "mental health" OR impair*) AND ("meta regression" OR "meta synth*" OR meta-synth* OR "meta analy*" OR metaanaly* OR meta-analy* OR metanaly*) )) AND (instance:"ghl") AND ( db:("LILACS" OR "WPRIM" OR "WHOLIS" OR "IMEMR" OR "AIM") AND mj:("Mental Health" OR "Disabled Persons" OR "Hearing Loss" OR "Quality of Life" OR "Mental Disorders" OR "Intellectual Disability" OR "Disability Evaluation" OR "Mental Health Services") AND year_cluster:("2020" OR "2019" OR "2018" OR "2017" OR "2016"))

Search 5: tw:((disable* OR disabilit* OR handicapped OR "mental health" OR impair*) AND (((case* OR cohort OR "follow up" OR follow-up) AND (control* OR series OR report* OR study OR studies)) OR retrospective* OR (observ* AND (study OR studies)))) AND (instance:"ghl") AND ( db:("LILACS" OR "WPRIM" OR "WHOLIS" OR "IMEMR" OR "AIM") AND mj:("Mental Health" OR "Disabled Persons" OR "Hearing Loss" OR "Quality of Life" OR "Mental Disorders" OR "Intellectual Disability" OR "Disability Evaluation" OR "Mental Health Services") AND year_cluster:("2020" OR "2019" OR "2018" OR "2017" OR "2016"))
